# A deep mutational scanning platform to characterize the fitness landscape of anti-CRISPR proteins

**DOI:** 10.1093/nar/gkae1052

**Published:** 2024-11-18

**Authors:** Tobias Stadelmann, Daniel Heid, Michael Jendrusch, Jan Mathony, Sabine Aschenbrenner, Stéphane Rosset, Bruno E Correia, Dominik Niopek

**Affiliations:** Center for Synthetic Biology, Technical University of Darmstadt, Darmstadt 64287, Germany; Department of Biology, Technical University of Darmstadt, Darmstadt 64287, Germany; Hochschule Offenburg, Faculty of Mechanical & Process Engineering, 77652 Offenburg, Germany; Hochschule Offenburg, Faculty of Mechanical & Process Engineering, 77652 Offenburg, Germany; European Molecular Biology Laboratory (EMBL), 69117 Heidelberg, Germany; European Molecular Biology Laboratory (EMBL), 69117 Heidelberg, Germany; Institute of Pharmacy and Molecular Biotechnology (IPMB), Faculty of Engineering Sciences, Heidelberg University, Heidelberg 69120, Germany; Institute of Pharmacy and Molecular Biotechnology (IPMB), Faculty of Engineering Sciences, Heidelberg University, Heidelberg 69120, Germany; Institute of Bioengineering, École Polytechnique Fédérale de Lausanne, Lausanne, CH-1015, Switzerland; Institute of Bioengineering, École Polytechnique Fédérale de Lausanne, Lausanne, CH-1015, Switzerland; Institute of Pharmacy and Molecular Biotechnology (IPMB), Faculty of Engineering Sciences, Heidelberg University, Heidelberg 69120, Germany

## Abstract

Deep mutational scanning is a powerful method for exploring the mutational fitness landscape of proteins. Its adaptation to anti-CRISPR proteins, which are natural CRISPR-Cas inhibitors and key players in the co-evolution of microbes and phages, facilitates their characterization and optimization. Here, we developed a robust anti-CRISPR deep mutational scanning pipeline in *Escherichia coli* that combines synthetic gene circuits based on CRISPR interference with flow cytometry coupled sequencing and mathematical modeling. Using this pipeline, we characterized comprehensive single point mutation libraries for AcrIIA4 and AcrIIA5, two potent inhibitors of CRISPR-Cas9. The resulting mutational fitness landscapes revealed considerable mutational tolerance for both Acrs, suggesting an intrinsic redundancy with respect to Cas9 inhibitory features, and – for AcrIIA5 – indicated mutations that boost Cas9 inhibition. Subsequent *in vitro* characterization suggested that the observed differences in inhibitory potency between mutant inhibitors were mostly due to changes in binding affinity rather than protein expression levels. Finally, to demonstrate that our pipeline can inform Acrs-based genome editing applications, we employed a selected subset of mutant inhibitors to increase CRISPR-Cas9 target specificity by modulating Cas9 activity. Taken together, our work establishes deep mutational scanning as a powerful method for anti-CRISPR protein characterization and optimization.

## Graphical Abstract

**Graphical Abstract:**
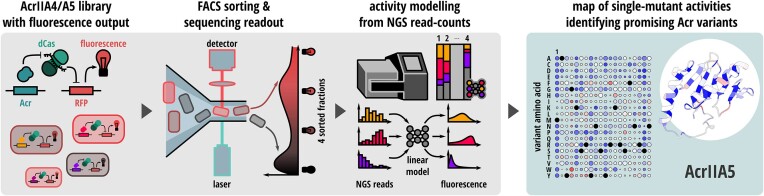

## Introduction

Deep mutational scanning (DMS) leverages next-generation sequencing (NGS) to rapidly measure the effects of mutations on proteins, enabling the creation of comprehensive mutational fitness landscapes that reveal functional and structural properties, informing protein optimization and engineering (1). In general, DMS requires (i) a robust pipeline for generating a mutant library, (ii) a coupled genotype-phenotype platform and corresponding assay to separate active from inactive protein variants and (iii) an NGS-based readout to assess the enrichment of variants in the active and/or inactive variant pools.

In the past, DMS has been applied to characterize human disease mutations (2), dissect protein-coding regions in the adeno-associated virus genome (3) and investigate the mutational fitness landscape of Cas9 (4,5). Moreover, comprehensive DMS datasets comprising protein single and double mutants were used for detailed analysis of intramolecular interactions, based on which the three-dimensional protein structure could be inferred (6). DMS was also used to analyze the impact of mutations in the SARS-CoV-2 receptor binding domain on the binding of ACE2, a central host factor required for virus entrance (7). Moreover, mutational scanning approaches have been utilized for characterizing human caspases (8) as well as to study essential bacterial proteins and inform antibiotics development (9). Together, these examples illustrate the power of mutational scanning for protein functional dissection and structure-mechanistic analysis.

Anti-CRISPR (Acr) proteins are natural inhibitors of CRISPR-Cas systems (10,11) and play an important role in the co-evolution of phages and their bacterial hosts (12). Hundreds of experimentally validated anti-CRISPR proteins have been described and many thousands have been additionally predicted (13). Together, these Acrs target all common class II CRISPR Cas effectors, including type II CRISPR-Cas9 systems (14–23), as well as Cas12a, a type V CRISPR effector (24,25), and Cas13, a type VI CRISPR effector (26,27).

AcrIIA4 was amongst the first type II-A Acrs to be reported, discovered in *Listeria monocytogenes* (21). It is rather selective for Cas9 from *Streptococcus pyogenes* (*Spy*) and binds the Cas9-sgRNA complex with nanomolar affinity (28), acting as a protospacer adjacent motif (PAM) mimic to prevent Cas9 DNA binding. AcrIIA4 also interferes with the catalytic RuvC domain of Cas9 (29–31). Like many Acrs, AcrIIA4 functions effectively when heterologously expressed in various cellular contexts, including human, plant and yeast cells (32,33).

AcrIIA5 is a special member of the type II-A clade, discovered in a virulent *Streptococcus thermophilus* phage (16). In contrast to the target-selective AcrIIA4, AcrIIA5 inhibits a broad spectrum of CRISPR-Cas9 systems, including type II-A, II-B and II-C Cas9 orthologs (22,23). Structurally, AcrIIA5 features an α/β fold linked to an N-terminal intrinsically disordered region (IDR), which was proposed to modulate its association with the Cas9-sgRNA complex and is essential for its inhibitory activity (34). However, the precise mechanism of action of AcrIIA5 remains to be fully elucidated. While inhibition of Cas9 nuclease activity is considered the primary mode of action, (34) there is ongoing debate about whether AcrIIA5 also interferes with Cas9 DNA target binding (22,23). Additionally, inconsistencies exist in the literature regarding its *in vitro* and *in vivo* activity (22,34) (see ‘Discussion’ section). The strong inhibitory potency of AcrIIA4 on *Spy*Cas9, the most widely used Cas9 ortholog, and the broad-spectrum activity of AcrIIA5, render these Acrs particularly valuable candidates for controlling, fine-tuning, and safeguarding Cas9 activity across various applications (see below).

Since Acrs play a central role in the evolutionary battle between phages and their microbial hosts (12,35–37), they are likely under evolutionary pressure to (i) effectively inhibit the CRISPR-mediated immune response of the host and (ii) rapidly adapt to evolutionary changes in their target Cas effectors and external factors such as temperature, pH and cell context. This would require substantial plasticity at the Acr sequence as well as structural level.

Apart from the relevance of Acrs to micro- and phage biology, their small size and often robust CRISPR inhibitory function across a broad range of cell types render them highly attractive tools for CRISPR-Cas control in gene editing applications. Acrs have been employed, for instance, as parts in CRISPR-Cas-based gene circuits (38), shown to enable reduction of off-target editing via temporally confined and fine-tuned Cas9 activity (30,39), and coupled to exogenous or endogenous stimuli such as light (40–42), chemicals (43) or microRNAs (44–46) for inducible and cell-type restrictive genome editing.

Adapting DMS to Acrs could not only yield mutational fitness landscapes that provide important insights into their evolutionary design space and plasticity, but also inform mechanistic studies of newly identified Acrs. Moreover, Acr mutational fitness landscapes could guide the engineering of customized Acrs with altered or improved inhibition potency or specificity (44). Finally, the ability to selectively alter regions of Acr polypeptides by mutagenesis without perturbing their actual function could be important for their *in vivo* application (46), i.e. to overcome potential issues of immunogenicity similar to previous efforts for CRISPR nucleases (47–49).

Here, we present a robust experimental and computational pipeline to examine the mutational fitness landscape of anti-CRISPR proteins via DMS. By applying our pipeline to AcrIIA4 (21) and AcrIIA5 (16), we created comprehensive datasets on the impact of individual amino acid exchanges on CRISPR-Cas9 inhibition as well as the overall mutation tolerance for these two widely used and mechanistically and structurally divergent Acrs. We then aligned our DMS data with known sequence, structural and evolutionary features to identify the key factors influencing mutational tolerance. We show that AcrIIA5 naturally is a weak inhibitor of Cas9 DNA binding, a property that can be considerably improved by several, independent single-point mutations. AcrIIA4, in turn, effectively blocks CRISPRi in its wild-type form, while mutations either preserve or attenuate its activity, e.g. by reducing its affinity for Cas9-sgRNA complexes. Combining a selected set of new AcrIIA4 mutants with our previously established approach of CRISPR-Cas9 fine-tuning via genetic Acr fusion (39) resulted in markedly improved CRISPR-Cas9 target specificity. Our work establishes mutational scanning as a powerful strategy for Acr characterization and engineering.

## Material and methods

### Molecular cloning

All plasmids used in this study are listed in Supplementary Table T1, sgRNA target sequences are shown in Supplementary Table T2. Sequences and maps for the CRISRPi selection vectors are available as Supplementary data 1 (Genbank files).

Oligonucleotides and synthetic double-stranded DNA fragments were obtained from Integrated DNA Technologies (IDT). Polymerase chain reactions (PCRs) were performed with Q5 Hot Start High Fidelity Polymerase (New England Biolabs) or Invitrogen Platinum Superfi II DNA Polymerase (Thermo Fisher Scientific). Expression vectors were created using classical restriction enzyme cloning or Golden-Gate assembly (50). Restriction enzymes were obtained from New England Biolabs, T4 DNA ligase was obtained from Jena Biosciences. Agarose gel electrophoresis was used to analyze PCR and restriction products. Bands of the expected size were cut out and DNA was extracted with a ZymoClean Gel DNA recovery kit (Zymo Research). For all cloning steps, chemically competent *Escherichia coli* K12 DH5α cells (New England Biolabs) were used. Antibiotics were used at the following concentrations: carbenicillin, 50 μg mL^−1^; chloramphenicol, 25 μg mL^−1^; kanamycin, 50 μg mL^−1^. Plasmid DNA was purified with the ZR Plasmid Miniprep or ZymoPure II Midiprep kit (both Zymo Research). The integrity of all plasmids was verified by Sanger Sequencing (GENEWIZ Europe).

AcrIIA4 (*Listeria monocytogenes)* (21) and AcrIIA5 (Phage D4276) (16) encoding DNA sequences were codon optimized for expression in *E. coli*, obtained as double-stranded DNA fragments (IDT) and cloned into pBAD24 (pBAD24-sfGFPx1 was a gift from Sankar Adhya & Francisco Malagon, Addgene plasmid #51 558) (51) via unique EcoRI and HindIII restriction sites, resulting in the plasmids pBAD24-AcrIIA4 and pBAD24-AcrIIA5.

To construct plasmids co-expressing d*Spy*Cas9 and sgRNA scaffolds, a second multiple cloning site was introduced into pBbA5 (pBbA5c-RFP was a gift from Jay Keasling, Addgene plasmid #35 281) (52) by PCR, resulting in pBbA5C_sgMCS. A fragment encoding an *E. coli* codon optimized d*Spy*Cas9 was PCR amplified from vector pdCas9 (gift from Luciano Marraffini, Addgene plasmid #46 569) (53) and cloned into pBbA5C_sgMCS using EcoRI and BamHI restriction sites, thereby generating pBbA5C_sgMCS_d*Spy*Cas9. A sgRNA expression cassette with an red fluorescent protein (RFP) reporter-targeting spacer sequence was ordered as overlapping DNA oligonucleotides, extended by PCR and cloned into pBbA5c-spCas9 using NotI and SalI restriction sites, resulting in pBbA5C_sgMCS_d*Spy*Cas9_RFP_guide.

To generate the RFP reporter plasmid, a bacterial codon optimized RFP encoding sequence was PCR amplified from pBbA5c-RFP; the Anderson Promoter J23102 (http://parts.igem.org/Promoters/Catalog/Anderson) was included in the 5′-extension of the forward primer. A sequence encoding a SsrA degradation tag (54) (AANDENYADAS; corresponding to part BBa_M0052 in iGEM parts registry - parts.igem.org) was included as 5′-extension into the reverse primer to reduce the half-live of RFP in bacterial cells. The resulting PCR fragments were cloned into pJUMP27 (pJUMP27-1AsfGFP was a gift from Chris French, Addgene plasmid #126 974) (55) via XbaI and PstI restriction sites, thereby resulting in pJUMP27_J23102_RFP_M0052.

The single codon mutational libraries of AcrIIA4 and AcrIIA5 were generated by back-to-back PCR on pBAD24-AcrIIA4 and -AcrIIA5 with forward primers containing NNB overhangs as previously described (56). PCRs were performed for each position individually to avoid PCR bias, followed by gel extraction. The purified fragments were treated with KLD enzyme mix (New England Biolabs) and transformed into *E. coli* DH5a chemically competent cells. Cells were grown in LB supplemented with carbenicillin. Note that sub-libraries corresponding to a single codon were grown individually and to stationary phase in a 96 deep-well plate. Cultures were then combined at an equal volume and further grown in 50 mL LB carbenicillin until saturation, followed by extraction of plasmid DNA.

Single mutant Acr variants for bacterial validation experiments were created via back-to-back PCR on template vectors pBAD24-AcrIIA4 and -5 and by incorporating the mutations into the 5′-extension of one primer, followed by KLD treatment.

The vectors co-encoding firefly luciferase, a firefly luciferase gene targeting sgRNA and *Renilla* luciferase (for normalization purposes) used for the dual luciferase assay experiments in mammalian cells were previously reported by us (40). Vector pCMV-AcrIIA4 was previously reported by us (40). A human codon optimized AcrIIA5 coding sequence was obtained as double-stranded DNA fragment from IDT and cloned into pcDNA3.1, thereby creating pCMV-AcrIIA5. Single codon substitutions were introduced into the Acr expression constructs via back-to-back PCR by incorporating the mutations into the 5′-extension of one primer, followed by KLD treatment. Constructs expressing Cas9-Acr genetic fusions were created on the basis of a Cas9-AcrIIA4 fusion construct (Addgene #128 115) previously reported by us (39). Briefly, AcrIIA4 mutants were PCR-amplified from the corresponding CMV-driven constructs (see above) and cloned into Cas9-AcrIIA4 via BamHI/HindIII restriction sites to generate vectors encoding genetic fusions of different AcrIIA4 variants to the Cas9 C-terminus (Cas-Acr). Vectors for human cell expression of *Spy*Cas9 (Addgene #113 033) and a CCR5 locus targeting sgRNA (Addgene #113 041) were previously reported by us (40). An all-in-one vector co-expressing both *Spy*Cas9 and the CCR5 sgRNA was created by PCR-amplifying the sgRNA expression cassette from the CCR5 sgRNA construct with primers carrying MluI/XbaI sites, followed by cloning the PCR amplicon into the *Spy*Cas9 expression construct via MluI/SpeI.

### Transformation of *E. coli* with Acr libraries

For library generation, chemically competent bacterial cells were first co-transformed with the d*Spy*Cas9 plasmid and the RFP reporter plasmid and selected on LB containing kanamycin and chloramphenicol. An overnight culture of a single bacterial colony was inoculated in Super Optimal Broth medium supplemented with kanamycin and chloramphenicol, grown to an optical density (OD)_600_ of 0.5 and chemical competent cells were prepared according to the Inoue transformation protocol (57). AcrIIA4 and AcrIIA5 libraries were transformed into chemical competent cells by heat-shock at 42°C for 1 min. 0.1% of the total transformation volume was plated on LB Agar plates containing carbenicillin, chloramphenicol and kanamycin to estimate the transformation efficiency and corresponding library complexity (Supplementary Tables T3 and T4). The remaining cells were grown in liquid LB medium containing carbenicillin, chloramphenicol and kanamycin until stationary phase, followed by cryopreservation in aliquots.

### Fluorescence-activated cell sorting

Cryopreserved cells were thawed and grown in LB medium containing carbenicillin, chloramphenicol, kanamycin until stationary phase, followed by induction with 1 mM IPTG and 4 mM arabinose at a starting OD_600_ of 0.05. Following a 14-h incubation period, cells were collected by centrifugation at 5000 × g for 10 min, the supernatant was removed, and cells were resuspended in 10 volumes of 1 × phosphate-buffered saline (PBS) buffer.

Fluorescence-activated cell sorting (FACS) experiments were performed on a Sony SH800S cell sorter at the Offenburg University of Applied Sciences. *E. coli c*ells were first gated using the forward-scatter area and site-scatter area. Bacterial cells were then sorted into four fractions according to their RFP intensity using a total of 1 000 000 cells per fraction. Collected cells were either frozen for DNA extraction followed by deep amplicon sequencing or recovered by growing the cells in LB medium containing the necessary antibiotics for subsequent characterization of fluorescence enrichments. In the latter case, cells were washed in PBS twice and grown in LB medium containing carbenicillin, chloramphenicol and kanamycin overnight. The cultures form the individual fractions were then induced with 1 mM IPTG and 4 mM arabinose at a starting OD_600_ of 0.05 and analyzed on the same Sony SH800S cell sorter 14 h later with the same settings as described above.

### Amplicon deep sequencing

FACS-sorted *E. coli* cell fractions were lysed and DNA extracted for subsequent first stage PCR amplification of AcrIIA4 or AcrIIA5 genes with primers containing Nextera XT index overhangs. PCR amplicons were purified by gel extraction and the concentrations of each sample was adjusted to 25 ng/μl for the second stage barcoding PCR using TG Nextera XT Index Kit v2 Set A (Illumina) according to the manufacturer's protocol. Libraries were sequenced at the EMBL Heidelberg GeneCore facility on the Illumina MiSeq system using 2 × 250 paired-end sequencing reagents (MiSeq Reagent Kit v2, 500-cycles).

### NGS data analysis

Paired-end reads for each sorted fraction were assembled by their overlap and filtered to remove reads corresponding to sequences not contained in the original library. Only reads corresponding the wild-type Acr and reads containing mutations in *exactly one* codon relative to wild-type were kept for further analysis. Read counts of the remaining reads were augmented with pseudocounts and normalized within each sorting fraction. Then, for each single mutant, read counts were normalized across fractions, resulting in a distribution of read counts over fractions for each mutant.

### Acr mutant activity regression

As a proxy for Acr activity, linear regression of the binned distribution and mean of log fluorescence intensities was performed for a set of single Acr mutants (benchmarks). To this end, log fluorescence intensity data from flow cytometry for the benchmark mutant set were binned (20 equally spaced bins, range from 0 to 14) and bins normalized to sum to one. An affine regression model relating the read distribution across sorting fractions to the distribution of log fluorescence intensity was zero initialized and fit using gradient descent to minimize squared error under L² regularization. Model fits were evaluated using mean square error calculated via leave-one-out cross-validation on the set of benchmark mutants. All models were fit to three biological replicates for each of the Acr mutants in the training set.

Log fluorescence intensity distributions were predicted for all individual Acr mutants. As a measure of confidence in the predicted distribution, the standard deviation of the predicted mean log fluorescence intensity was calculated from three biological replicates. High standard deviation indicates low confidence in the predicted mean log fluorescence intensity.

### Protein production for Acr single point mutants

Bacterial codon-optimized sequences of AcrIIA4 mutants were PCR amplified and cloned into pET-28b(+) using BsaI sites. Constructs were expressed with an N-terminal His-tag and TEV cleavage site, resulting in a protein size of ∼12.3 kDa. Plasmids were transformed into BL21(DE3) competent cells (Thermo Fisher Scientific). Protein expression was conducted in LB medium supplemented with kanamycin, and cells were induced with 0.5 mM IPTG at an OD_600_ of 0.6, followed by incubation at 18°C for 16 h. Next, cells were harvested by centrifugation, resuspended in lysis buffer (50 mM Tris-HCL pH 7.5, 200 mM NaCl, 1 mM DTT, 1 mM PMSF and 0.3 mg ml^−1^ lysozyme) and sonicated. For protein expression analysis, the lysates were loaded on a sodiumdodecyl sulphate-polyacrylamide gel electrophoresis (SDS-PAGE) gel stained with Coomassie Blue. To purify the proteins, samples were centrifuged, and the cleared lysates were incubated with 1 mL HisPur Ni-NTA Resin (Thermo Fisher Scientific), washed and eluted. Eluates were desalted using 7K MWCO Zeba Spin Desalting columns, stored in PBS solution. The flow-through was collected and concentrated using 3K MWCO Pierce Protein Concentrators PES (Thermo Fisher Scientific), followed by flash freezing.

### Expression strength comparison

To compare the expression levels of AcrIIA4 wild-type and mutants, the pBAD24-AcrrIIA4 constructs were transformed into DH5a chemically competent cells. Note that in these constructs, AcrIIA4 is untagged, i.e. does not contain the N-terminal His-tag and TEV cleavage site mentioned above (protein size ∼10 kDa). Expression was performed in LB medium supplemented with carbenicillin and induced with 4 mM arabinose at a starting OD_600_ of 0.05. After 14 h of incubation, cell concentrations were normalized, cells were lysed by boiling and lysates were loaded onto a SDS-PAGE gel, followed by staining with Coomassie Blue.

### Circular Dichroism measurements

Far-UV circular dichroism spectra for AcrIIA4 wild-type and mutant proteins were collected from 190 to 250 nm using a Jasco J-815 circular dichroism spectrometer and a 1-mm path length quartz cuvette. Prior to measurement, proteins were suspended in 10 mM phosphate buffer at concentrations ranging from 20 to 40 μM. Wavelength spectra were obtained by averaging two scans at a scan rate of 20 nm per minute and a response time of 0.125 s. Thermal denaturation data were obtained by tracking the ellipticity shift at 220 nm from temperatures of 20–90°C using increments of 2–5°C.

### Affinity measurements

Biolayer interferometry (BLI) binding data were collected with a Gator Bioanalysis System and processed using the instrument's integrated software. For the binding assay, His tagged proteins were loaded onto anti-NTA biosensors (Gator probes) at 5 μg mL^−1^ in binding buffer (PBS pH 7.4) for 120 s. Homemade CRISPR-Cas9 RNP was diluted from 200 to 6.25 nM in binding buffer. After baseline measurement in the binding buffer alone, the binding kinetics were monitored by dipping the biosensors in wells containing the Cas9 RNP at the indicated concentration (association step) and then dipping the sensors back into baseline/buffer (dissociation).

### Mammalian cell experiments

HEK293T (human embryonic kidney) cells were cultured at 5% CO_2_ and 37°C in a humidified incubator and passaged every 2–3 days. Cells were maintained in DMEM (Thermo Fisher Scientific) supplemented with 10% (v/v) fetal calf serum (Thermo Fisher Scientific), 100 U mL^−1^ penicillin and 100 μg mL^−1^ streptomycin (Thermo Fisher Scientific). Prior to the assays, cells were checked for mycoplasma contamination by qPCR (Mycoplasma Check, GATC Eurofins).

A total of 12 500 cells were seeded in a 96-well culture plate and transfected the next day with Lipofectamine 3000 (Thermo Fisher Scientific) according to the manufacturer's protocol. For luciferase experiments, cells were co-transfected with 33 ng of (i) a plasmid co-expressing *Renilla* and firefly luciferase as well as a sgRNA targeting the firefly reporter gene, (ii) 33 ng of a plasmid encoding *Spy*Cas9 with C- and N-terminal nuclear localization signals and an N-terminal 3xFlag tag, (iii) and either 11, 33 or 99 ng of a vector encoding human codon-optimized Acr mutant variants. A stuffer plasmid (pUC19) was used to top up plasmid levels per sample to 165 ng, thereby keeping the total amount of DNA constant across all samples.

After 72 h post-transfection, cells were washed with PBS and firefly and *Renilla* luciferase activities were measured using the Dual-Glo luciferase assay system (Promega) according to manufacturer's instructions. First, one volume Dual-Glo reagent was added to each well. Following a 10-min incubation time, lysates were transferred to a white 96-well plate and firefly luciferase photon counts were measured using a FLUOstar Omega multimode reader (BMG Labtech). Subsequently, one volume Dual-Glo Stop & Glo was added to quench the firefly signal and activate *Renilla* luciferase, samples were incubated for 10 min, and *Renilla* luciferase photon counts were measured. To calculate the reported luciferase activity values, firefly luciferase photon counts were normalized to *Renilla* luciferase photon counts in each sample.

For analysis of ON- and OFF-target editing, sgRNAs and experimental procedures previously reported by us were employed (39). In brief, cells were co-transfected with (i) 66 ng of a construct encoding the respective Cas9-Acr fusion variant (or wild-type Cas9 as control), (ii) 66 ng of a vector encoding an sgRNA targeting the AAVS1 locus (Addgene construct #128 119) or the HEK locus (Addgene construct #128 123) and (iii) 66 ng of an irrelevant stuffer DNA. After 72 h post transfection, a region surrounding the ON-target (AAVS1: 5′-GGGAGGGAGAGCTTGGCAGG**GGG**-3′; HEK: 5′-GGCACTGCGGCTGGAGGTGG**GGG**-3′; PAM-sequence is in bold) or relevant off-target sites (AAVS1 OFF-target: 5′- GGGAAGGGGAGCTTGGCAGG**TGG**-3′; HEK OFF-target: 5′- TGCACTGCGGCCGGAGGAGG**TGG**-3′) was PCR-amplified, followed by Sanger sequencing (Microsynth) and quantification of indel frequencies using TIDE (58).

For analysis of CCR5 locus editing in the presence of AcrIIA5 mutants, HEK293T cells were co-transfected with (i) 100 or 190 ng of a vector co-expressing Cas9 and a CCR5 locus targeting sgRNA, and (ii) 100 or 10 ng of the respective AcrIIA5 variant using Lipofectamine 2000 (Thermo Fisher Scientific) and according to the manufacturer's protocol. After 72 h post-transfection, a region surrounding the expected Cas9 cut site was PCR amplified with primes carrying Illumina sequencing adapters (CCR5_fw: 5′-ACACTCTTTCCCTACACGACGCTCTTCCGATCTcattgcttggccaaaaagagag-3′; CCR5_re: 5′- GACTGGAGTTCAGACGTGTGCTCTTCCGATCTgaaggaaaaacaggtcagag-3′) as well as barcodes for sample multiplexing, followed by sequencing via the Genewiz Amplicon-EZ sequencing service.

## Results

### Implementation and validation of an anti-CRISPR deep mutational scanning pipeline

**Figure 1.**
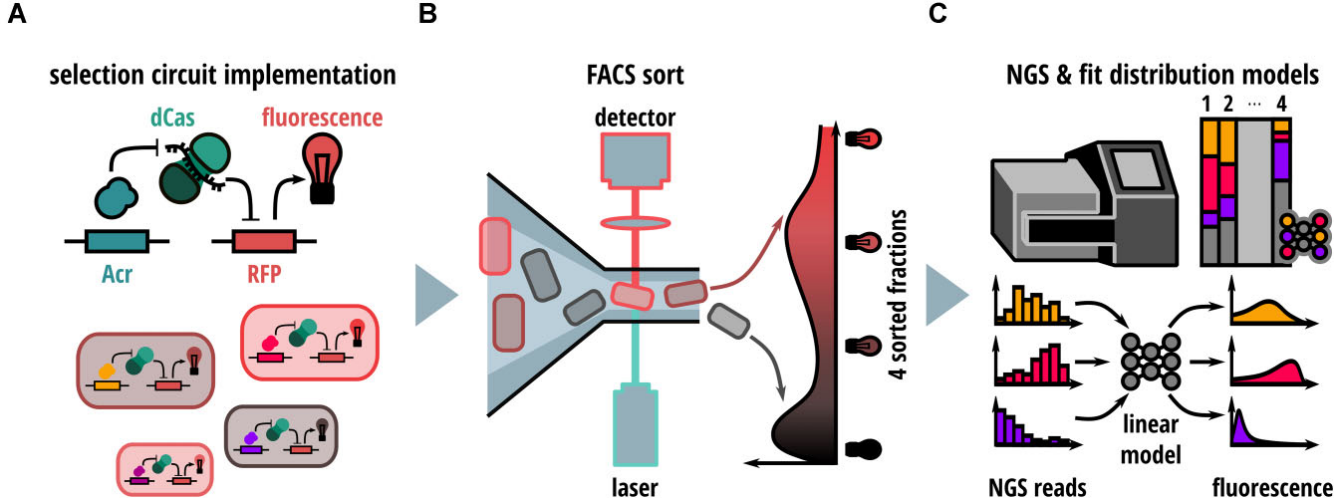
Implementation of a DMS pipeline for anti-CRISPR proteins. General setup of the DMS workflow comprising (**A**) a CRISPRi-based selection circuit in *E. coli* that generates a fluorescent output indicative of Acr activity, (**B**) a Flow-seq pipeline consisting of enrichment of Acr mutants with distinct activity by FACS, (**C**) followed by NGS, and a data analysis pipeline to predict inhibition potencies for each Acr mutant.

Our anti-CRISPR DMS pipeline comprises four steps: (1) generation of the Acr mutational library; (2) Implementation of a CRISPR interference (CRISPRi)-based gene circuit that can be inhibited by an Acr, hence enabling selection of Acrs mutants according to their activity; (3) FACS-mediated fractionation of the mutational library based on the intensity of a CRISPRi-dependent fluorescent reporter signal and deep amplicon sequencing of the individual fractions; (4) data analysis and integration using a mathematical model trained on a set of benchmark mutants (Figure 1).

**Figure 2.**
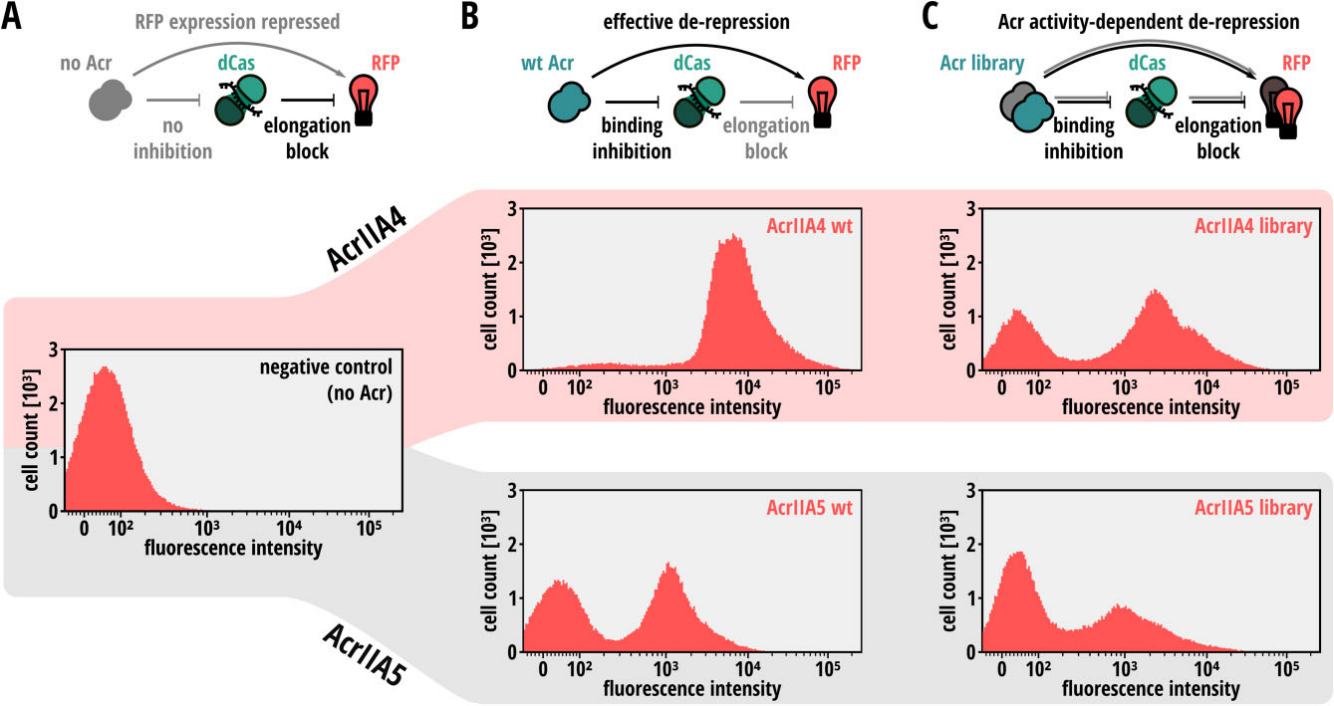
Validation of the bacterial CRISPRi gene circuit. *E. coli* carrying plasmids expressing an RFP reporter, d*Spy*Cas9 and an RFP gene-targeting sgRNA were transformed with (**A**) a control vector containing no Acr, (**B**) with a vector expressing wild-type AcrIIA4 or AcrIIA5 or (**C**) with the AcrIIA4 and AcrIIA5 mutant libraries covering all possible single point mutations, followed flow cytometry analysis. Top: Schematics of the expected CRISPRi circuit behavior. Bottom: Histograms showing the RFP fluorescence distribution in the cell population. wt, wild-type.

First, to implement a robust gene circuit for Acr selection, we created a three-plasmid system encoding (i) a (mutant) Acr protein, (ii) a catalytically impaired *Spy*Cas9 (d*Spy*Cas9) and sgRNA and (iii) a red fluorescent protein (RFP) reporter (Figure 2). The sgRNA was designed to direct the d*Spy*Cas9-sgRNA ribonucleoprotein complex to the 5′ region of the RFP reporter non-template strand, thereby efficiently blocking transcriptional elongation through CRISPRi (Figure 2A), as previously described (59). In presence of an Acr that prevents *Spy*Cas9 from binding its target DNA, RFP expression is restored, as we determined by flow cytometry analysis (Figure 2B). While it is well established that AcrIIA4 abolishes Cas9 DNA binding (21,29,31), the ability of AcrIIA5 to block DNA targeting by Cas9 has been under debate (22,23). When tested in context of our *E. coli* CRISPRi selection circuit, however, also AcrIIA5 showed a considerable rescue of reporter expression as compared to the CRISPRi negative control (compare Figure 2A and B). This indicates that AcrIIA5 is capable of interfering with Cas9 DNA binding under our experimental conditions. Notably, while the fluorescence histogram for AcrIIA4 showed a single peak shifted towards higher-fluorescent values compared to the no Acr control, AcrIIA5 expression resulted in a bimodal fluorescence distribution (compare Figure 2A and B; see ‘Discussion’ section for details).

To create libraries covering all single point mutants for AcrIIA4 and -5, we performed PCRs with primer pairs that replace each individual codon with NNB randomized codons. Due to the small size of Acrs typically around ∼100 amino acids, this straight-forward procedure is both rapid and cost-efficient. We first cloned the single codon sub-libraries into expression plasmids, then mixed the resulting plasmid sub-libraries at equimolar ratios, followed by transformation into *E. coli* DH5α receiver cells already carrying the CRISPRi selection plasmids encoding dCas9, the sgRNA and the RFP reporter. From the transformed cells, cultures were grown to stationary phase, diluted and Acr and Cas9 expression was induced with arabinose and IPTG, respectively, followed by flow cytometry. The resulting *E. coli* AcrIIA4 and -5 libraries showed a broad distribution with respect to the RFP reporter fluorescence, indicating that they contained Acrs of various strengths (Figure 2C). Notably, the AcrIIA5 library sample showed a marked right-shift of the high-fluorescence peak compared to the wild-type AcrIIA5 control (Supplementary Figure S1), suggesting that the library contained some AcrIIA5 variants that inhibit Cas9 DNA binding more efficiently than the wild-type Acr.

**Figure 3.**
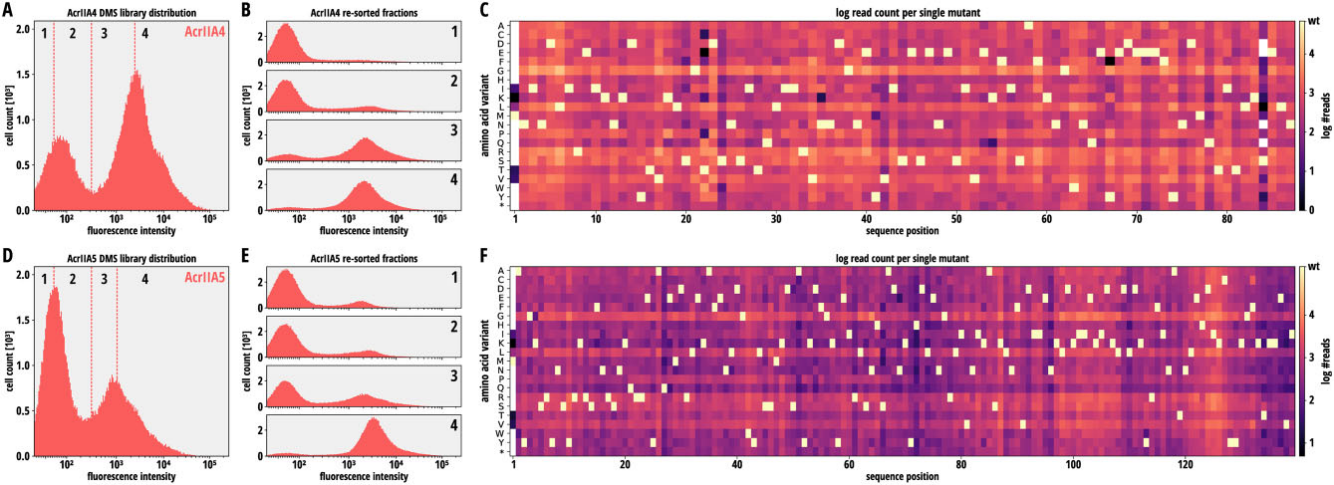
Library sorting and library coverage analysis by NGS. (**A,D**) Four fractions corresponding to the indicated bins were selected for AcrIIA4 (**A**) and AcrIIA5 (**D**) libraries. 1 000 000 cells per fraction were sorted. (**B, E**) For each sorted fraction from the AcrIIA4 (**B**) and AcrII5 (**E**) libraries, cultures were re-grown and -induced overnight individually, followed by flow cytometry analysis. (**C, F**) Library coverage for each single mutant in the AcrIIA4 (**C**) and AcrIIA5 (**F**) libraries. Logarithmic total read counts across all fractions and replicates are shown for each single mutant.

We then performed flow-sorting of the libraries in three biological replicates and collected a total of four fractions covering the entire fluorescence spectrum. 1 000 000 cells were sorted for each fraction of the AcrIIA4 (Figure 3A) and AcrIIA5 (Figure 3D) libraries. To determine whether sorted fractions indeed carried Acr variants of differing strength, we re-grew and induced cultures from each individual fraction separately and measured RFP expression by flow cytometry. We observed distinct fluorescence distributions across the four analyzed fractions for both Acr libraries (Figure 3B and E), suggesting that the different fractions were enriched for Acrs with distinct inhibition potency. We then sequenced the Acr mutant pools in all individual fractions on the Illumina MiSeq platform.

To analyze general mutant coverage, we first summed up all NGS reads in all fractions and calculated the proportion of reads corresponding to any given mutant (Figure 3C, AcrIIA4; Figure 3F, AcrIIA5). Overall, 92.8 and 99.4% of all possible AcrIIA4 and -5 mutants could be robustly detected within the library (>10 reads), respectively. The majority of mutants were covered by more than 100 reads.

**Figure 4.**
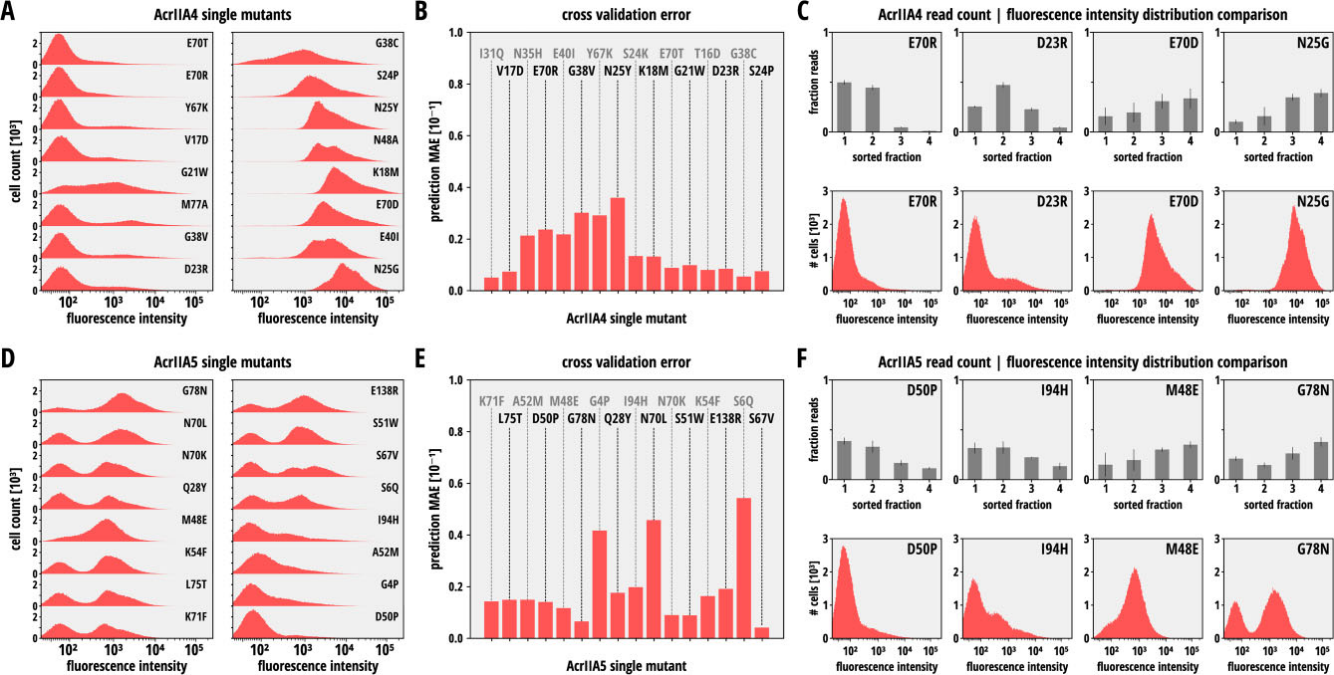
Single Acr mutant cross-validation and activity prediction. (**A, D**) Cross-validation of NGS data. 16 AcrIIA4 (**A**) and AcrIIA5 (**D**) mutants were cloned individually and their inhibition potency was assessed using the CRISPRi circuit and flow cytometry as readout. Mutants showing different levels of Cas9 inhibition strengths according to the NGS data were selected for the cross-validation. (**B, E**) Leave-one-out cross-validation error for predicting Acr mutant activities from NGS read distribution across the four FACS-sorted fractions for the 16 AcrIIA4 (**B**) and AcrIIA5 (**E**) mutants. (**C, F**) Exemplary NGS read distribution for AcrIIA4 (**C**) and AcrIIA5 (**F**) mutants across sorted fractions versus corresponding fluorescence histograms from individual flow cytometry measurements.

Next, to determine the inhibition potency for each Acr mutant in the library, we calculated the frequency of NGS reads corresponding to each variant in each fraction. To validate the read distribution across the four analyzed fractions, 16 individually measured benchmark mutants for each, AcrIIA4 (Figure 4A) and AcrIIA5 (Figure 4D), were used (see Material & Methods for details). These mutants were selected so that the diversity of observed NGS read count distributions was covered.

Next, we applied a regression model to the NGS read distributions (see Supplementary data 2–4). The model comprised a fully parameterized affine transformation and was trained on NGS read frequencies and the corresponding FACS fluorescence profiles of the individual Acr mutants. We then used the trained model to predict RFP fluorescence profiles corresponding to each Acr mutant within our *E. coli* library from the individual NGS read profiles (see Supplementary data 5 and 6 for the predicted fluorescence profile for every single AcrIIA4 and -5 mutant, respectively). Leave-one-out cross-validation on the benchmark mutant dataset (Figure 4B and E) revealed that our model could accurately infer the reporter fluorescence distribution (Figure 4C and F), and hence the inhibitory activity of AcrIIA4 and -5 mutants. From the predicted fluorescence distribution, we finally calculated the log fluorescence intensity, corresponding to the model-predicted ability of the Acr variant to prevent CRISPRi as a measure for the Acr inhibition potency.

To cross-validate our analysis, we compared the obtained Acr inhibition potencies (log mean fluorescence values) with two previously reported, smaller AcrIIA4 mutant datasets (29,33). Despite the fact that the readouts underlying these studies were profoundly different from our DMS readout, namely measurement of *Spy*Cas9 gene drive activity in *Saccharomyces cerevisiae* in case of Basgall et al. (33) and *in vitro* DNA cleavage in case of the Dong et al. study (29), our DMS data correlated with both literature datasets reasonably well (R = 0.91 and 0.78 for the Basgall et al and Dong et al datasets, respectively; Supplementary Figure S2).

On top of predicting the Acr activity from the NGS profiles, we also calculated the standard deviation of predicted activity values across the three biological replicates as a measure of confidence. Thus, for each mutant in our library, we obtained two values, i.e. the predicted activity and the confidence in that prediction.

### Anti-CRISPR mutational fitness landscapes dissect mutational sensitivity and reveal activity-enhancing mutations

**Figure 5.**
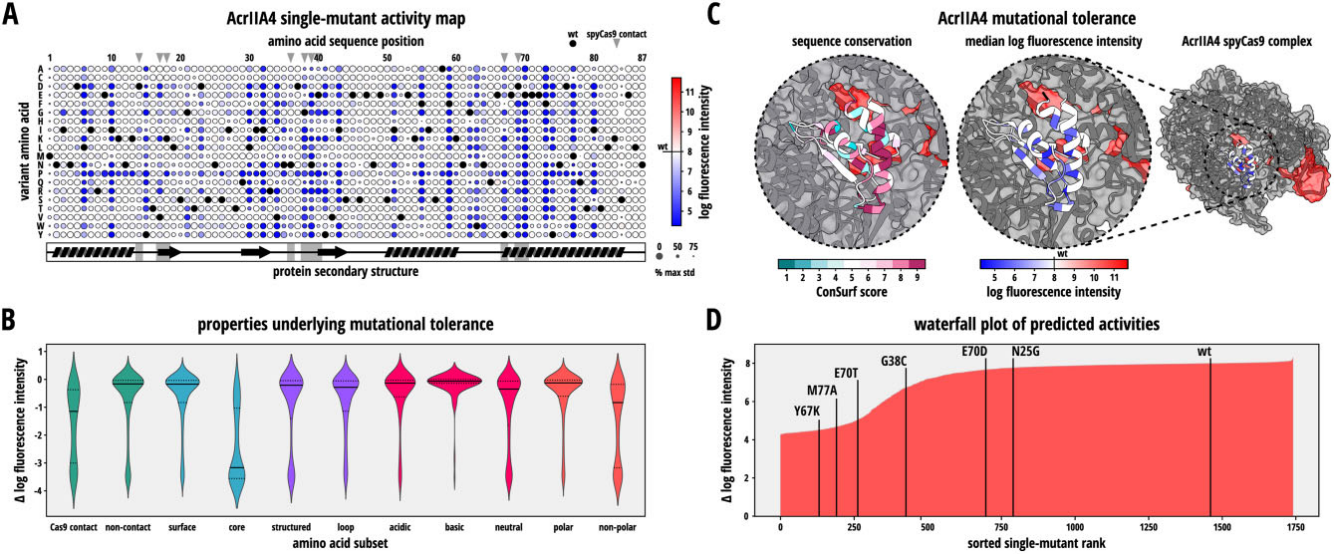
The AcrIIA4 mutational fitness landscape reveals high mutational tolerance. (**A**) AcrIIA4 mutational fitness landscape. Circle color indicates the inhibitory potency of the indicated mutant. Circle size corresponds to the percentage of the maximum standard deviation of the activities determined for each AcrIIA4 mutant in the dataset. Larger circles correspond to smaller standard deviations and therefore more precise values. Black circles correspond to the wild-type residue. Gray triangles and gray regions in the secondary structure cartoon below the heat map indicate residues that directly interact with Cas9. (**B**) Violin plots showing the distribution of the predicted log mean fluorescence values for the indicated residue subgroups in AcrIIA4. (**C**) Mean log intensity for each residue (right) and the sequence conservation (left) plotted on the crystal structure of AcrIIA4 in complex with Cas9 (PDB 5VW1). (**D**) Waterfall plot of predicted mutant activities, i.e. mean log fluorescence intensity. Several individually tested Acr mutants and the wild-type (wt) Acr are indicated.

**Figure 6.**
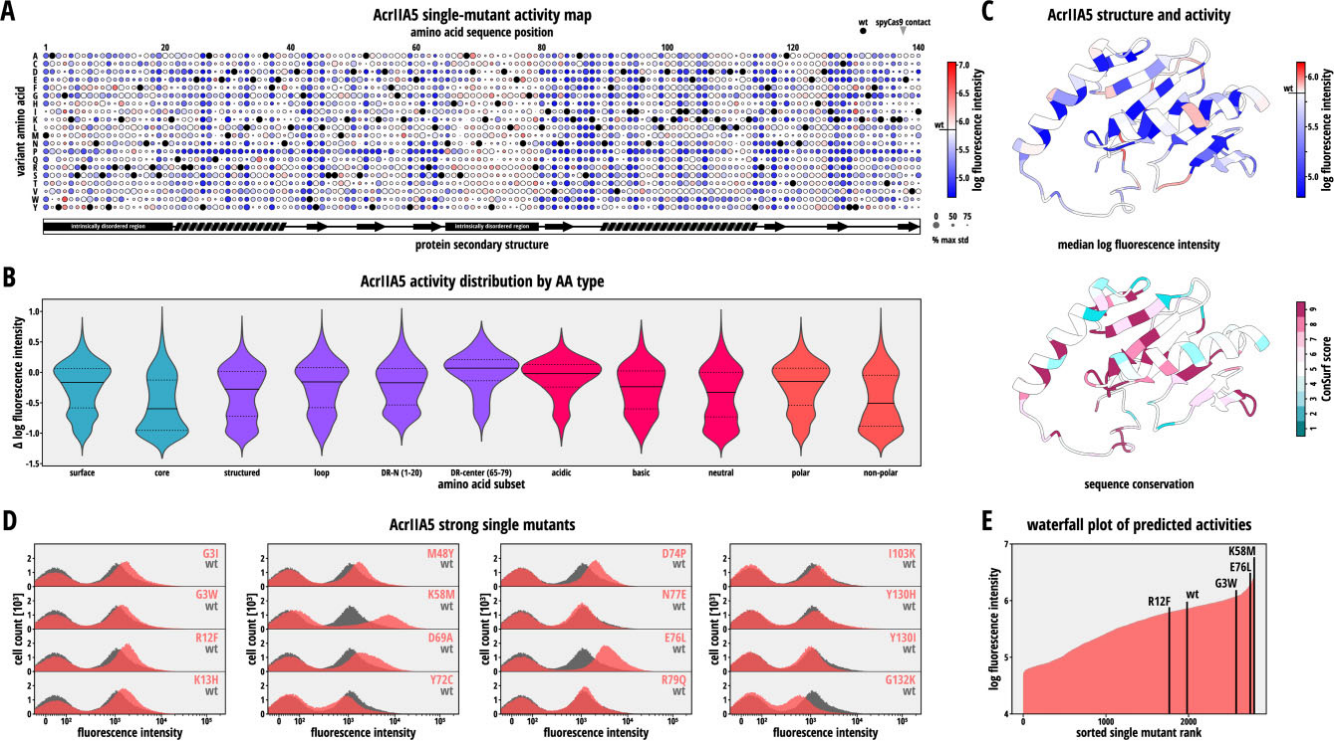
AcrIIA5 DMS reveals highly active mutants. (**A**) AcrIIA5 mutational fitness landscape. Circle size corresponds to the percentage of the maximum standard deviation of the activities determined for each AcrIIA5 mutant in the dataset. Larger circles correspond to smaller standard deviations and therefore more precise values. Black circles correspond to the wild-type residue. (**B**) Violin plots showing the distribution of the predicted log mean fluorescence values for the indicated residue subgroups and IDRs of AcrIIA5 single mutants. (**C**) Mean log intensity for each residue (top) and the sequence conservation (bottom) plotted on the NMR structure of AcrIIA5 (PDB 6LKF). (**D**) Histograms showing flow cytometry validation of several potent AcrIIA5 mutants (mutations are indicated) and their comparison to wild-type AcrIIA5. (**E**) Waterfall plot of predicted mutant activities, i.e. mean log fluorescence intensity. Several individually tested AcrIIA5 mutants and the wild-type (wt) AcrIIA5 are indicated.

From our analysis, we obtained comprehensive mutational fitness landscapes for both, AcrIIA4 (Figure 5A and Supplementary Figure S3A) and AcrIIA5 (Figure 6A and Supplementary Figure S3B).

Generally, AcrIIA4 and -5 both well-tolerate single point mutations at most positions throughout the protein (Figure 5B-D and Figure 6B,C). Around 70.1 and 71.3% of the mutant AcrIIA4 and -5 variants, respectively, showed an inhibitory potency equivalent to at least 90% of their cognate wild-type Acr.

In case of AcrIIA4, mutational tolerance was rather independent of whether mutations were introduced into loop regions or structured regions and also mostly independent of the chemical properties of the wild-type amino acid underlying the mutated residue (polar, acidic and basic) (Figure 5B, Supplementary Figure S4A). Mutations were generally less-well tolerated at the protein core as compared to the Acr surface (Figure 5B and C). However, Acr surface sites directly contacting Cas9 were rather mutation intolerant, which is expected considering their key role in the Acr–Cas9 interaction.

The AcrIIA5 DMS data set was noisier as compared to the AcrIIA4 data as evident from the overall lower confidence (i.e. higher standard deviation) of predicted activities (Figure 6A) as well as lower correlation between chemically similar amino acid substitutions (Supplementary Figure S4B). For a subset of AcrIIA5 mutants in our library, however, we could still confidently measure their activity (Figure 6A). Compared to AcrIIA4, AcrIIA5 shows an overall reduced tolerance for substitutions (Figure 6B), with the notable exception of the unstructured region from residue position 66–79 which showed a comparatively high mutation tolerance (Figure 6A and C). This mobile loop between beta sheets β3-β4 has previously been hypothesized to play a role in the binding of AcrIIA5 to Cas9, but has been suggested to be of varying importance dependent on the specific Cas9 ortholog (22). Of note, the N-terminal disordered region (IDR) described by An et al. (34) as critical for AcrIIA5 function was indeed more sensitive to point mutations than the β3-β4 mobile loop (see ‘Discussion’ section).

For both AcrIIA4 and AcrIIA5, mutant activity showed a weak correlation with the ConSurf score, a measure of sequence conservation (Figures 5C and 6C and Supplementary Figure S5; AcrIIA4: R = -0.52 with *P* = 3.08e-7; AcrIIA5: R = -0.37 with *P* = 7.91e-6).

Remarkably, the AcrIIA5 dataset also included several mutants that showed an activity higher than the wild-type Acr (Figure 6D and E; e.g. AcrIIA5 E76L and K58M). This suggests that unlike AcrIIA4, which is naturally very effective in preventing Cas9 DNA binding, the ability of AcrIIA5 to inhibit Cas9 DNA binding is naturally rather weak, but can be enhanced by mutagenesis. Taken together, our DMS analysis of AcrIIA4 and -5 revealed that both CRISPR inhibitors exhibit a certain degree of sequence plasticity.

### Acr mutant activity corresponds with Cas9 binding affinity *in vitro* and can inform genome editing applications in human cells

**Figure 7.**
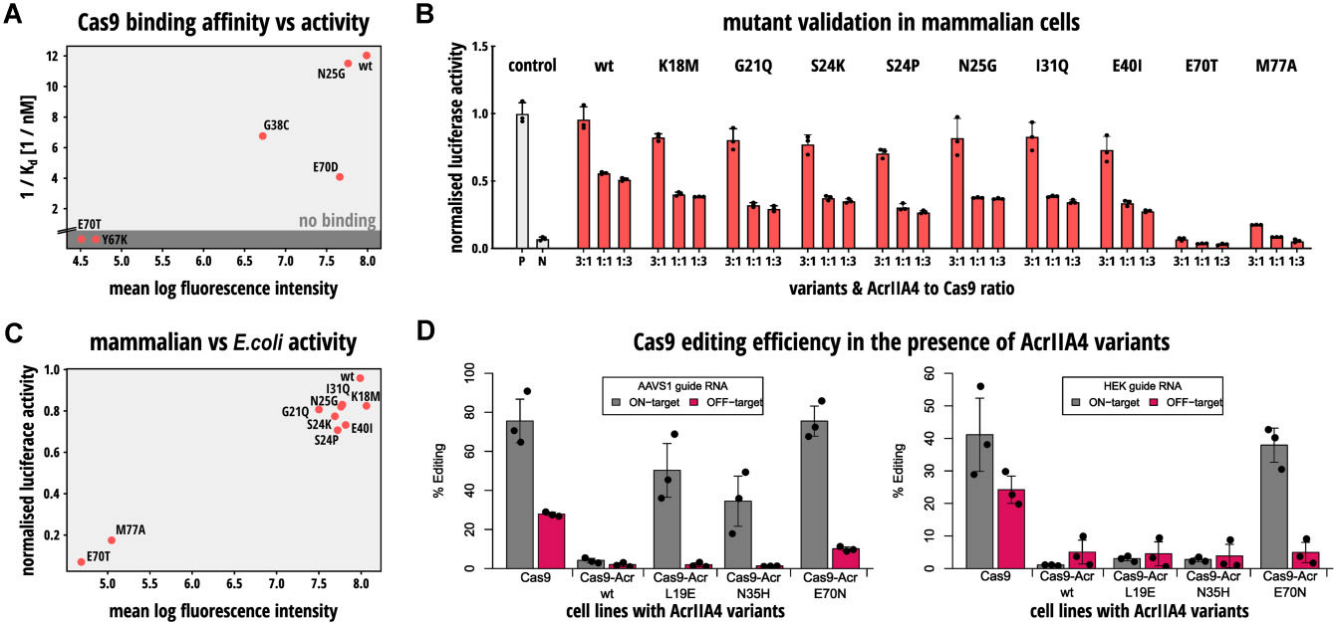
*In vitro* affinity measurements and human cell application of AcrIIA4 mutants derived from DMS. (**A**) Scatter plot of predicted log mean fluorescence values in Figure 5A and corresponding Cas9 binding affinities as measured by BLI. (**B**) Cross-validation of inhibition potency for individual AcrIIA4 mutants in mammalian cells. HEK293T cells were co-transfected with constructs expressing (i) *Renilla and* firefly luciferase as well as a sgRNA targeting the firefly reporter gene, (ii) *Spy*Cas9 and (iii) the indicated AcrIIA4 variant, followed by luciferase assay. The Cas9:Acr vector mass ratios used for transfection are indicated. Bars represent means, error bars the standard deviation and dots individual data points from *n* = 3 independent experiments. P: positive control, i.e. reporter with sgRNA. N: negative control, i.e. reporter with sgRNA + Cas9 (**C**) Scatter plot of predicted log mean fluorescence values in Figure 5A and corresponding luciferase activities in **B** (for the Acr:Cas9 ratio of 3:1). (**D**) Fusing Cas9 to mutant AcrIIA4 variants improves genome editing specificity. Cells were co-transfected with plasmids encoding Cas-Acr variants based on the indicated AcrIIA4 mutant (or wild-type AcrIIA4 as control) and an sgRNA targeting the AAVS1 (left) or HEK (right) locus. Following incubation for 72 hours, indel frequencies at the target and respective, prominent off-target sites were assessed using TIDE sequencing analysis. Bars represent means, error bars the standard deviation and dots individual data points from n = 3 independent experiments. (**A–D**) wt, wild-type AcrIIA4.

Next, to determine whether the mutation-induced modulation of Cas9 inhibition was related to changes in Cas9 binding affinity, we purified wild-type AcrIIA4 as well as five AcrIIA4 mutants of varying activity from our library (N25G, G38C, Y67K, E70D and E70T; Supplementary Figure S6). Circular dichroism measurements showed that the purified proteins were generally well-folded, with spectra corresponding to mixed alpha and beta secondary structures (Supplementary Figure S7). We then determined the binding affinity of these Acr variants to Cas9 RNP using BLI and compared the obtained affinities with the inhibition potencies derived from our DMS analysis (Figure 7A, Supplementary Figure S8). While we were able to detect tight binding to Cas9 for high activity AcrIIA4 mutants, no binding was detected for Y67K and E70T, the two mutants with the lowest mean log fluorescence intensity in the affinity measurement test set (Figure 7A and Supplementary Figure S8). We also examined whether there were significant changes in the expression levels of the purified mutants after induction with arabinose. By lysing the cells post-production and loading equal amounts on a Coomassie-stained SDS-PAGE gel, we found no substantial variations in expression levels (Supplementary Figure S9). Taken together, these data suggest that for the AcrIIA4 mutants characterized above, the observed functional effects are mostly due to changes in Cas9 binding affinity rather than other properties such as changes in protein stability or expression levels.

Next, to assess whether our *E. coli* DMS pipeline can inform the selection of Acrs with defined inhibition strength for applications in mammalian cells, we human codon optimized and expressed a subset of our AcrIIA4 mutants in HEK293T cells alongside a luciferase reporter, Cas9, and a luciferase-targeting sgRNA. We then compared the resulting luciferase activities (Figure 7B) with the Acr potencies (log mean fluorescence intensities) as measured by DMS in *E. coli* (see Figure 5A). We found that AcrIIA4 mutants classified as active, moderately active, or inactive in our DMS analysis also showed – at the qualitative level - corresponding activities in the mammalian cell assay (Figure 7C). Complementarily, we assessed the ability of six AcrIIA5 mutants to inhibit editing of the endogenous CCR5 locus in HEK293T cells using NGS as readout. We observed that AcrIIA5 mutants displaying weak or strong activity in the *E. coli* CRISPRi assay also showed low or high potency in the mammalian cell experiment (Supplementary Figure S10).

Finally, we aimed to illustrate the utility of the DMS pipeline to inform genome editing applications, specifically to reduce Cas9-mediated off-target editing. To this end, we applied several newly obtained AcrIIA4 mutants of varying inhibitory potency from our DMS set to the Cas-Acr approach previously reported by us (39). The Cas-Acr strategy employs attenuated Acrs genetically fused to Cas9 to fine-tune Cas9 activity, thereby improving target specificity via an effect that we had previously termed ‘kinetic insulation’ (39). We transfected the different Cas-Acr constructs based on AcrIIA4 mutants L19E, N35H or E70N or wild-type AcrIIA4 as a control into HEK293T cells. We then compared the editing specificity mediated by two different, transfected sgRNAs for the respective ON-target (AAVS1 or HEK) as well as a prominent OFF-target site. For wild-type Cas9, we observed potent ON- and OFF-target editing, the latter reaching more than 20% for both tested sgRNAs. In contrast, the Cas9-Acr variant based on wild-type AcrIIA4 was rather inactive with ON- and OFF-target editing below 10% for both sgRNAs. Remarkably, the Cas-Acr fusion based on the E70N AcrIIA4 mutation showed a strong gain in specificity, i.e. OFF-target editing was reduced by around 50% (AAVS1) or 80% (HEK), without any noticeable loss in ON-target activity. While the Cas-Acr constructs based on the L19E and N35H AcrIIA4 variants were largely impaired when using the HEK-targeting sgRNA, they retained considerable ON-target activity for AAVS1 while reducing the OFF-target editing to background levels. Taken together, these data indicate that the mutational fitness landscapes resulting from our DMS analysis in *E. coli* can inform the selection of Acr mutants with desired properties for genome editing applications in human cells.

## Discussion

Here we present a powerful DMS pipeline to comprehensively sample the mutational fitness landscape of anti-CRISPR proteins and applied it to AcrIIA4 and -5, two prominent, functionally and mechanistically divergent inhibitors of CRISPR-Cas9.

We found that both Acrs were tolerant to single-residue substitutions over large portions of the protein. This is consistent with a study by Figueroa and colleagues on an unrelated anti-CRISPR (AcrIF7), for which 2/3 of single mutations tested *in vitro* were well-tolerated, despite considerable evolutionary conservation of several of these residues (37).

Interestingly, we observed that even residues that lie within known, mechanistically important Acr regions, e.g. the Cas9-contacting loops in AcrIIA4 (29,31) or the intrinsically disordered N-terminus of AcrIIA5 (34), still tolerate mutations, at least to some extent. This suggests a certain degree of ‘intrinsic redundancy’ with respect to the Acr-Cas effector contacts that form the basis of the Cas inhibitory activity of AcrIIA4 and -5.

On the one hand, this property may provide the necessary evolutionary flexibility, i.e. render Acrs plastic to facilitate their adaptation to changes in the cognate Cas effector structure, changes in the cellular context or external conditions that impact Acr folding (e.g. pH, temperature).

On the other hand, these observations are also relevant from a protein engineering and CRISPR application point of view. Since many Acrs robustly function across a wide range of cell types, they present interesting tools for controlling, fine-tuning and safeguarding the activity of CRISPR-Cas effectors used in biotechnology or medicine (60). Mutational fitness landscapes obtained by DMS will enable the modulation of Acrs with respect to their inhibitory potency and, potentially, their selectivity (44), thus facilitating Acr applications.

While a number of previous studies characterized mutant Acrs, including variants of AcrIIA4 (29,31,33) and AcrIIA5 (34), these analyses were either focused on a few, selected residues or limited types of substitution or truncations. In contrast, our Acr-DMS pipeline provides a detailed and differentiated picture of the Acr mutational fitness landscapes. We determined that mutation-sensitive residues in AcrIIA4 are mostly found in flexible loops directly contacting Cas9, or in the Acr protein core critical for proper folding. Of note, the AcrIIA4 mutational scan did not yield considerably improved variants (Figure 5D), suggesting that the ability of AcrIIA4 to interfere with Cas9 DNA binding is close to at least a local optimum on the sequence-fitness landscape.

In our mutational scanning experiments, we observed that the wild-type AcrIIA5 naturally is a weak inhibitor of Cas9 DNA binding. This is reflected in the bimodal fluorescence distribution observed in the CRISPRi experiments for AcrIIA5 (Figure 2B), suggesting that this Acr can only inhibit Cas9 DNA binding under certain conditions (e.g. specific stoichiometry or high Acr expression in single cells). This bimodality likely contributes to the less distinct NGS read distributions across the FACS-sorted fractions observed for AcrIIA5 (even for the wild-type or potent AcrIIA5 mutants) compared to AcrIIA4 (Supplementary data 2–4). Additional variability in the read count distributions may also arise from inherent NGS noise (see standard deviations in Figures 5A and 6A).

Remarkably, the interference with Cas9 DNA binding by AcrIIA5 can be substantially enhanced by specific single-point mutations, at least for the *Spy*Cas9 ortholog employed as the Acr target in this work (Figure 6A and D). Thus, while variants of AcrIIA5 capable of effectively interfering with Cas9 DNA binding are likely within the realm of evolutionary possibility, the full development of this inhibitory property does not appear to have been naturally favored. This implies that inhibition of Cas9 DNA binding is likely to be an optimization challenge specific to each Cas9 ortholog. Conversely, inhibition of Cas9 catalytic activity is achievable through mechanisms independent of the Cas9 ortholog, making it a preferred target for broad-spectrum Acrs. This interpretation also aligns with our observation that AcrIIA5 mutants with enhanced potency in the *E. coli* CRISPRi assay assessing Cas9 DNA binding showed a strong, but not enhanced activity compared to wild-type AcrIIA5 in the human cell experiment based on editing the CCR5 locus with catalytically competent Cas9 (Supplementary Figure S10).

Our CRISPRi DMS screen further revealed that the β3-β4 mobile loop of AcrIIA5 exhibits a high tolerance to single point mutations. This finding is consistent with phage plaque experiments by Garcia et al. (22), where a triple mutation (His66, Asn70, His73) within the same loop of AcrIIA5 was well-tolerated and did not impair *Spy*Cas9 inhibition in this *in vivo* experimental setup. In contrast, using *in vitro* cleavage assays, An et al. (34) observed that the same triple mutant failed to inhibit Cas9, highlighting a potential mechanistic gap that may explain why AcrIIA5 can inhibit *Spy*Cas9 DNA binding *in vivo* but not *in vitro*. In addition, Garcia et al. found that this triple mutant failed to inhibit Cas9 orthologs other than *Spy*Cas9, suggesting that the role of the β3-β4 loop may vary depending on both the experimental context and the specific Cas9 ortholog. Notably, the mutants showing the strongest enhancement over wild-type AcrIIA5 in our *E. coli* CRISPRi validation experiments (K58M, D69A and E76L in Figure 6D) are located directly within or close to the β3-β4 mobile loop in AcrIIA5 (Figure 6A and Supplementary Figure S3B). This suggests that this region may be a particularly interesting target for further engineering.

Moreover, a number of sites distributed throughout the structure of AcrIIA5 were found to be critical for its ability to inhibit Cas9 DNA binding *in vivo* (Figure 6C). These are predominantly neutral, non-polar and therefore hydrophobic residues. Given that AcrIIA5 is a broad-spectrum inhibitor compatible with a variety of structurally divergent type II-A and -C Cas9 orthologs, these mutation-sensitive residues likely are critical for stabilizing the AcrIIA5 fold rather than representing individual Acr-Cas interactions essential for inhibition. Interestingly, the N-terminal, disordered region in AcrIIA5 (first ∼20 residues) did not show a particularly high sensitivity to point mutations compared to the remainder of the protein. This suggests that in addition to its structural plasticity, a property hypothesized to facilitate binding to structurally diverse Cas9-sgRNA complexes (34), the N-terminal disordered region possesses some variability with respect to its actual sequence. This observation leads us to propose a two-step interaction mechanism: first, the N-terminal region of AcrIIA5 would act as a weak anchor to the Cas9-sgRNA complex. Subsequently, the rest of the AcrIIA5 structure would bind and interfere with the Cas9-sgRNA complex, facilitated by the proximity established by the initial tethering. This unique property could explain the high efficiency of AcrIIA5 across a wide range of Cas9 orthologs.

In the future, it will be possible to extend our DMS pipeline to other CRISPR-Cas orthologs and Acr types and families. We here used a CRISPRi-based gene circuit as our readout because it was easily implemented with *Spy*Cas9 and provided a decent dynamic range under our experimental conditions. However, it should also be feasible to develop selection circuits based on DNA or RNA target cleavage or even Cas effector binding. Furthermore, while we have prioritized comprehensive coverage of single point mutants in this study, it will be interesting to extend our pipeline to insertions and deletions as well as higher order mutants to resolve additive or epistatic effects.

Taken together, our study establishes Acr mutational fitness landscapes created via DMS as a powerful resource to explore the Acr evolutionary design space and inform the engineering of Acrs that are optimized for selected applications in CRISPR genome engineering.

## Supplementary Material

gkae1052_Supplemental_Files

## Data Availability

Plasmid sequences (Genbank files) are provided as Supplementary data 1. NGS read distributions for AcrIIA4 and -5 are available as Supplementary data 2–4. Predicted flow cytometry plots for each single AcrIIA4 and -5 mutant are available as Supplementary data 5 and 6, respectively. Code is available on GitHub (https://github.com/mjendrusch/acr-dms), the entire datasets are accessible on Zenodo (https://dx.doi.org/10.5281/zenodo.5221560), and the DMS NGS data is available on GEO (https://www.ncbi.nlm.nih.gov/geo/query/acc.cgi?acc=GSE254101). Data will also be made available from the corresponding author upon reasonable request.
