# Supplementary material for: A deep mutational scanning platform to characterize the fitness landscape of anti-CRISPR proteins
Source: Nucleic Acids Res. 2024 Nov 18;52(22):e103. doi: 10.1093/nar/gkae1052 (PMC11662660; doi:10.1093/nar/gkae1052)
Supplement: gkae1052_Supplemental_Files [file gkae1052_supplemental_files.zip › Supplementary_Information_revision3.pdf]

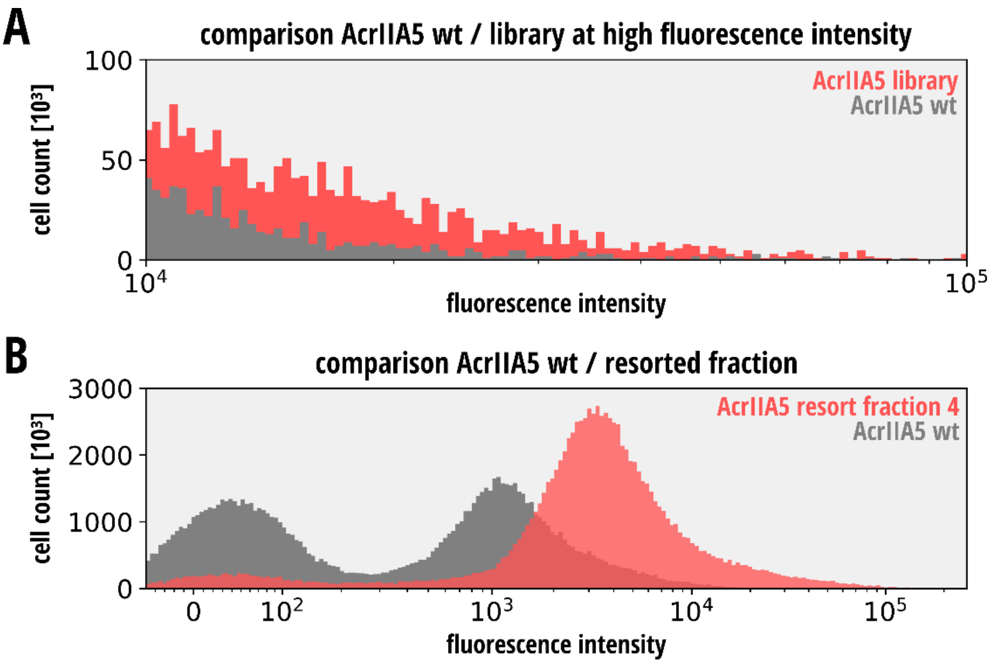

Supplementary Figure S1: The AcrIIA5 library contains variants that are more potent than AcrIIA5 wild-type. *E. coli* carrying plasmids expressing an RFP reporter, d*Spy*Cas9, and an RFP gene-targeting sgRNA were transformed with either a plasmid encoding the wild-type AcrIIA5 or the AcrIIA5 mutant library covering all possible single point mutations, followed flow cytometry analysis. (**A**) Overlay histogram comparing the AcrIIA5 wt to the AcrIIA5 library prior sorting/enrichment (see data in Figure 2), focusing on the high fluorescence fraction (fluorescence intensity > 10^4 a.u). (**B**) Resort of the AcrIIA5 library fraction corresponding to highly active variants (see fraction 4 in Figure 3D), and comparison to AcrIIA5 wild-type (wt).

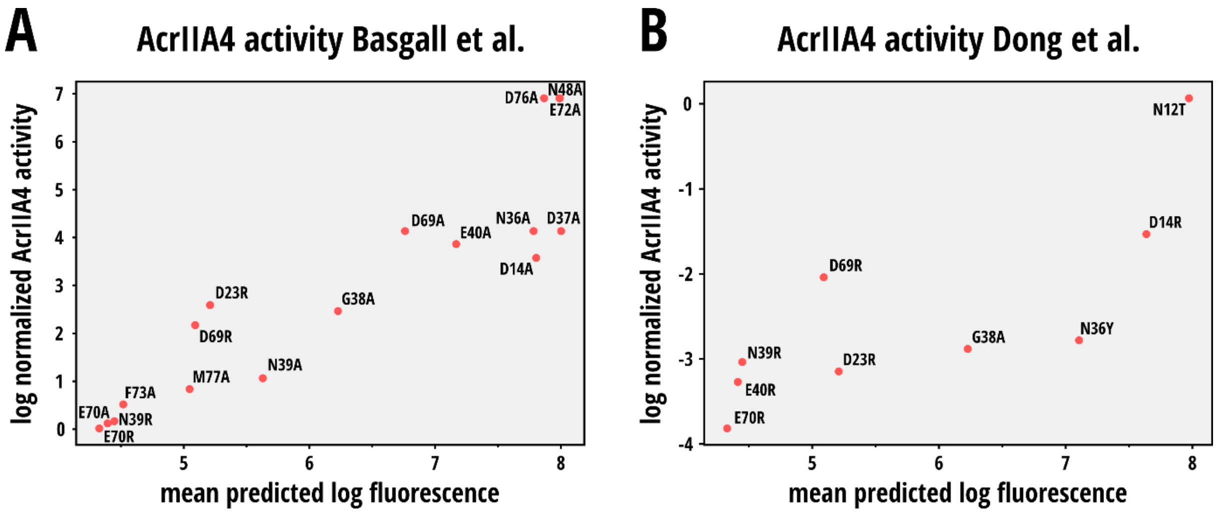

Supplementary Figure S2: Cross-validation of the AcrIIA4 DMS data sets with previously reported measurements of AcrIIA4 mutant activity. Scatter plots of the predicted mean log fluorescence derived from our DMS analysis and the corresponding Acr mutant activity. "AcrIIA4 activity" corresponds to gene drive inhibition (Basgall et al, 2018) in **A,** and to the relative *in-vitro* DNA cleavage inhibition (Dong et al, 2017) in **B**.

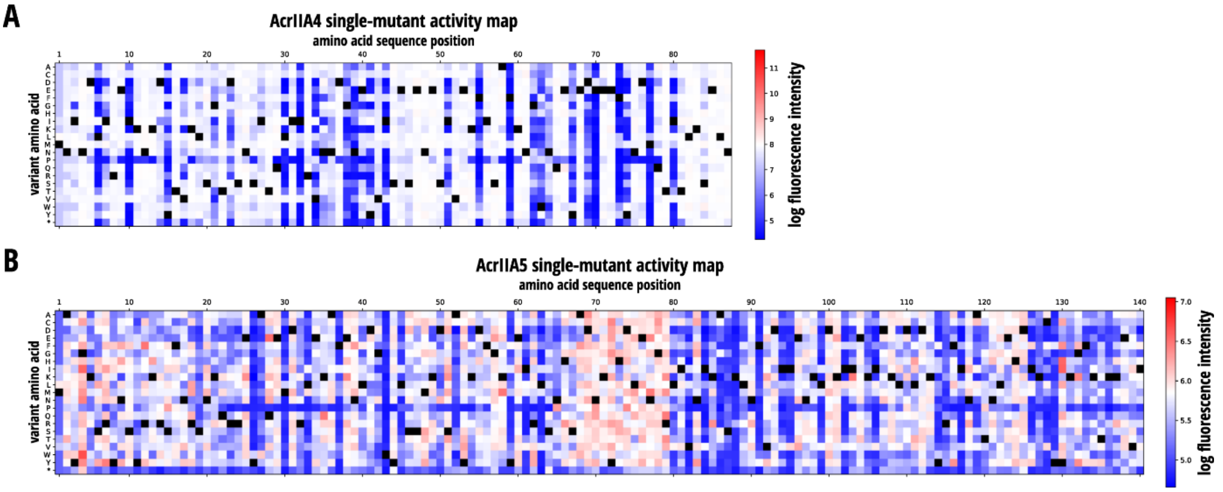

Supplementary Figure S3: Mean log fluorescence for each single variant. Reads from all fractions were summed up for each single AcrIIA4 (A) or AcrIIA5 (B) mutant. Heat maps indicate the relative fraction of these sums from all reads and display their mean from three biological replicates. Black squares indicate the wild-type amino acids.

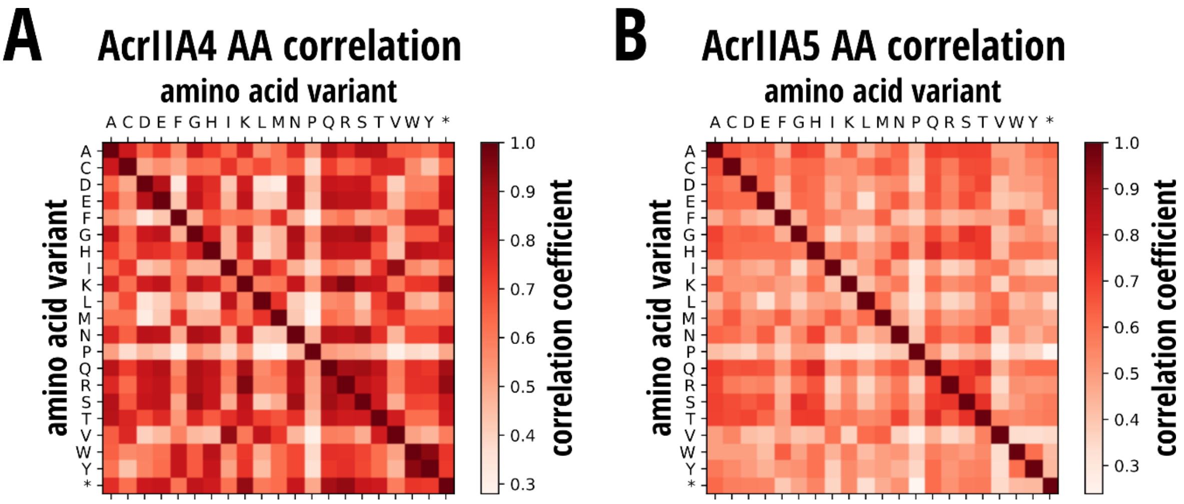

Supplementary Figure S4: Heat maps indicating the Pearson's correlation coefficient of the functional impact between the indicated amino acid exchanges for AcrIIA4 (A) and AcrIIA5 (B).

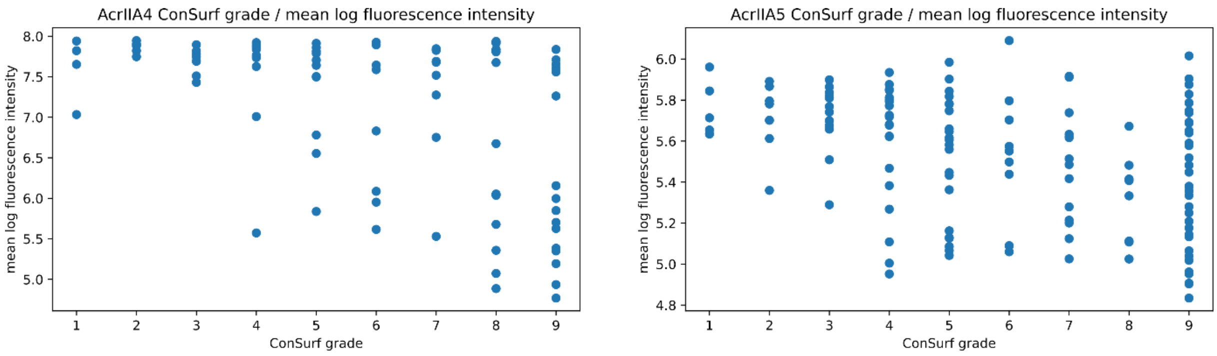

Supplementary Figure S5: Acr mutant activity is weakly correlated with protein sequence conservation. Scatter plot showing the Acr mutant activity (as mean log fluorescence intensity) in relation to the ConSurf grade, a measure for sequence conservation, for AcrIIA4 (left) and AcrIIA5 (right).

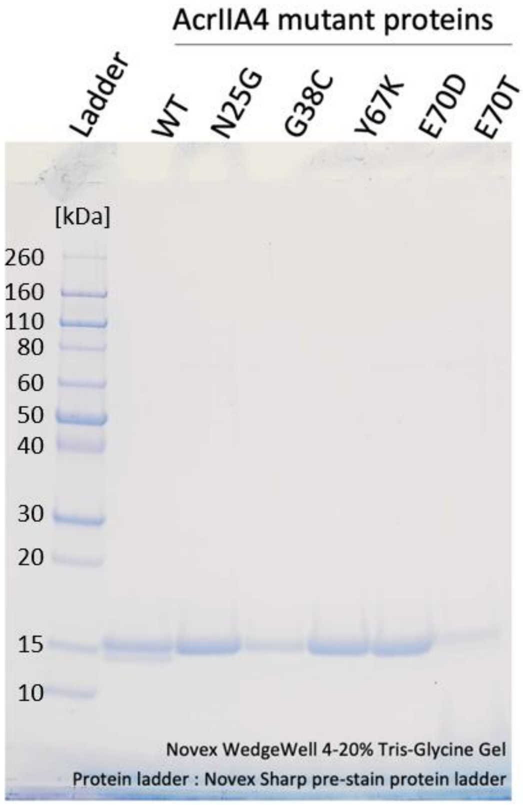

Supplementary Figure S6: Coomassie-stained SDS page gel of purified AcrIIA4 mutants. Note that the AcrIIA4 variants are tagged with an N-terminal His-tag and a TEV cleavage site, which increases their molecular weight to 12.3 kDa.

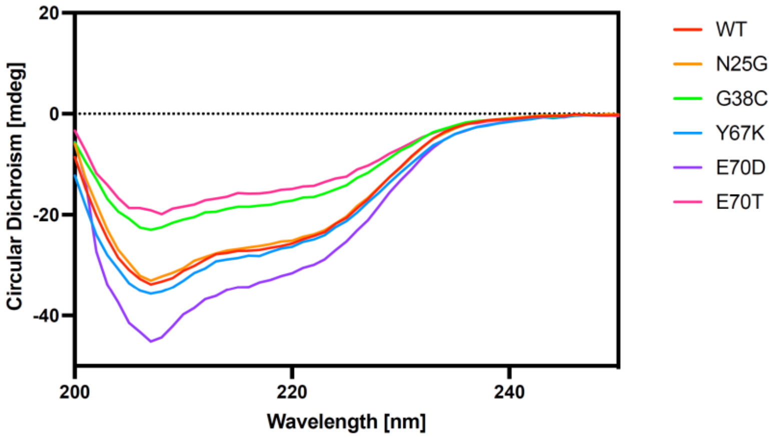

Supplementary Figure S7: CD spectra for AcrIIA4 wild-type (WT) as well as mutant proteins. Spectra show a minimum at around 207 nm, typical of mixed alpha and beta secondary structures.

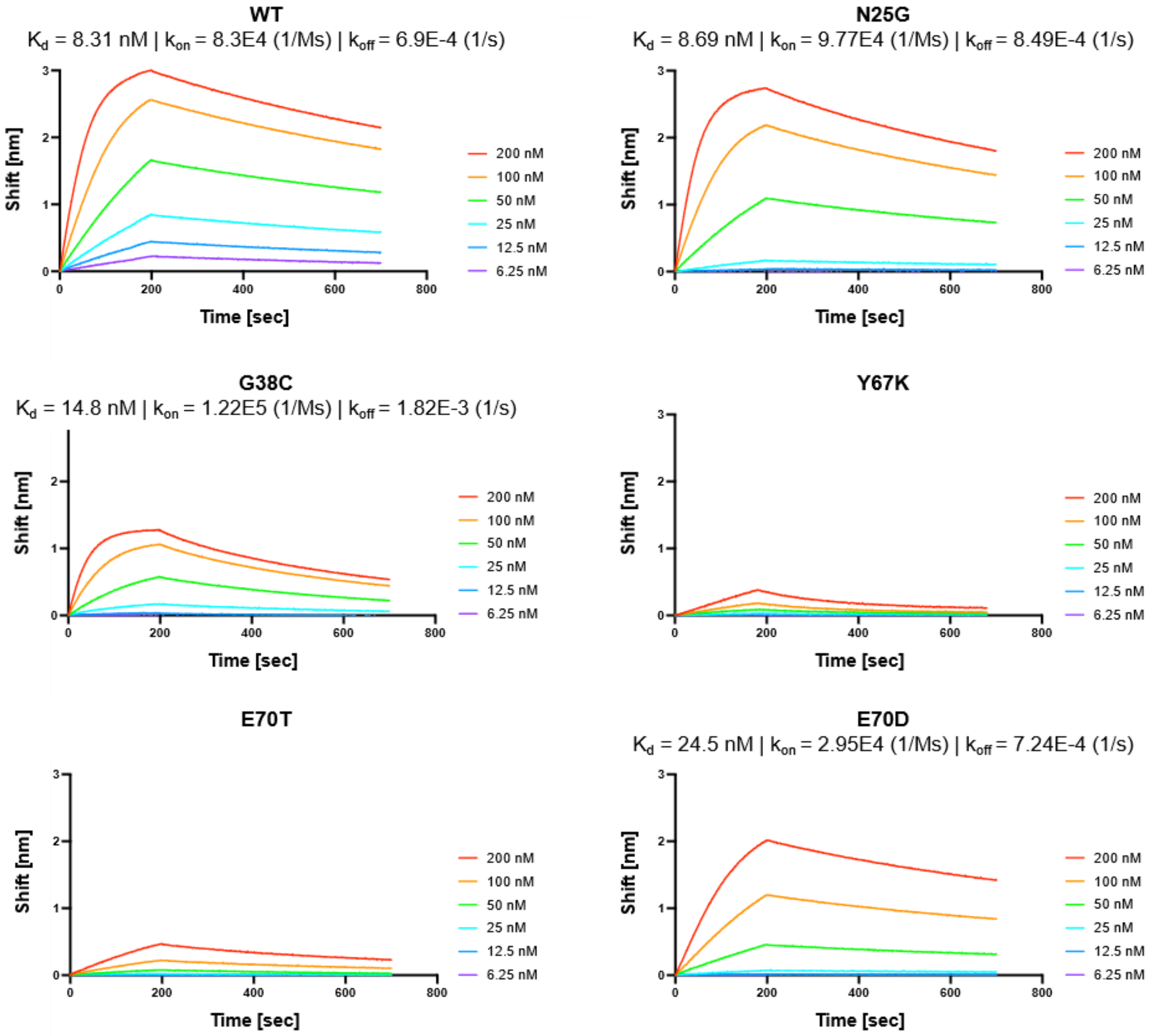

Supplementary Figure S8: Measurements of Cas9 binding affinity for AcrIIA4 variants. Biolayer interferometry binding data from Gator probes at different Acr protein concentrations. For the Y67K and E70T mutants, the K_d, k_{on} and k_{off} values are not provided due to very low binding. wt, wild-type.

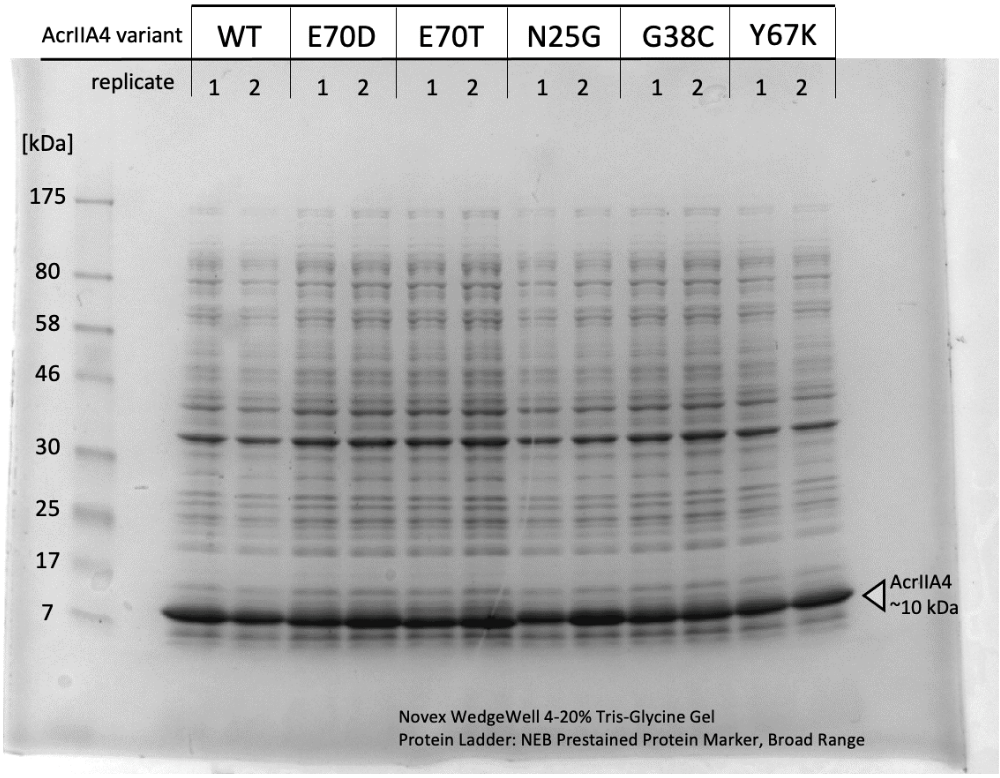

Supplementary Figure S9: **Acr expression analysis.** Different AcrIIA4 variants were expressed in the DH5a expression strain using the pBAD24 vector. Cell lysates were then analyzed on a Coomassie-stained SDS-PAGE gel. The grey arrow indicates bands that represent AcrIIA4 variants, i.e. wild-type (WT) or the indicated mutants.

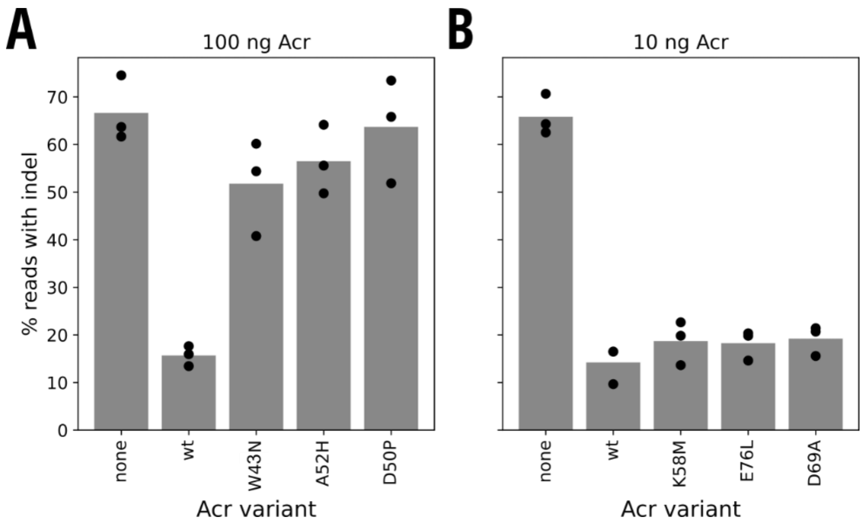

Supplementary Figure S10: AcrIIA5 mutants that impair CRISPRi in *E. coli* also inhibit genome editing in mammalian cells. HEK293T cells were co-transfected with (i) a construct co-expressing *Spy*Cas9 and a sgRNA targeting the CCR5 locus and (ii) a construct encoding the indicated AcrIIA5 mutant (or wild-type as control). A high (100 ng; panel **A**) or low (10 ng; panel **B**) Acr construct dose was used during transfection per well to facilitate discriminating between Acr mutants that, in the CRISPRi screen, showed a weak or strong activity, respectively. Post transfection, cells were incubated for 72 h, followed by NGS to assess InDel frequencies at the target locus. Wt, wild-type AcrIIA5. Bars represent means, dots indicate individual data points from n = 3 independent experiments.

Supplementary Table T1: List of plasmids used in this study

Amp = Ampicillin resistance, Kan = Kanamycin resistance, Cm = Chloramphenicol resistance, Ori = Origin of replication, ColE1 = high copy origin of replication (high copy), pSC101 = low copy origin of replication, p15A = low copy origin of replication, sfGFP = superfolder GFP, M0052 = M0052 degradation tag, CMV = cytomegalovirus, BGH = bovine growth hormone

Plasmid ID	Name	Resistance	Ori	Description
1	pJUMP27-1A(sfGFP)	Kan	pSC101	Addgene #126974, backbone for RFP expression plasmid
2	pBbA5c-RFP	Cm	P15A	Addgene #35281, backbone for d*Spy*Cas9 expression plasmid
3	pBAD24-sfGFPx1	Amp	ColE1	Addgene #51558, backbone for Acr expression plasmid
4	pBAD24_AcrIIA4	Amp	ColE1	Arabinose inducible expression of AcrIIA4, template plasmid for AcrIIA4 library generation
5	pBAD24_AcrIIA5(phage D4276)	Amp	ColE1	Arabinose inducible expression of AcrIIA5, template plasmid for AcrIIA4 library generation
6	pJUMP27_J23102_mRFP _M0052	Kan	pSC101	Constitutive RFP reporter expression with M0052 degradation tag
7	pBbA5C_sgMCS	Cm	P15A	Empty vector with additional MCS for sgRNA cassette
8	pBbA5C_sgMCS_dSpyCa s9	Cm	P15A	IPTG inducible expression of d*Spy*Cas9, additional MCS for sgRNA cassette
9	pBbA5C_sgMCS_dSpyCa s9 _RFPguide	Cm	P15A	IPTG inducible expression of d*Spy*Cas9, sgRNA cassette with spacer targeting RFP
10	pBAD24_AcrIIA4_V17D	Amp	ColE1	AcrIIA4 single mutant, used for model training
11	pBAD24_AcrIIA4_K18M	Amp	ColE1	AcrIIA4 single mutant, used for model training
12	pBAD24_AcrIIA4_G21W	Amp	ColE1	AcrIIA4 single mutant, used for model training
13	pBAD24_AcrIIA4_D23R	Amp	ColE1	AcrIIA4 single mutant, used for model training

14	pBAD24_AcrIIA4_S24P	Amp	ColE1	AcrIIA4 single mutant, used for model training
15	pBAD24_AcrIIA4_N25G	Amp	ColE1	AcrIIA4 single mutant, used for model training
16	pBAD24_AcrIIA4_N25Y	Amp	ColE1	AcrIIA4 single mutant, used for model training
17	pBAD24_AcrIIA4_G38C	Amp	ColE1	AcrIIA4 single mutant, used for model training
18	pBAD24_AcrIIA4_G38V	Amp	ColE1	AcrIIA4 single mutant, used for model training
19	pBAD24_AcrIIA4_E40I	Amp	ColE1	AcrIIA4 single mutant, used for model training
20	pBAD24_AcrIIA4_N48A	Amp	ColE1	AcrIIA4 single mutant, used for model training
21	pBAD24_AcrIIA4_Y67K	Amp	ColE1	AcrIIA4 single mutant, used for model training
22	pBAD24_AcrIIA4_E70D	Amp	ColE1	AcrIIA4 single mutant, used for model training
23	pBAD24_AcrIIA4_E70R	Amp	ColE1	AcrIIA4 single mutant, used for model training
24	pBAD24_AcrIIA4_E70T	Amp	ColE1	AcrIIA4 single mutant, used for model training
25	pBAD24_AcrIIA4_M77A	Amp	ColE1	AcrIIA4 single mutant, used for model training
26	pBAD24_AcrIIA5_G4P	Amp	ColE1	AcrIIA5 single mutant, used for model training
27	pBAD24_AcrIIA5_S6Q	Amp	ColE1	AcrIIA5 single mutant, used for model training
28	pBAD24_AcrIIA5_Q28Y	Amp	ColE1	AcrIIA5 single mutant, used for model training
29	pBAD24_AcrIIA5_M48E	Amp	ColE1	AcrIIA5 single mutant, used for model training
30	pBAD24_AcrIIA5_D50P	Amp	ColE1	AcrIIA5 single mutant, used for model training
31	pBAD24_AcrIIA5_S51W	Amp	ColE1	AcrIIA5 single mutant, used for model training
32	pBAD24_AcrIIA5_A52M	Amp	ColE1	AcrIIA5 single mutant, used for model training
33	pBAD24_AcrIIA5_K54F	Amp	ColE1	AcrIIA5 single mutant, used for model training
34	pBAD24_AcrIIA5_S67V	Amp	ColE1	AcrIIA5 single mutant, used for model training
35	pBAD24_AcrIIA5_N70K	Amp	ColE1	AcrIIA5 single mutant, used for model training

36	pBAD24_AcrIIA5_N70L	Amp	ColE1	AcrIIA5 single mutant, used for model training
37	pBAD24_AcrIIA5_L75T	Amp	ColE1	AcrIIA5 single mutant, used for model training
38	pBAD24_AcrIIA5_G78N	Amp	ColE1	AcrIIA5 single mutant, used for model training
39	pBAD24_AcrIIA5_E138R	Amp	ColE1	AcrIIA5 single mutant, used for model training
40	pBAD24_AcrIIA5_K71E	Amp	ColE1	AcrIIA5 single mutant, used for model training
41	pBAD24_AcrIIA5_I94H	Amp	ColE1	AcrIIA5 single mutant, used for model training
42	pBAD24_AcrIIA4_D36* (TAA stop)	Amp	ColE1	AcrIIA4 with stop codon, used for circuit evaluation
43	pBAD24_AcrIIA4_D36* (TAG stop)	Amp	ColE1	AcrIIA4 with stop codon, used for circuit evaluation
44	pBAD24_AcrIIA4_D36* (TGA stop)	Amp	ColE1	AcrIIA4 with stop codon, used for circuit evaluation

Plasmids for cell culture assays

**Plasmid ID**	**Name**	**Description**
45	ssAAV-SV40 hRluc-TK hLuc- H1-gRNA hLuc	Dual luciferase plasmid, containing a sgRNA expression cassette to target the hLuc gene
46	3xFlag-NLS-SpCas9-NLS	spyCas9 expression plasmid
47	pCMV_AcrIIA4	CMV promoter, AcrIIA4 wild-type, BGH polyA
48	pCMV_AcrIIA5	CMV promoter, AcrIIA5 wild-type, BGH polyA
49	pCMV_AcrIIA4_K18M	CMV promoter, AcrIIA4 mutant K18M, BGH polyA
50	pCMV_AcrIIA4_G21Q	CMV promoter, AcrIIA4 mutant G21Q, BGH polyA
51	pCMV_AcrIIA4_S24K	CMV promoter, AcrIIA4 mutant S24K, BGH polyA
52	pCMV_AcrIIA4_S24P	CMV promoter, AcrIIA4 mutant S24P, BGH polyA
53	pCMV_AcrIIA4_N25G	CMV promoter, AcrIIA4 mutant N25G, BGH polyA
54	pCMV_AcrIIA4_I31Q	CMV promoter, AcrIIA4 mutant I31Q, BGH polyA
55	pCMV_AcrIIA4_E40I	CMV promoter, AcrIIA4 mutant E40I, BGH polyA
56	pCMV_AcrIIA4_E70T	CMV promoter, AcrIIA4 mutant E70T, BGH polyA
57	pCMV_AcrIIA4_M77A	CMV promoter, AcrIIA4 mutant M77A, BGH polyA
58	Cas_Acr (wt)	CMV promoter, Cas9 fused to wt AcrIIA4
59	Cas_Acr (L19E)	CMV promoter, Cas9 fused to AcrIIA4 mutant L19E
60	Cas_Acr (N35H)	CMV promoter, Cas9 fused to AcrIIA4 mutant N35H
61	Cas_Acr (E70N)	CMV promoter, Cas9 fused to AcrIIA4 mutant E70N
62	sgRNA_AAVS1	U6-driven AAVS1 locus targeting sgRNA
63	sgRNA_HEK	U6-driven HEK locus targeting sgRNA
64	sgRNA_CCR5	U6-driven CCR5 locus targeting sgRNA

65	pCMV_AcrIIA5	CMV promoter, AcrIIA5 wild-type, BGH polyA
66	pCMV_AcrIIA5	CMV promoter, AcrIIA5 mutant K58M, BGH polyA
67	pCMV_AcrIIA5	CMV promoter, AcrIIA5 mutant E76L, BGH polyA
68	pCMV_AcrIIA5	CMV promoter, AcrIIA5 mutant D59A, BGH polyA
69	pCMV_AcrIIA5	CMV promoter, AcrIIA5 mutant W43N, BGH polyA
70	pCMV_AcrIIA5	CMV promoter, AcrIIA5 mutant A52H, BGH polyA
71	pCMV_AcrIIA5	CMV promoter, AcrIIA5 mutant D50P, BGH polyA
72	CMV_Cas9/ U6_CCR5sgRNA	CMV promoter, *Spy*Cas9, BGH polyA, U6 promoter, CCR5 targeting sgRNA

Plasmids for protein purification

**Plasmid ID**	**Name**	**Description**
73	pET28a_AcrIIA4_wt	T7 Promoter, AcrIIA4 wild-type, T7 Terminator
74	pET28a_AcrIIA4_E70D	T7 Promoter, AcrIIA4 E70D mutant, T7 Terminator
75	pET28a_AcrIIA4_E70T	T7 Promoter, AcrIIA4 E70T mutant, T7 Terminator
76	pET28a_AcrIIA4_N25G	T7 Promoter, AcrIIA4 N25G mutant, T7 Terminator
77	pET28a_AcrIIA4_G38C	T7 Promoter, AcrIIA4 G38C mutant, T7 Terminator
78	pET28a_AcrIIA4_Y67K	T7 Promoter, AcrIIA4 Y67K mutant, T7 Terminator

Supplementary Table T2: sgRNA target sites. Sequences in 5'-3' direction, PAM sequences are bolded.

**sgRNA target sites**	**Description**
AACTTTCAGTTTAGCGGTCT**GGG**	RFP target site used for CRISPRi gene circuit in *E. coli* cells
GGTAGTCGGTCTTAGAGTCC**AGG**	Target site in hLuc used for cell culture validation (Figure 7B,C)
GGGAGGGAGAGCTTGGCAGG**GGG**	AAVS1 sgRNA, ON-target site
GGGAAGGGGAGCTTGGCAGG**TGG**	AAVS1 sgRNA, OFF-target site
GGCACTGCGGCTGGAGGTGG**GGG**	HEK sgRNA, ON-target site
TGCACTGCGGCCGGAGGAGG**TGG**	HEK sgRNA, OFF-target site
TGACATCAATTATTATACAT**CGG**	CCR5 sgRNA

Supplementary Table T3: AcrIIA4 and -5 libraries overview

**Library ID**	**Name**	**Description**	**Complexity (N x library)**
Library 1.1	pBAD24_AcrIIA4_mutant _library_1	Arabinose inducible expression of AcrIIA4 mutant library	342
Library 1.2	pBAD24_AcrIIA4_mutant _library_2	Arabinose inducible expression of AcrIIA4 mutant library	267
Library 1.3	pBAD24_AcrIIA4_mutant _library_3	Arabinose inducible expression of AcrIIA4 mutant library	287

Library 2.1	pBAD24_AcrIIA5_mutant _library_1	Arabinose inducible expression of AcrIIA5 mutant library	230
Library 2.2	pBAD24_AcrIIA5_mutant _library_2	Arabinose inducible expression of AcrIIA5 mutant library	212
Library 2.3	pBAD24_AcrIIA5_mutant _library_3	Arabinose inducible expression of AcrIIA5 mutant library	280

Supplementary Table T4: Bacterial strains generated in this study (excl. single mutants)

Strain ID Paper	**Plasmid 1** Resistance = Amp Ori = ColE1 Arabinose inducible Acr expression	**Plasmid 2** Resistance = Kan Ori = pSC101 Lac-inducible d*Spy*Cas9 expression	**Plasmid 3** Resistance = Cm Ori = P15A Constitutive RFP expression	**Description**
I	1 – pBAD24-1A(sfGFP)	8 - pBbA5C_sgMCS _dSpyCas9_RFPguide	6 - pJUMP27 _J23102_mRFP _M0052	Active CRISPRi system, no Acr expression (control)
II	4 - pBAD24 _AcrIIA4	8 - pBbA5C_sgMCS dSpyCas9_RFPguide	pJUMP27_J23102_ mRFP_M0052	Active CRISPRi system, suppressed by wt AcrIIA4 expression
III	5 - pBAD24 _AcrIIA5	8 - pBbA5C_sgMCS dSpyCas9_RFPguide	pJUMP27_J23102_ mRFP_M0052	Active CRISPRi system, suppressed by wt AcrIIA5 expression
IV.1	Library 1.1 – pBAD24_AcrIIA4_ mutant_library	8 - pBbA5C_sgMCS dSpyCas9_RFPguide	pJUMP27_J23102_ mRFP_M0052	Active CRISPRi system, partially suppressed by AcrIIA4 library expression
IV.2	Library 1.2 – pBAD24_AcrIIA4_ mutant_library	8 - pBbA5C_sgMCS dSpyCas9_RFPguide	pJUMP27_J23102_ mRFP_M0052	Active CRISPRi system, partially suppressed by AcrIIA4 library expression
IV.3	Library 1.3 – pBAD24_AcrIIA4_ mutant_library	8 - pBbA5C_sgMCS dSpyCas9_RFPguide	pJUMP27_J23102_ mRFP_M0052	Active CRISPRi system, partially suppressed by AcrIIA4 library expression
V.1	Library 2.1 – pBAD24_AcrIIA5_ mutant_library	8 - pBbA5C_sgMCS dSpyCas9_RFPguide	pJUMP27_J23102_ mRFP_M0052	Active CRISPRi system, partially suppressed by AcrIIA5 library expression

V.2	Library 2.2 – pBAD24_AcrIIA5_ mutant_library	8 - pBbA5C_sgMCS dSpyCas9_RFPguide	pJUMP27_J23102_ mRFP_M0052	Active CRISPRi system, partially suppressed by AcrIIA5 library expression
V.3	Library 2.3 – pBAD24_AcrIIA5_ mutant_library	8 - pBbA5C_sgMCS dSpyCas9_RFPguide	pJUMP27_J23102_ mRFP_M0052	Active CRISPRi system, partially suppressed by AcrIIA5 library expression
VI	4 - pBAD24 _AcrIIA4	-	-	pBAD-driven AcrIIA4 variants for expression strength comparison
