# Supplementary figures and images for: A deep mutational scanning platform to characterize the fitness landscape of anti-CRISPR proteins
Source: Nucleic Acids Res. 2024 Nov 18;52(22):e103. doi: 10.1093/nar/gkae1052 (PMC11662660; doi:10.1093/nar/gkae1052)

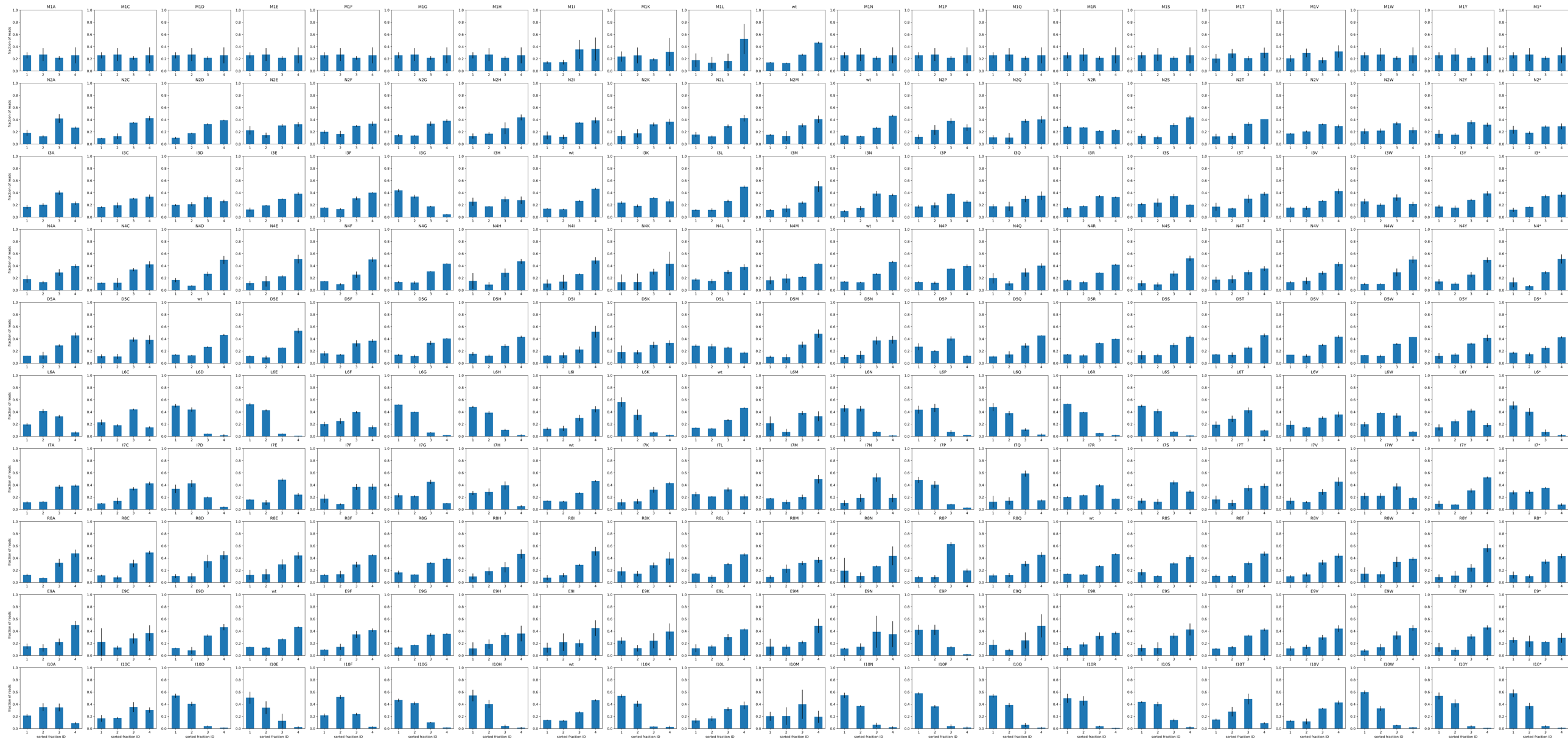

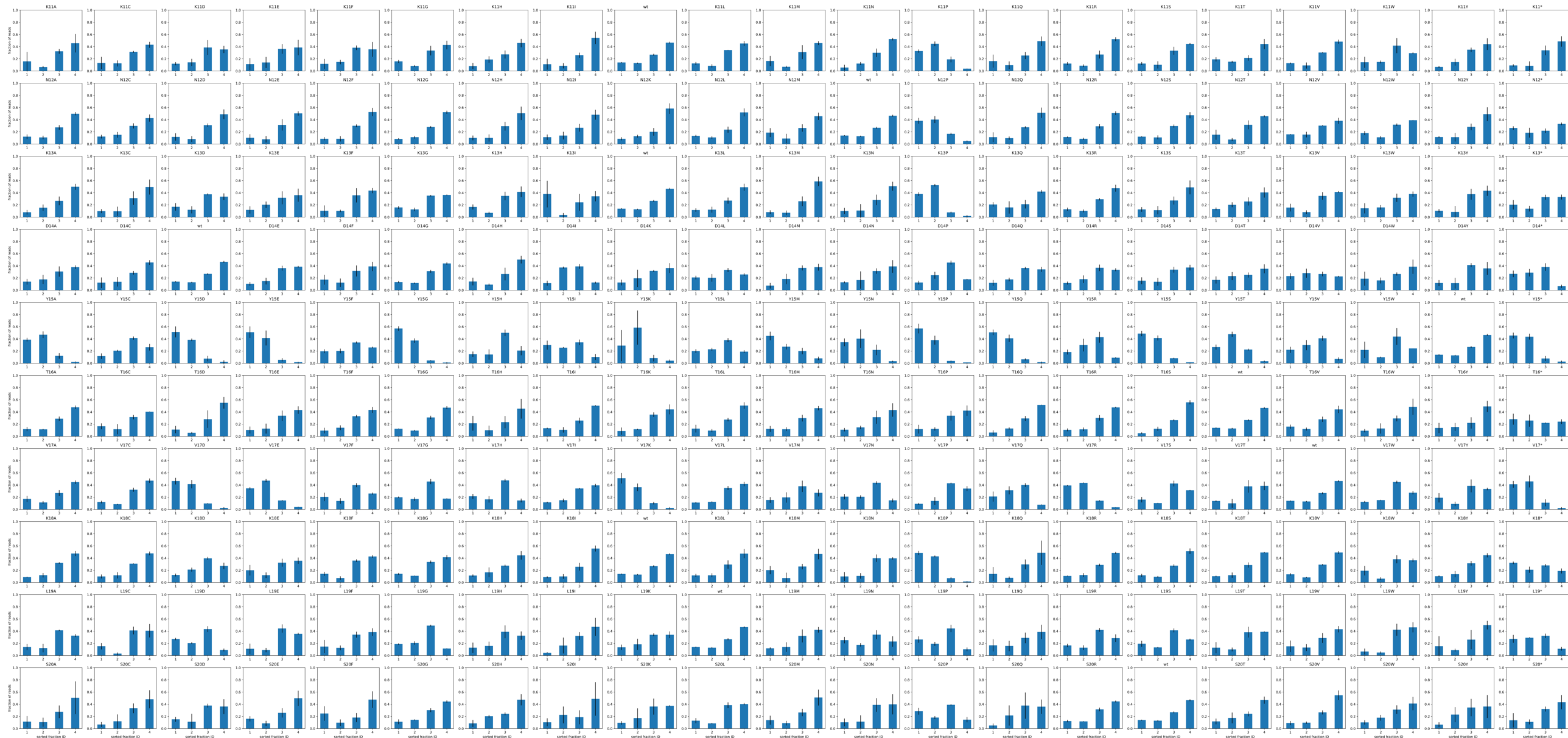

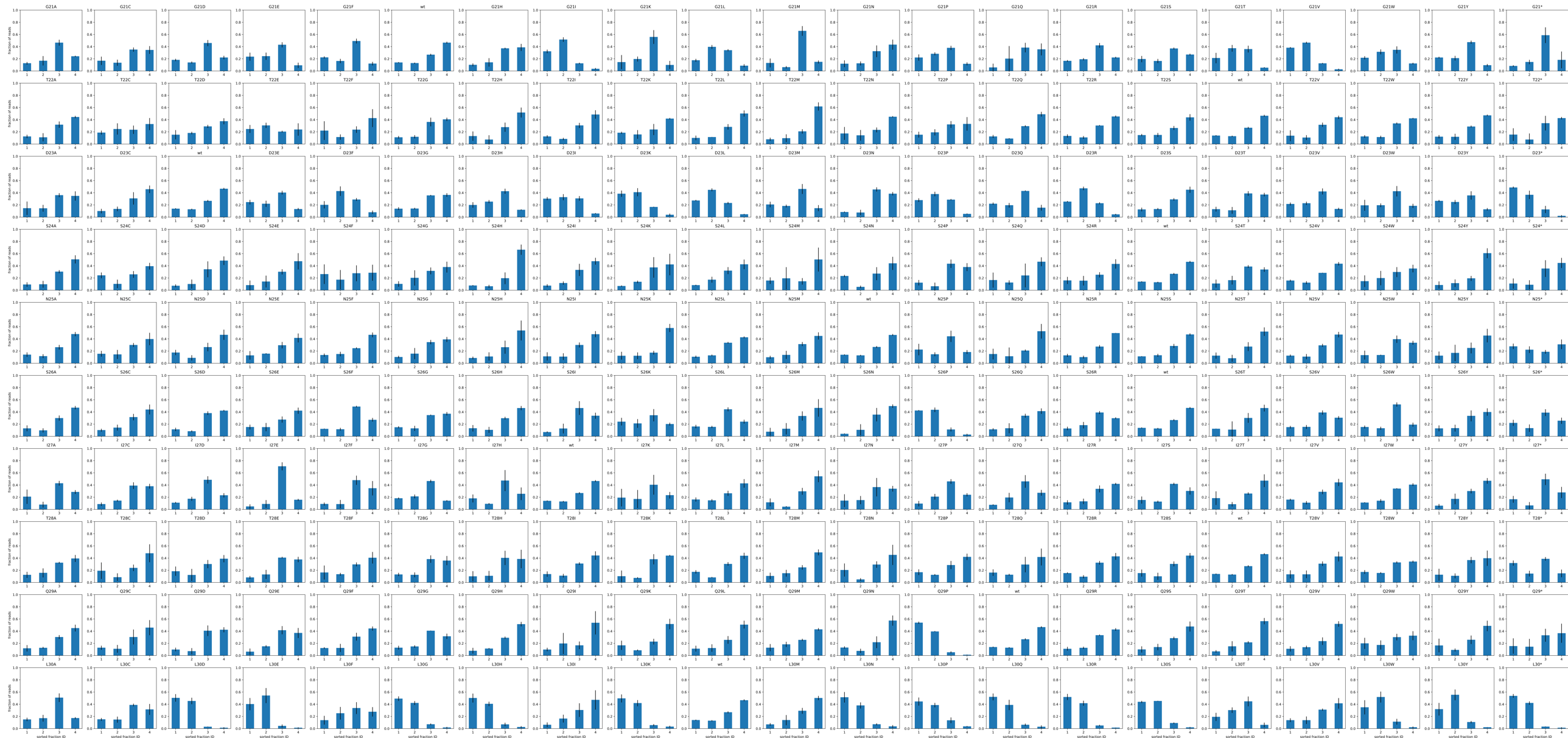

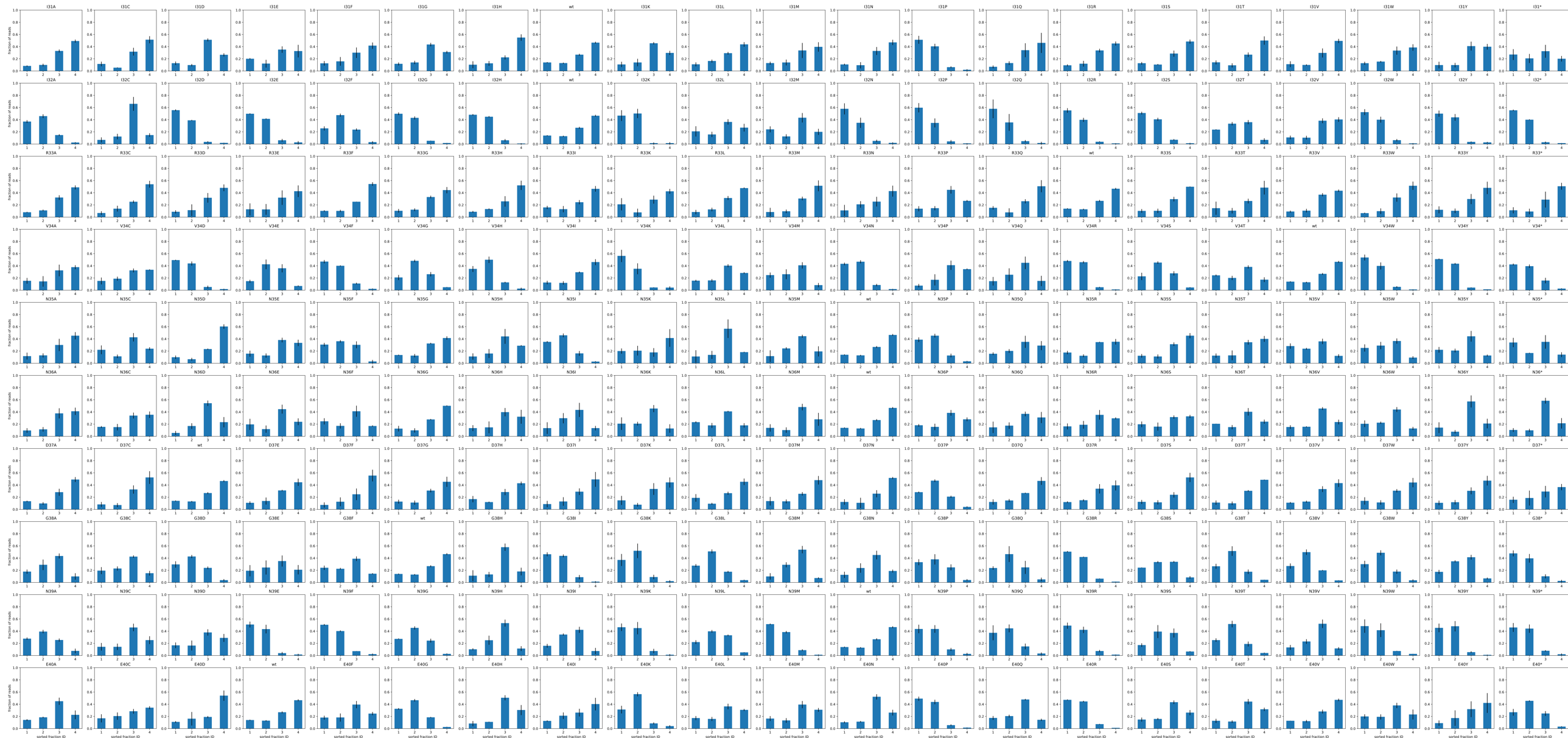

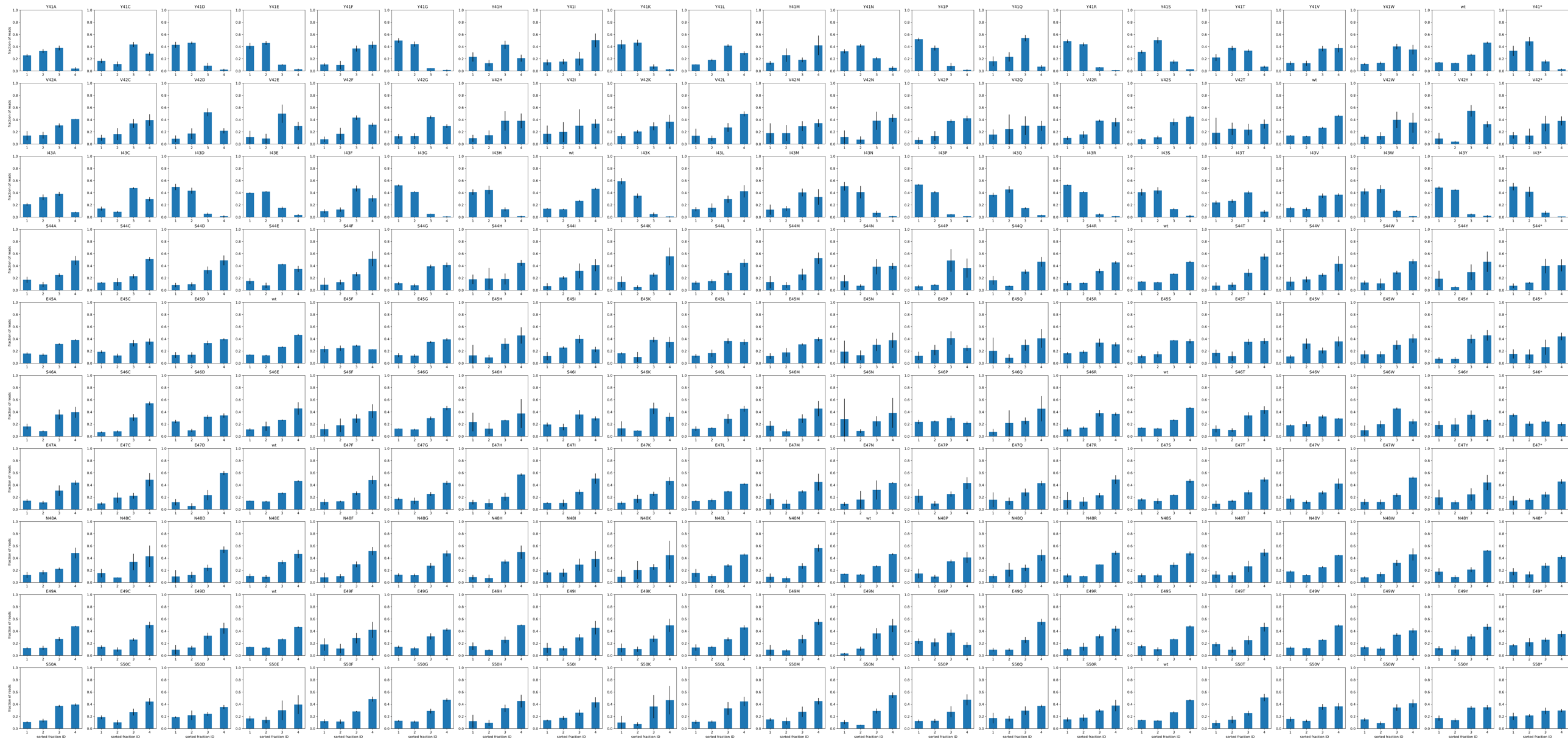

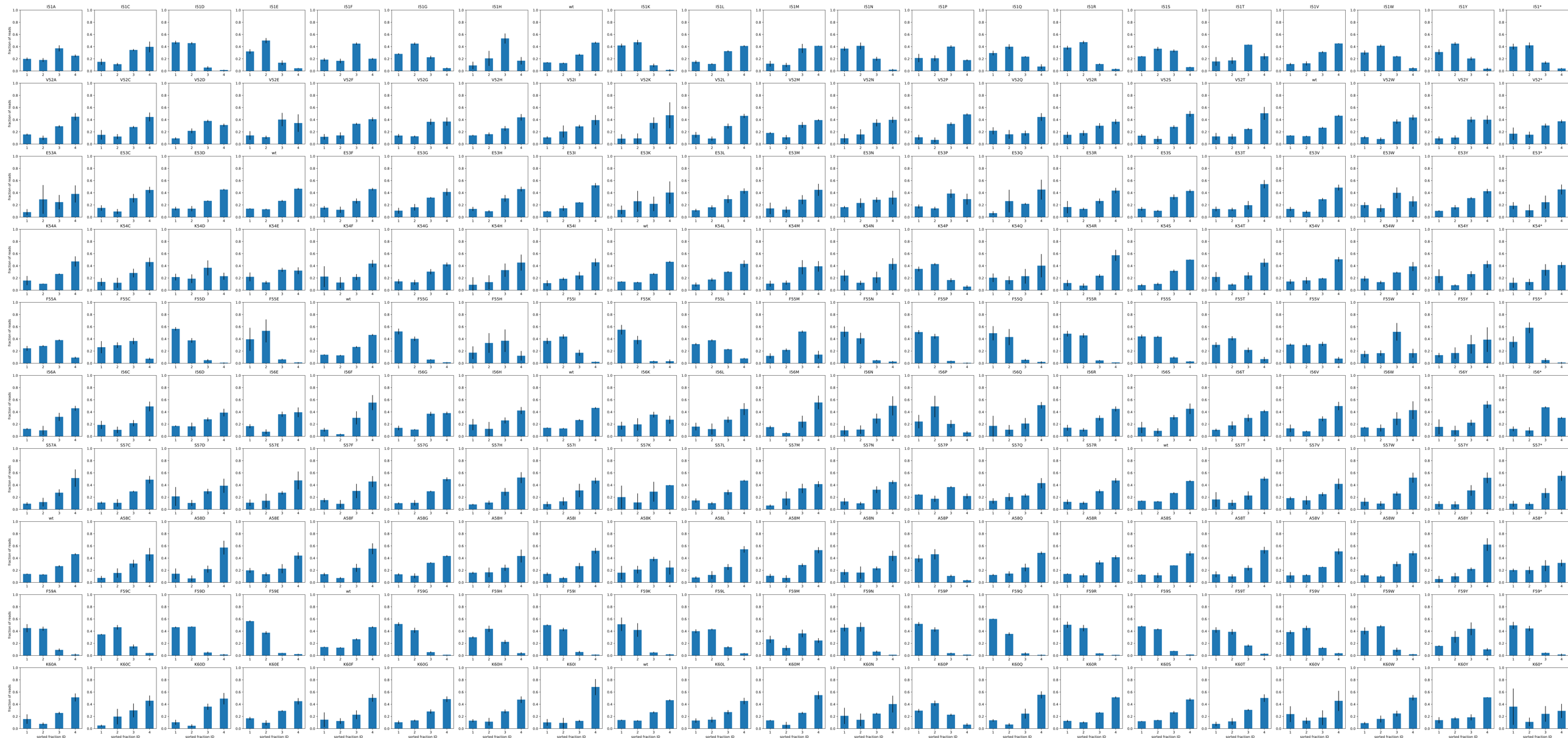

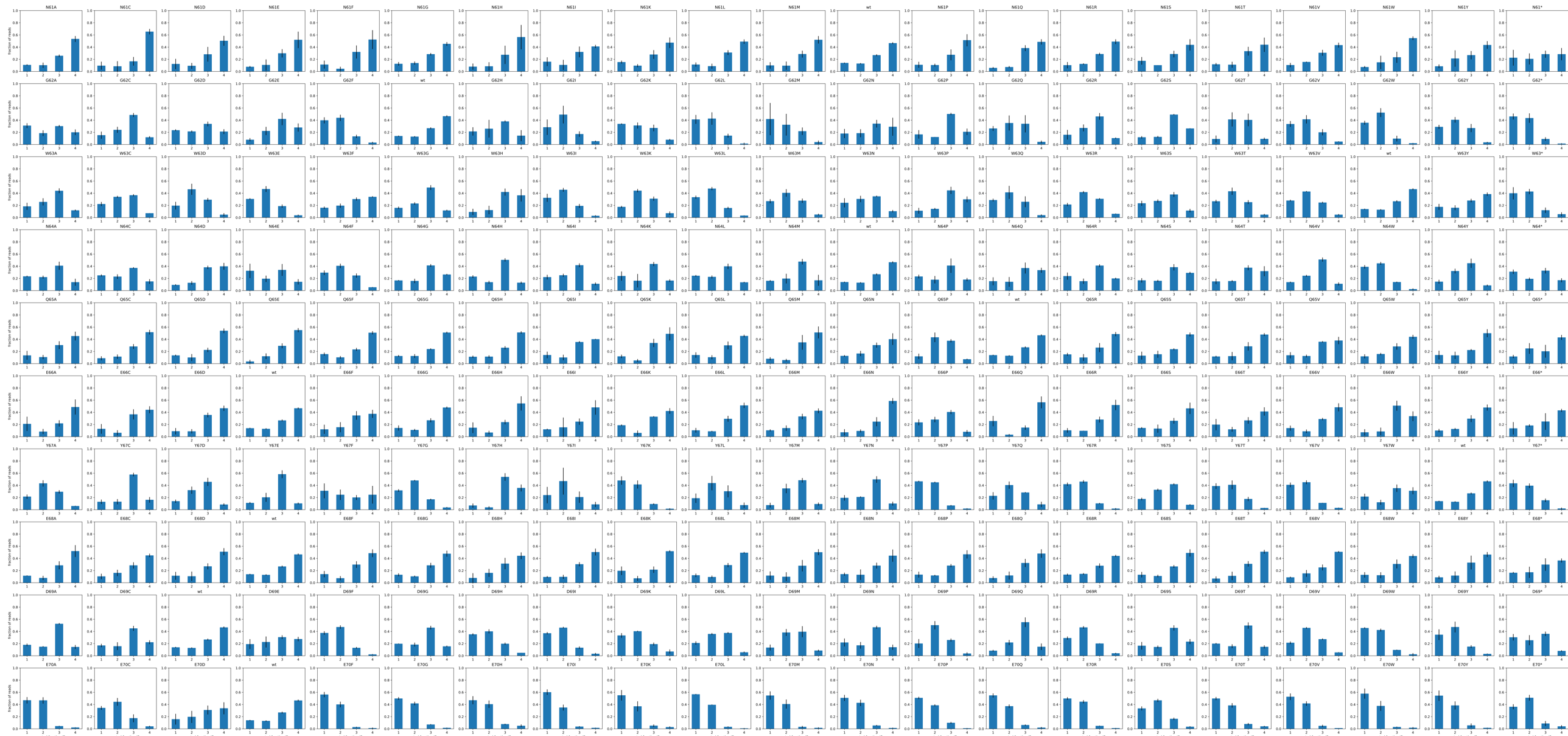

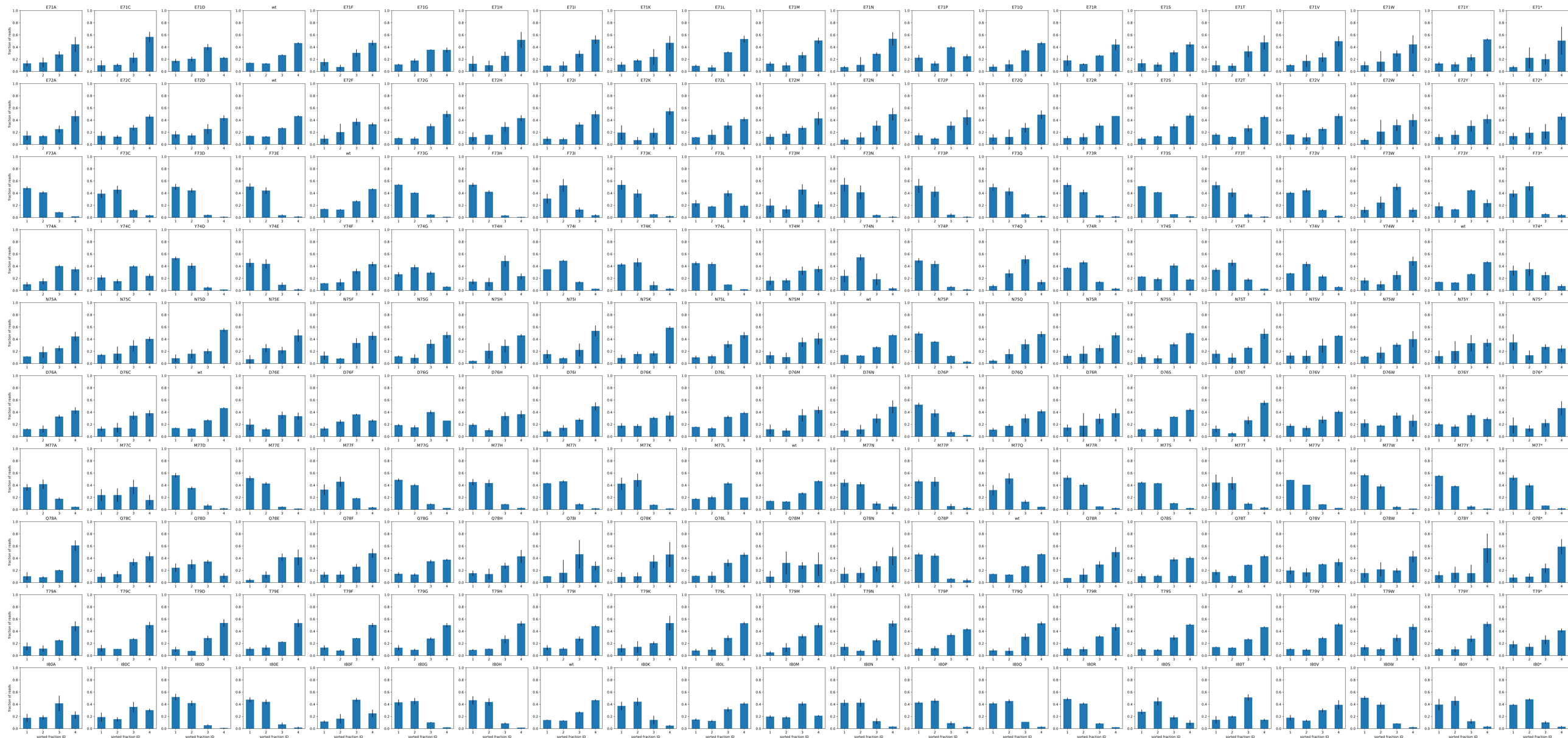

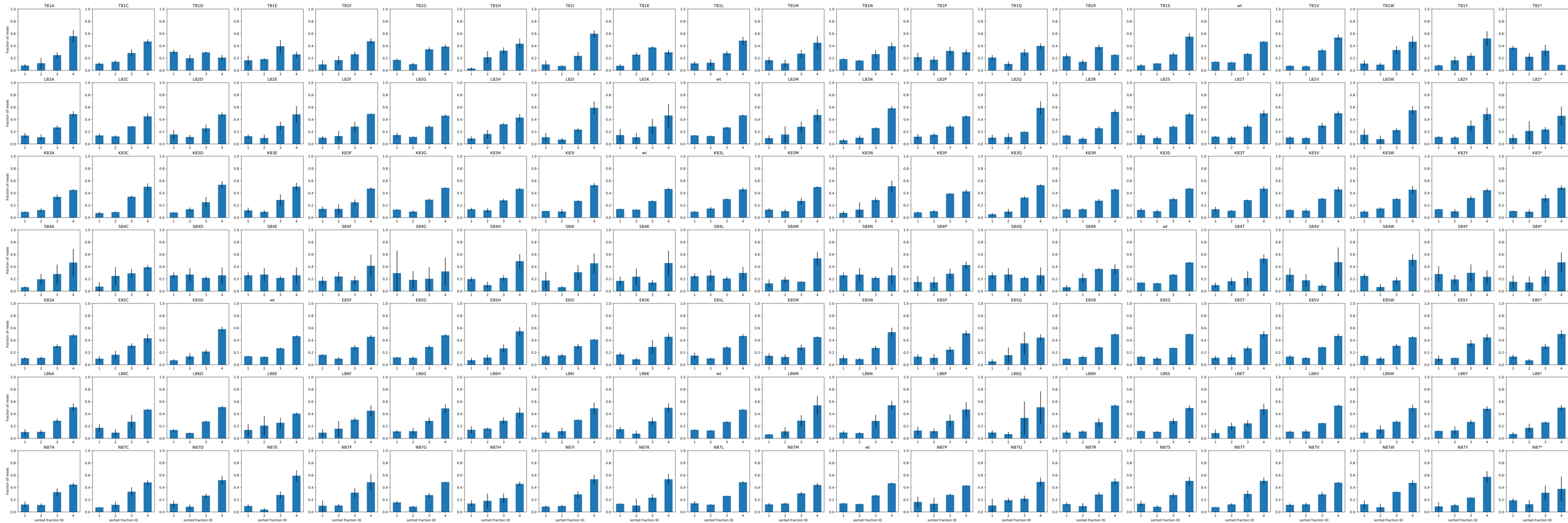

Supplement: gkae1052_Supplemental_Files [file gkae1052_supplemental_files.zip › Supplementary data 2.pdf]

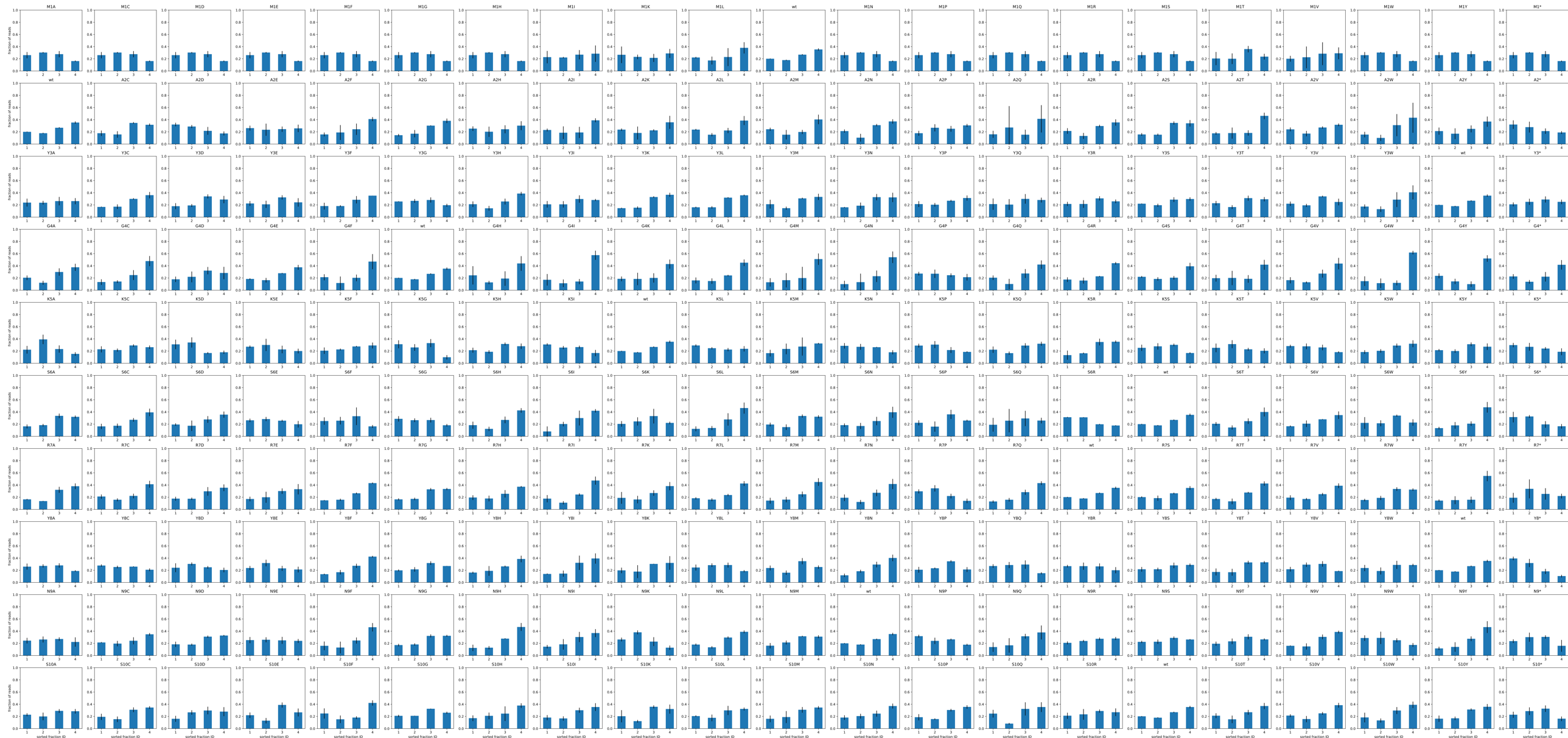

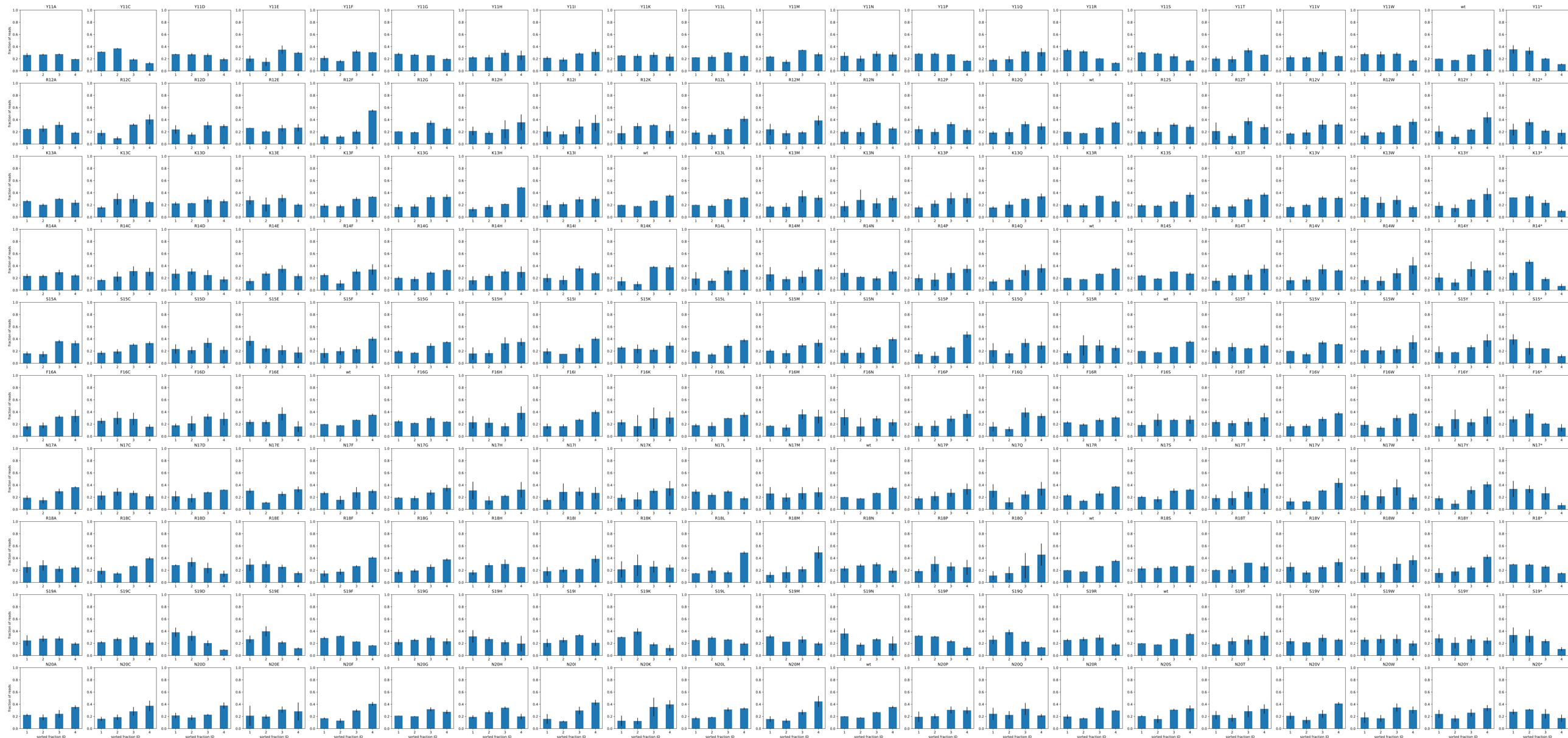

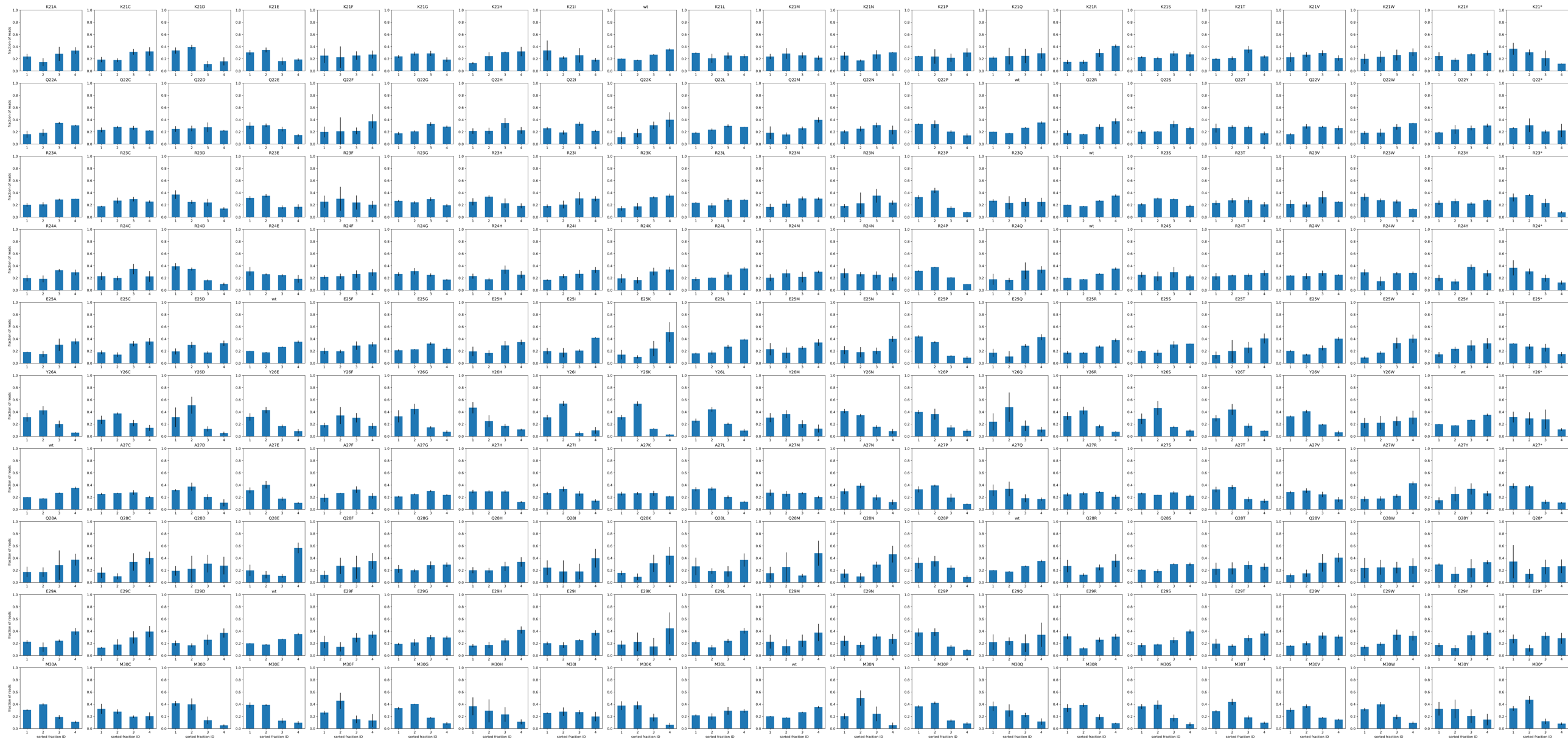

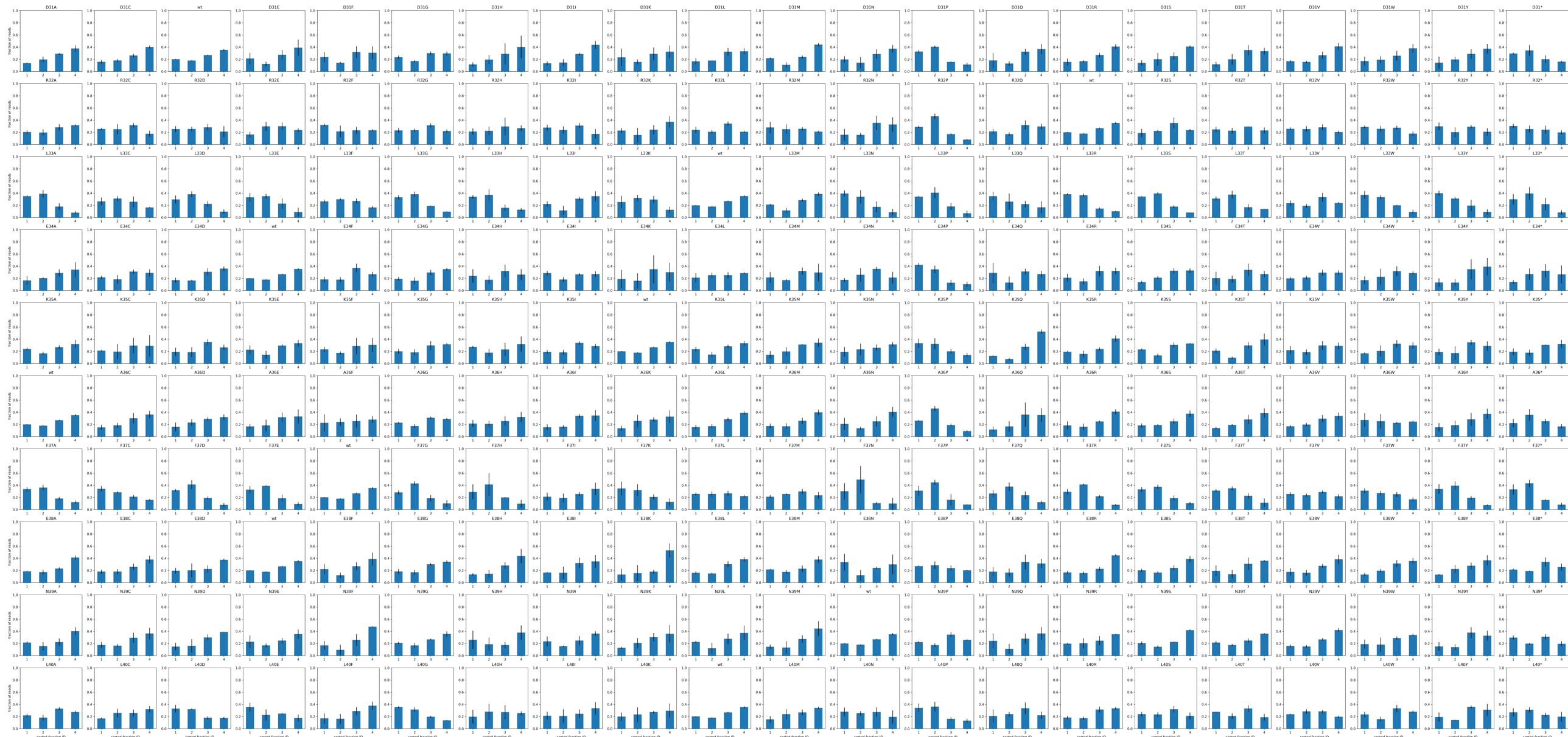

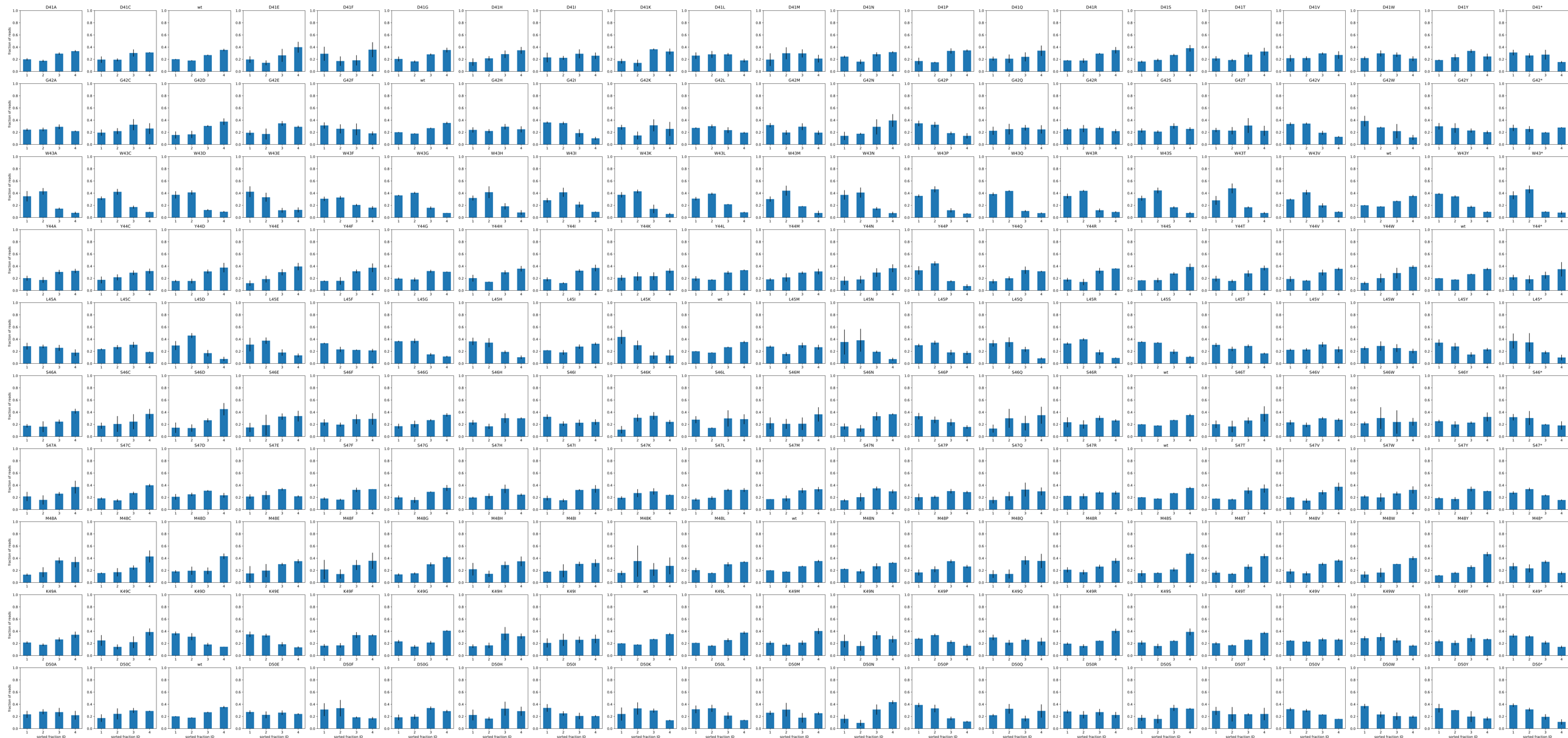

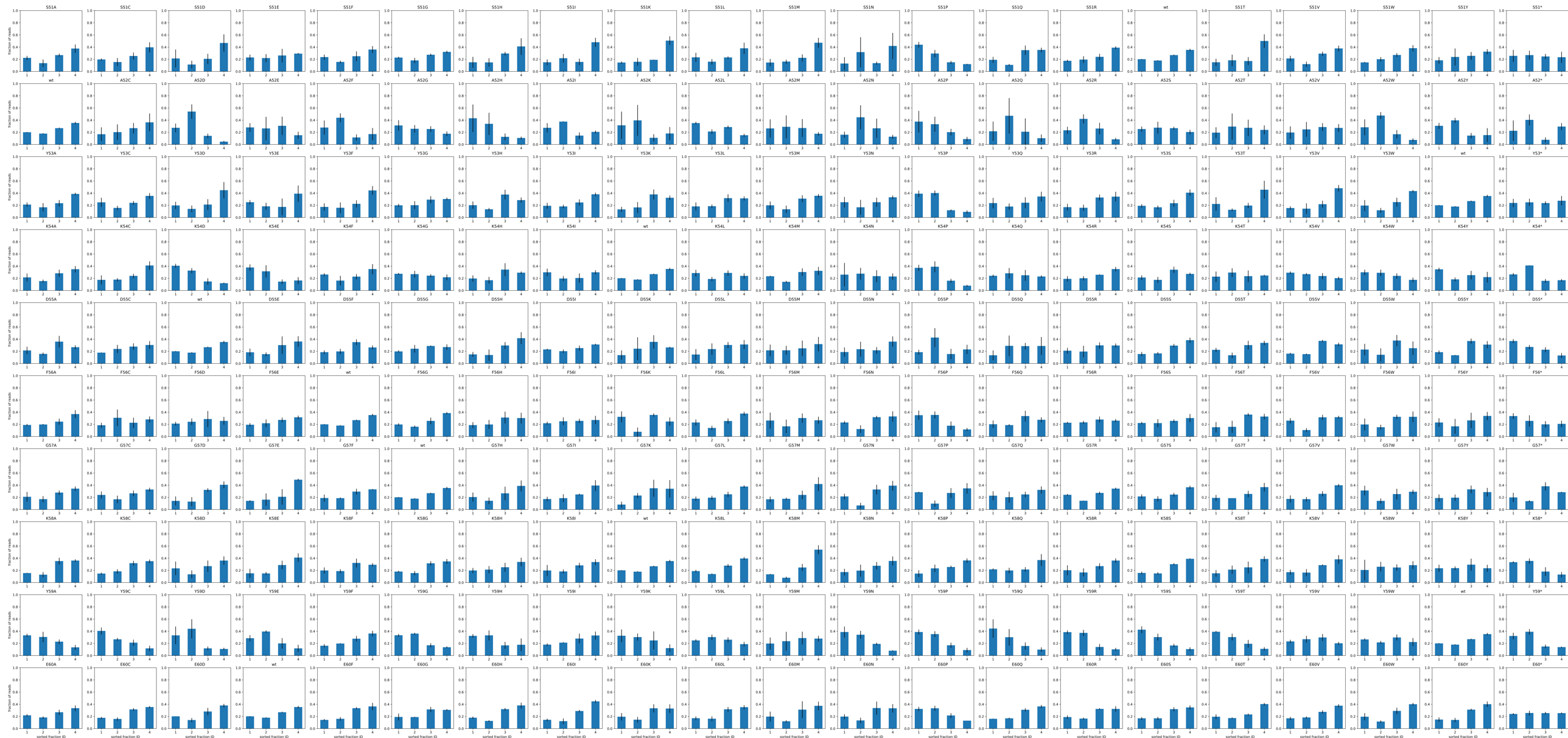

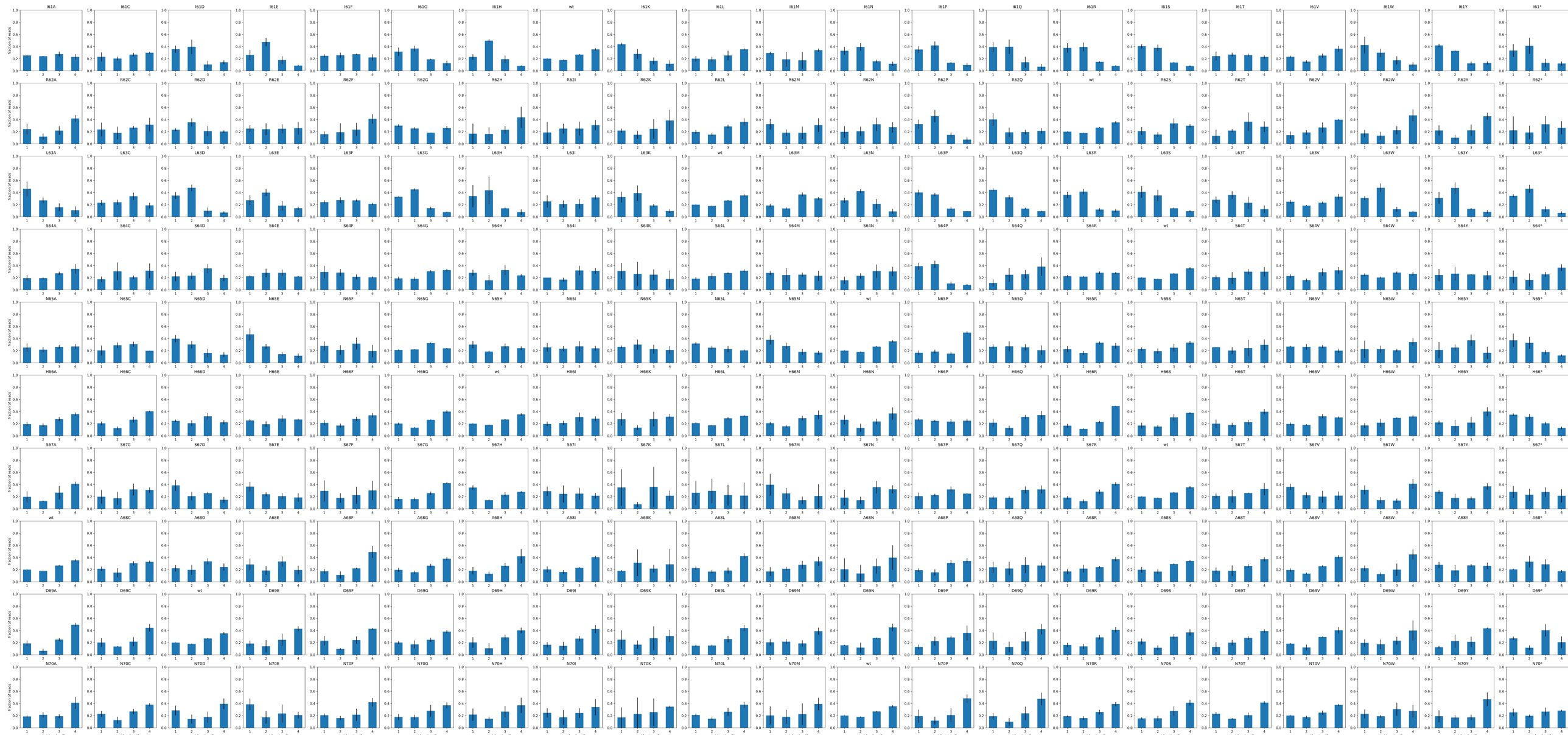

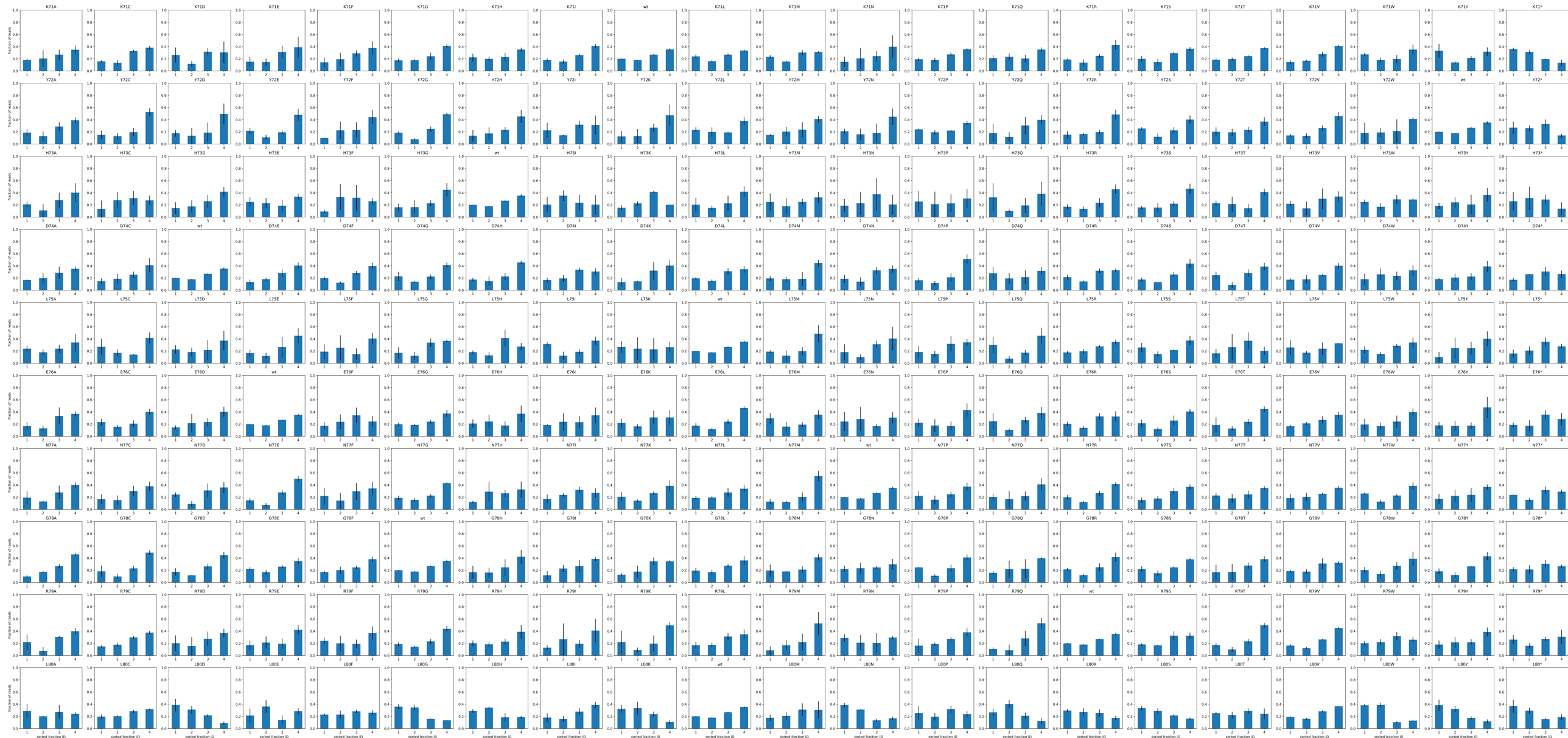

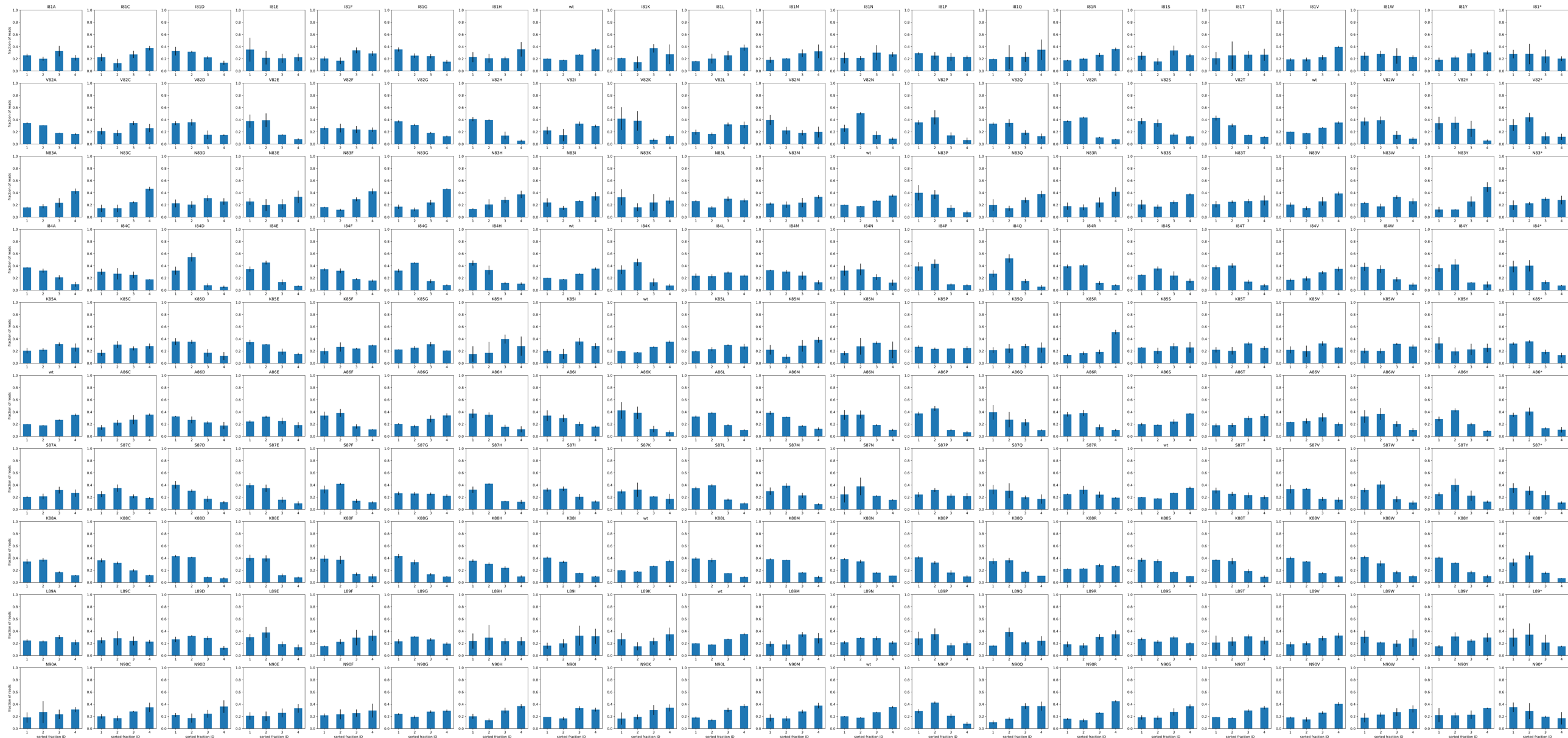

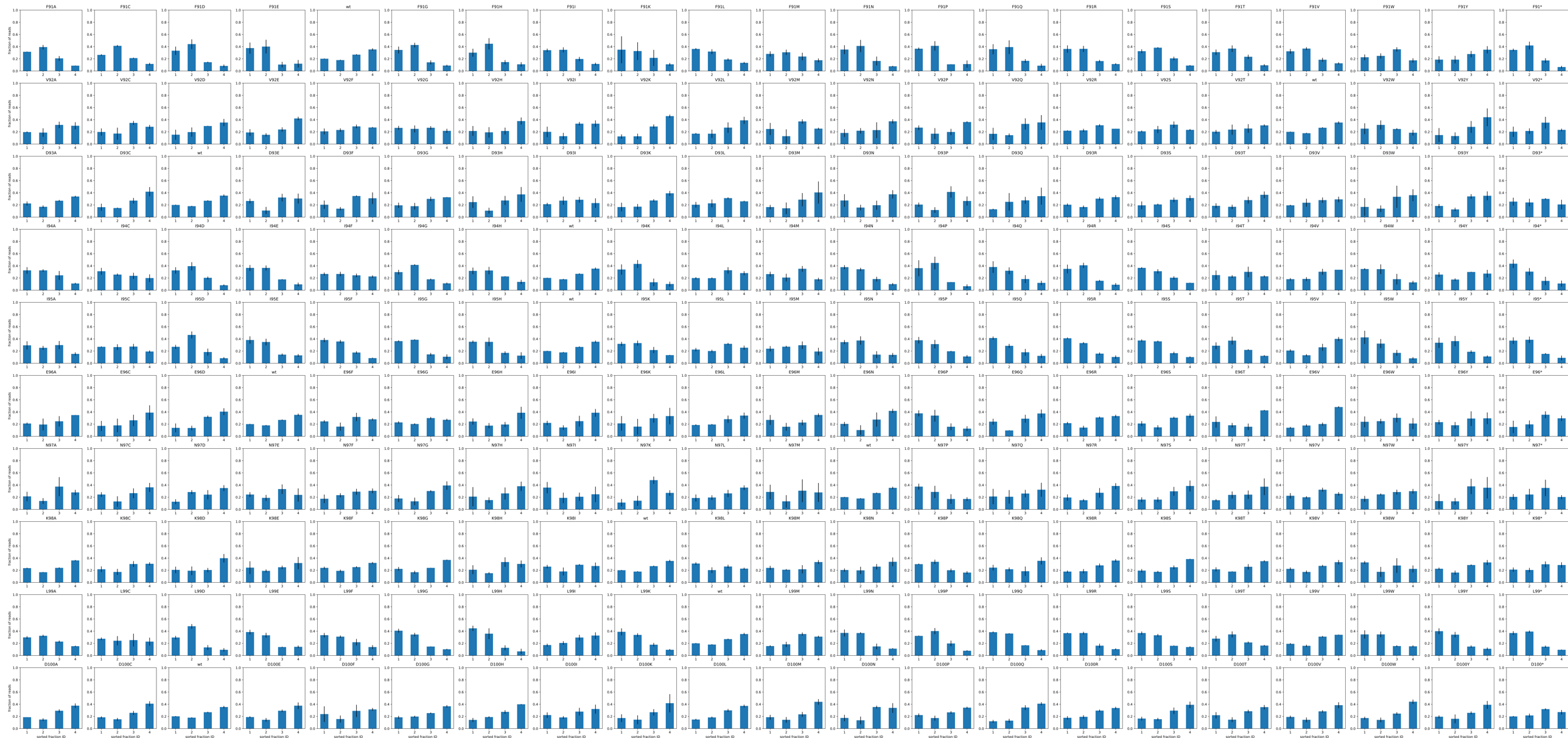

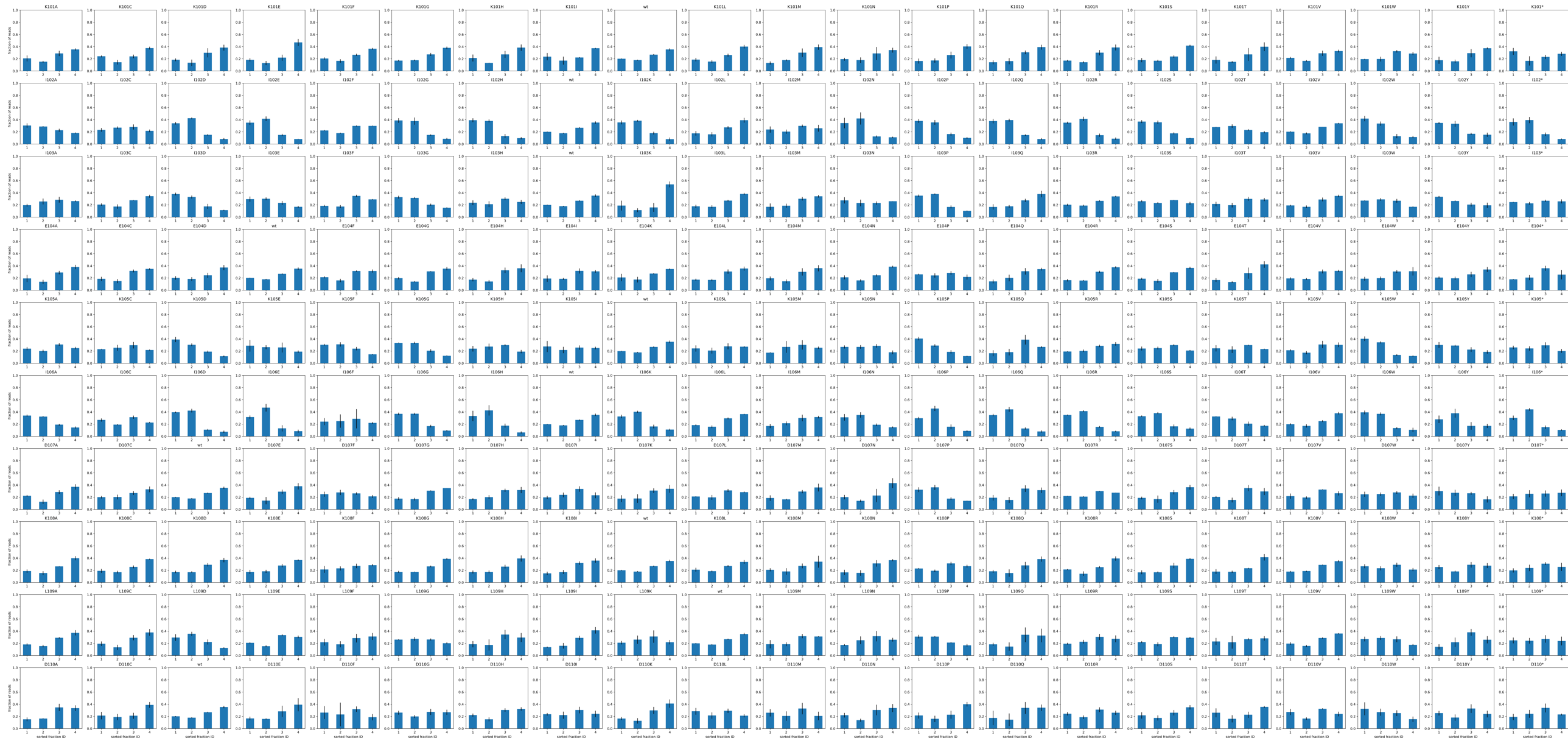

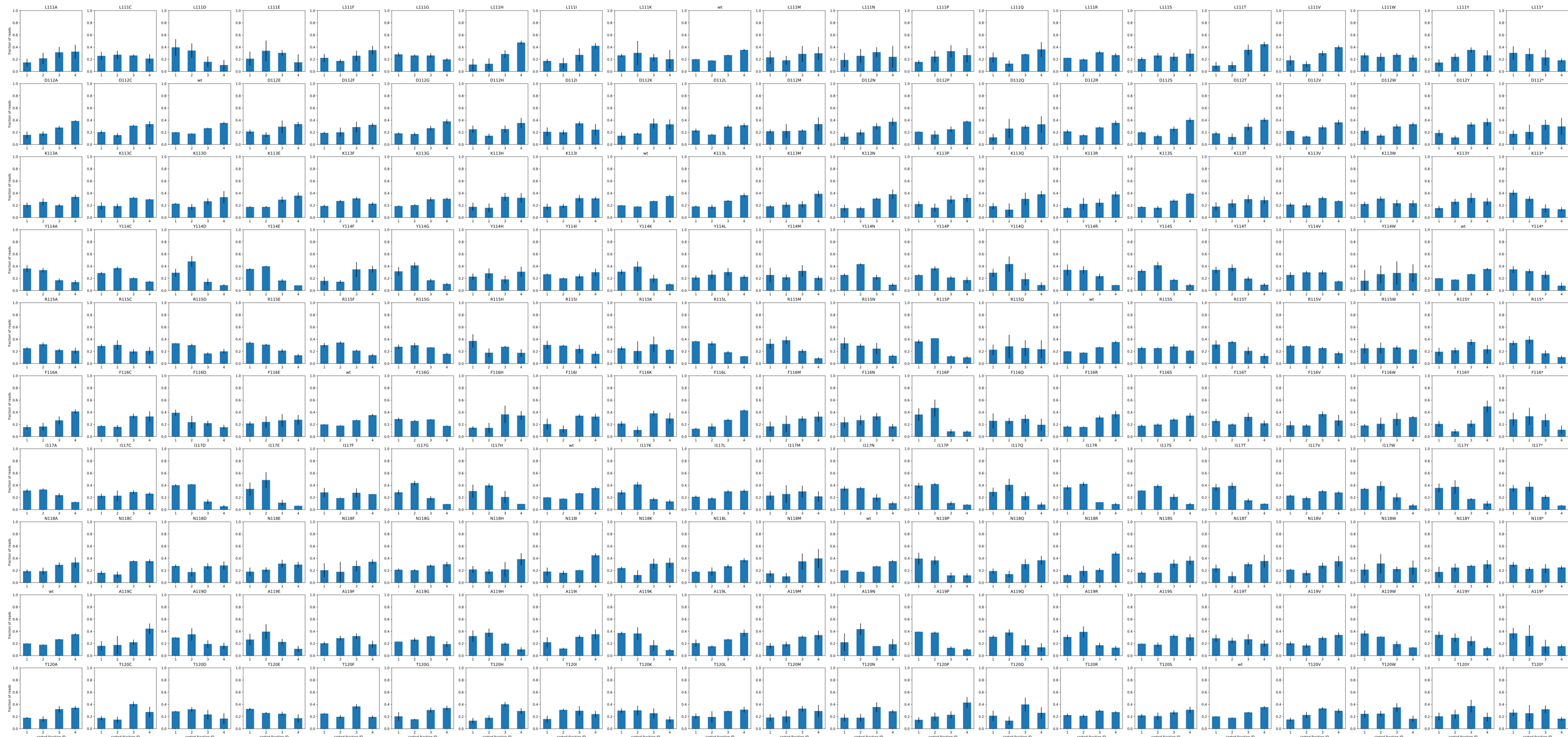

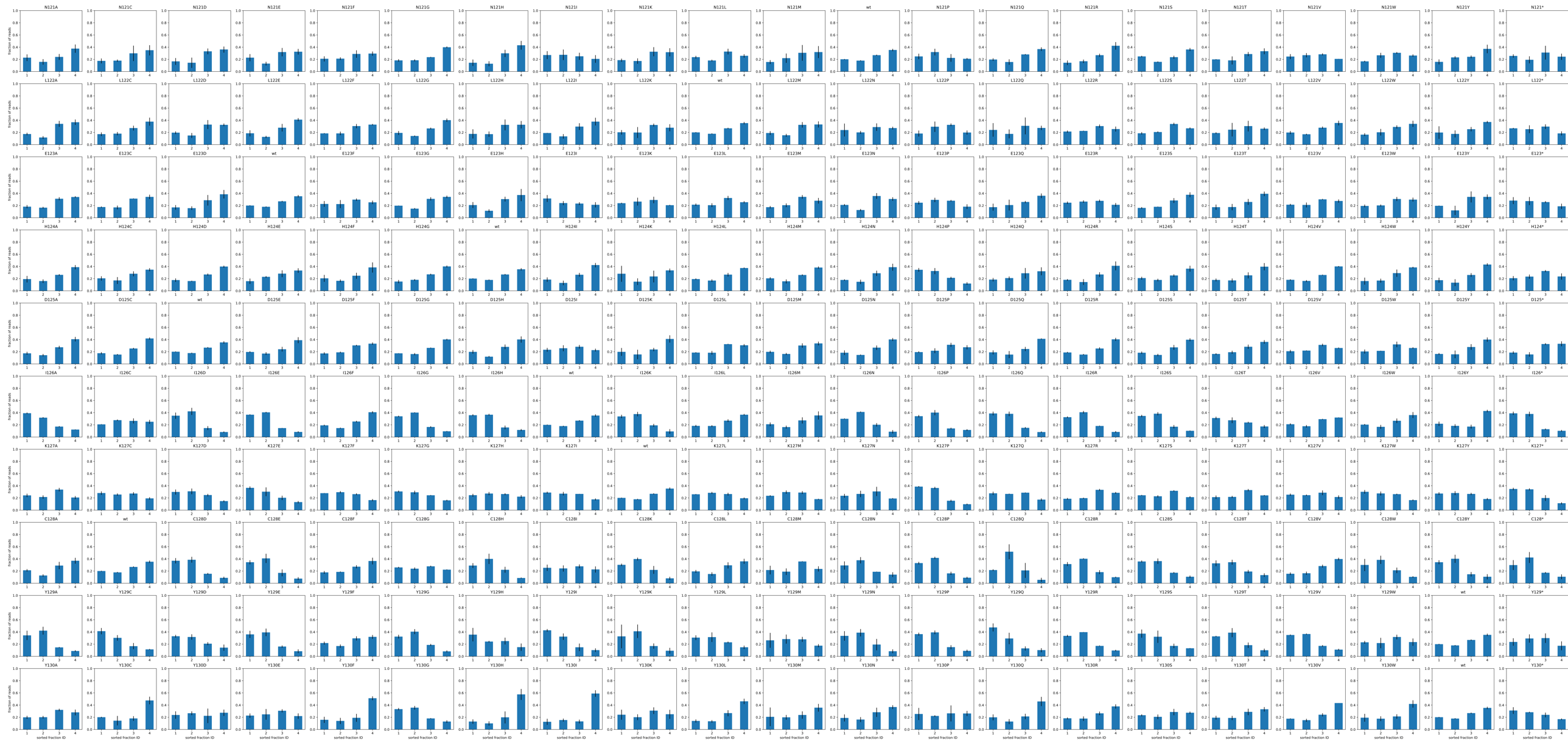

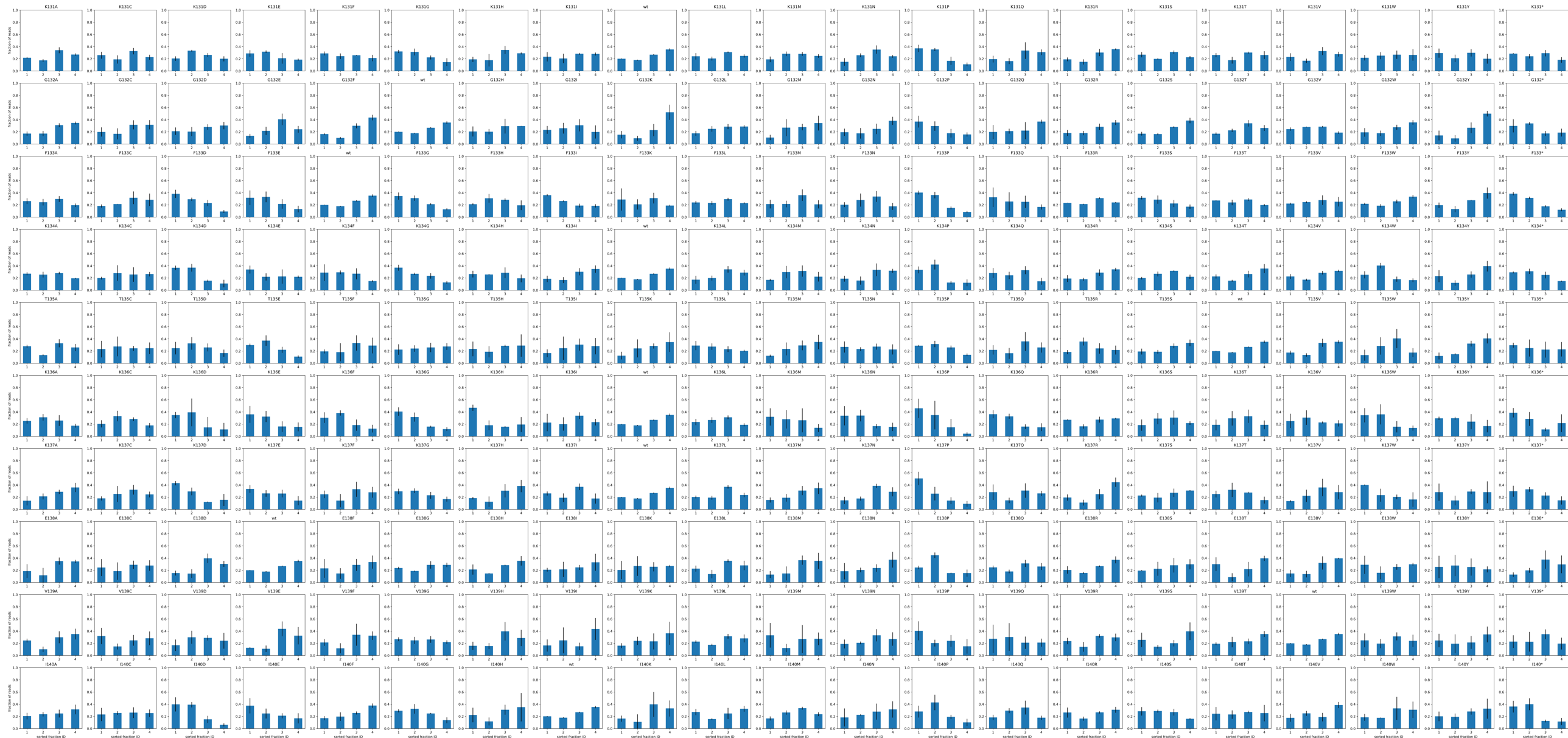

Supplement: gkae1052_Supplemental_Files [file gkae1052_supplemental_files.zip › Supplementary data 3.pdf]

**A**

fractions 1-4

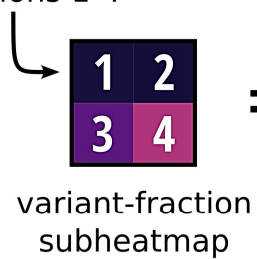

=

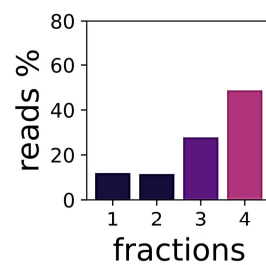**B**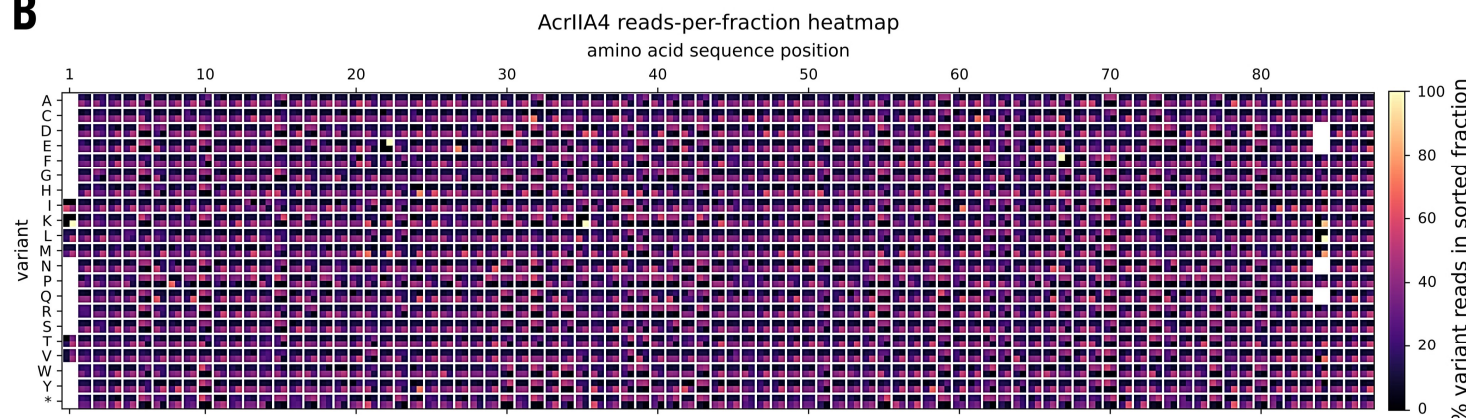**C**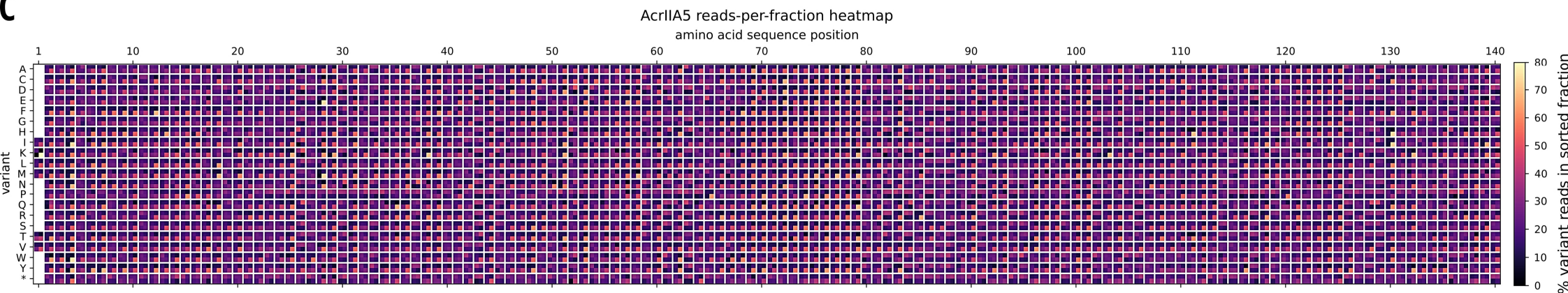

Supplement: gkae1052_Supplemental_Files [file gkae1052_supplemental_files.zip › Supplementary data 4_revised.pdf]

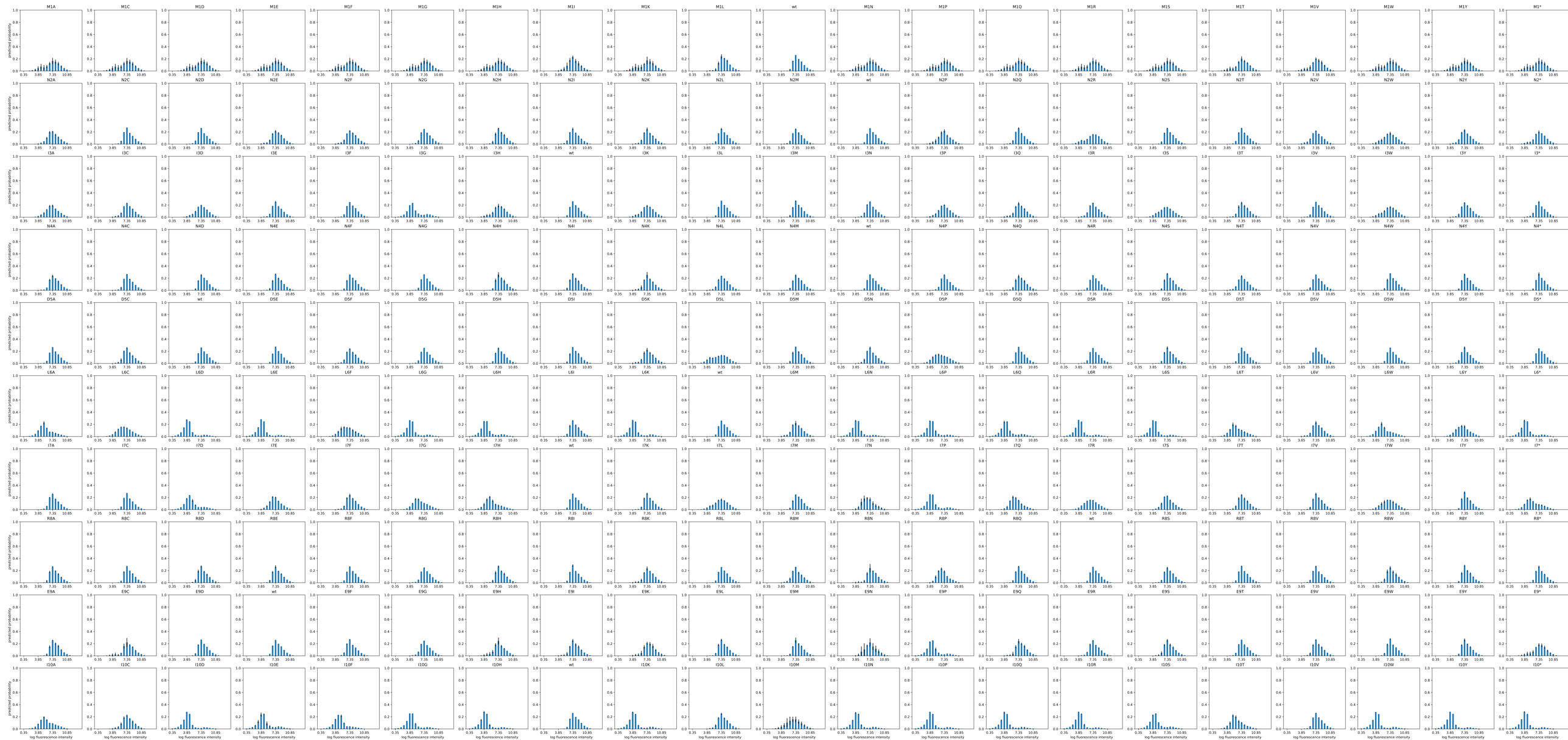

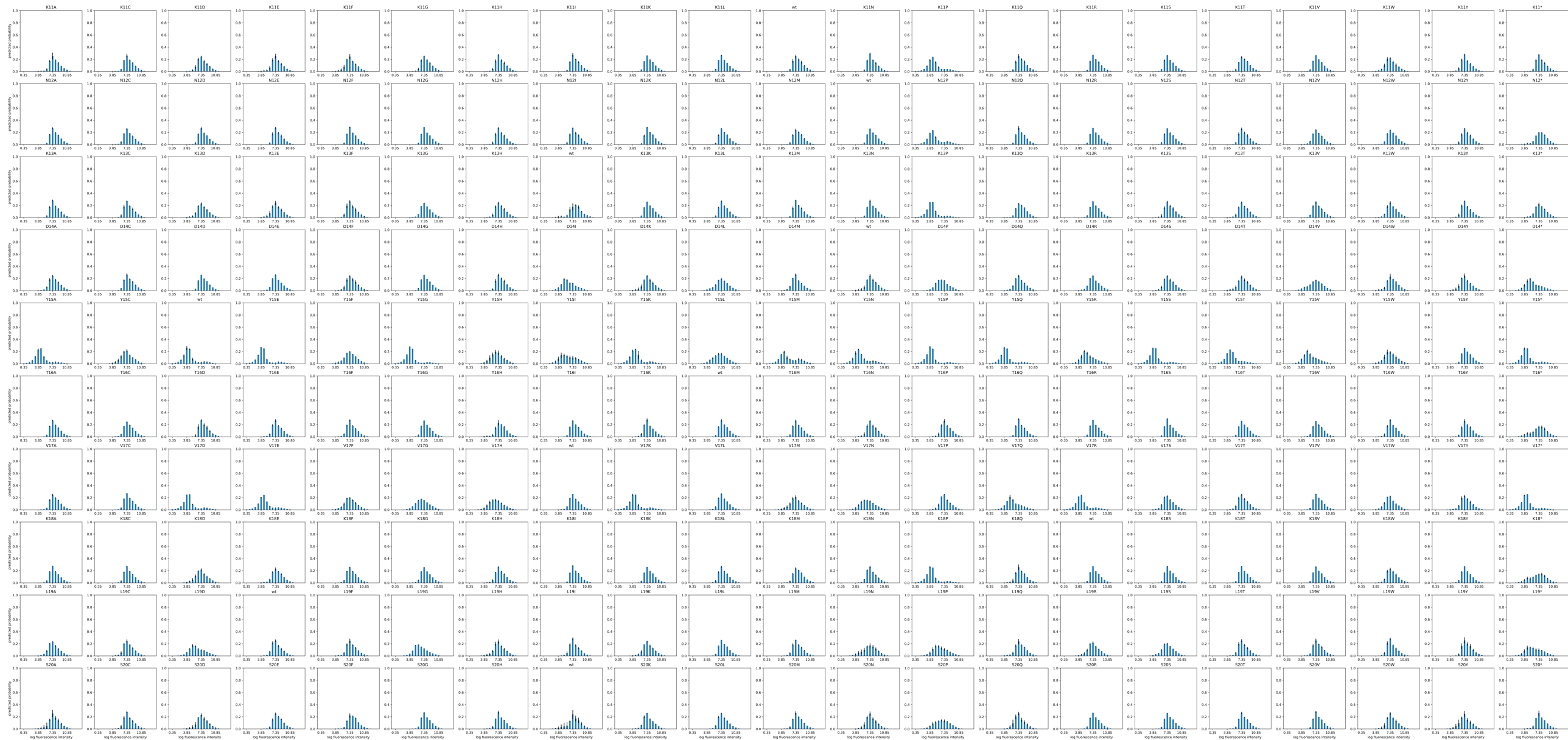

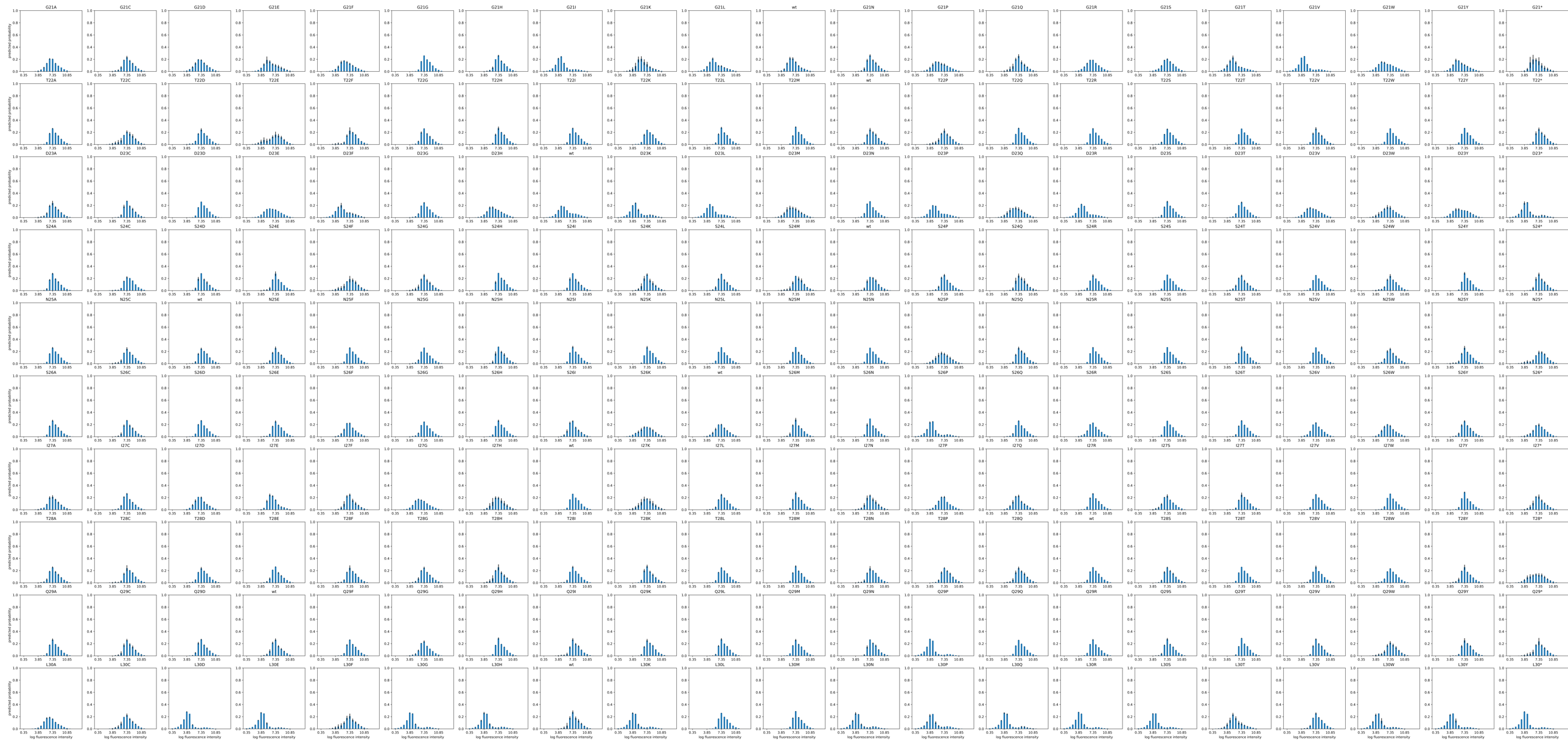

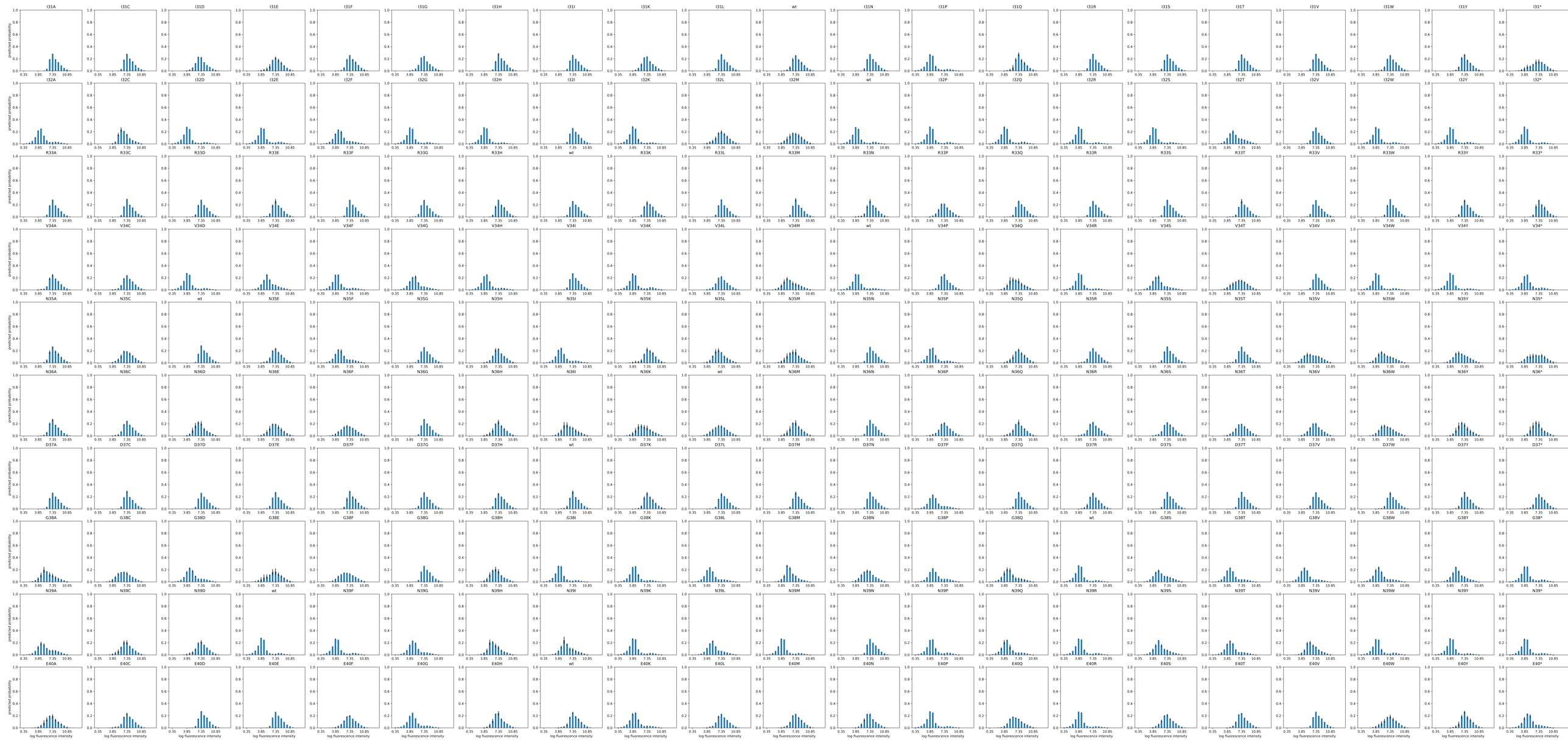

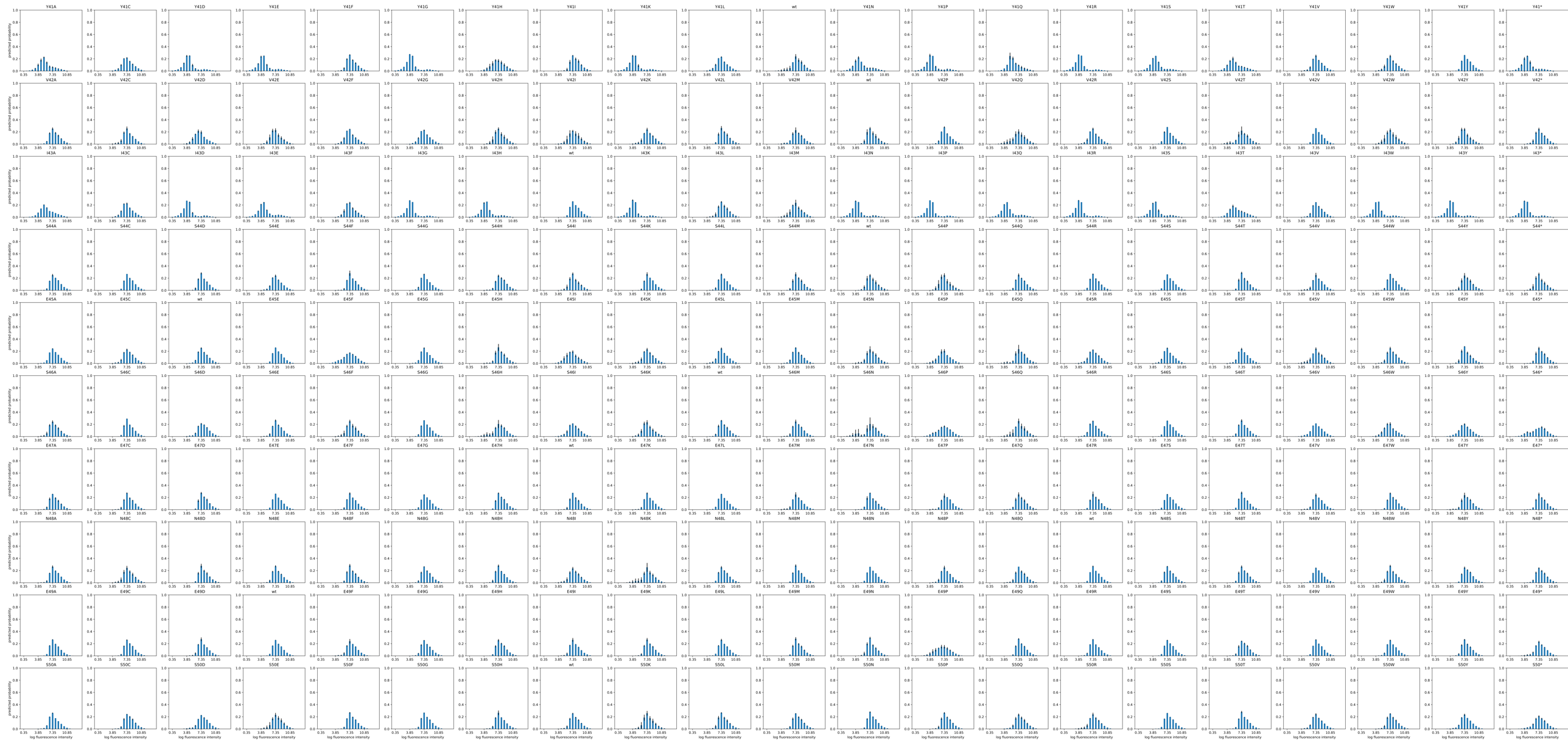

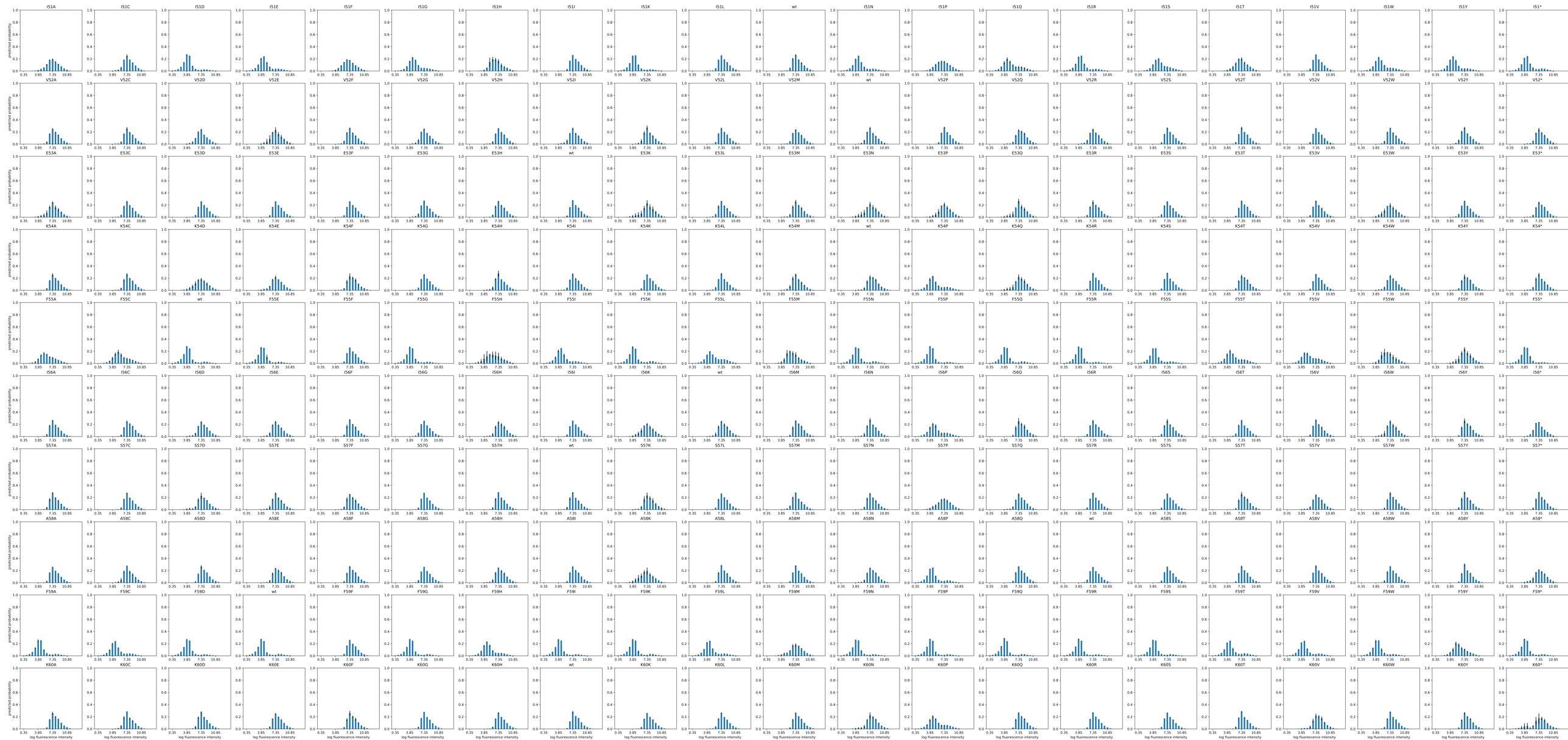

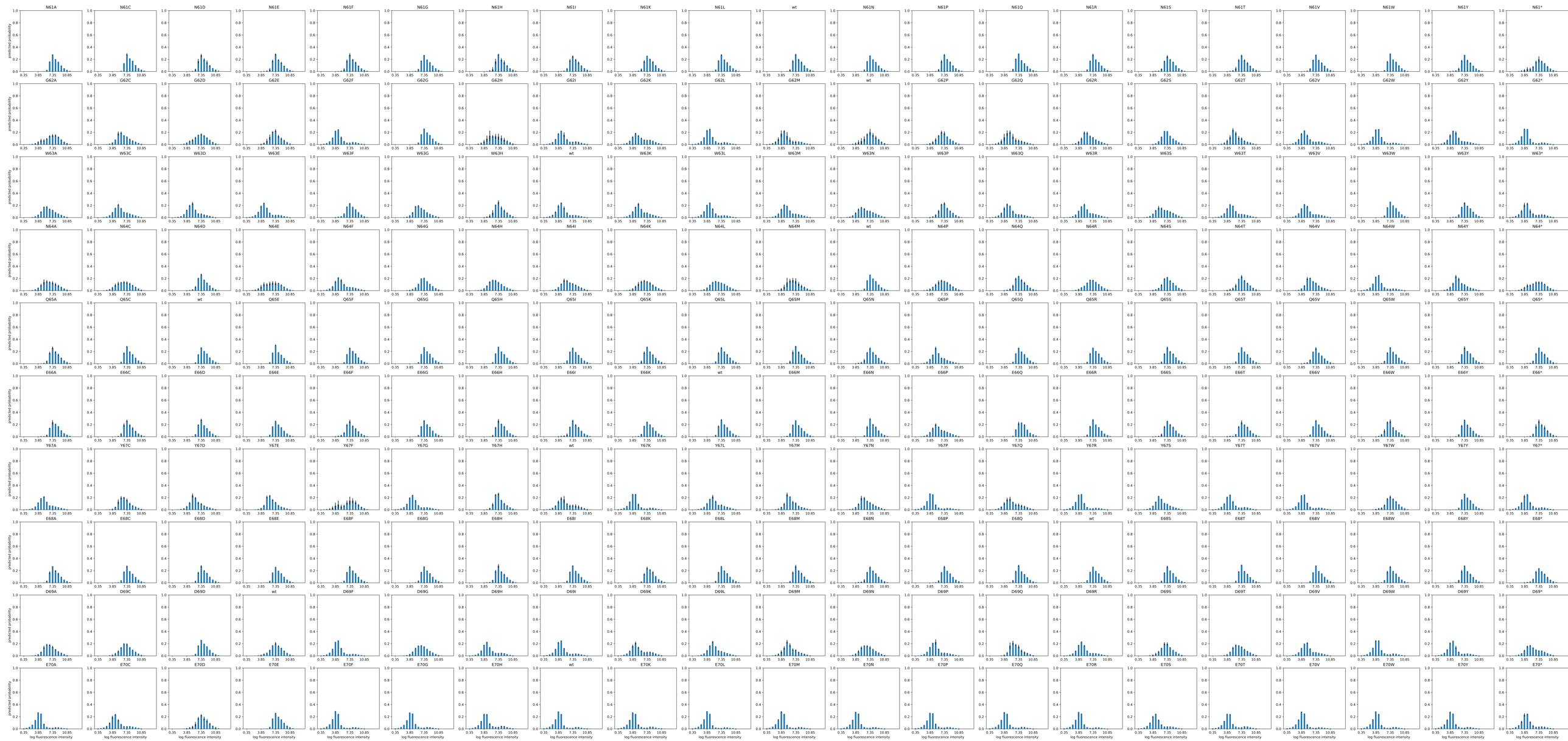

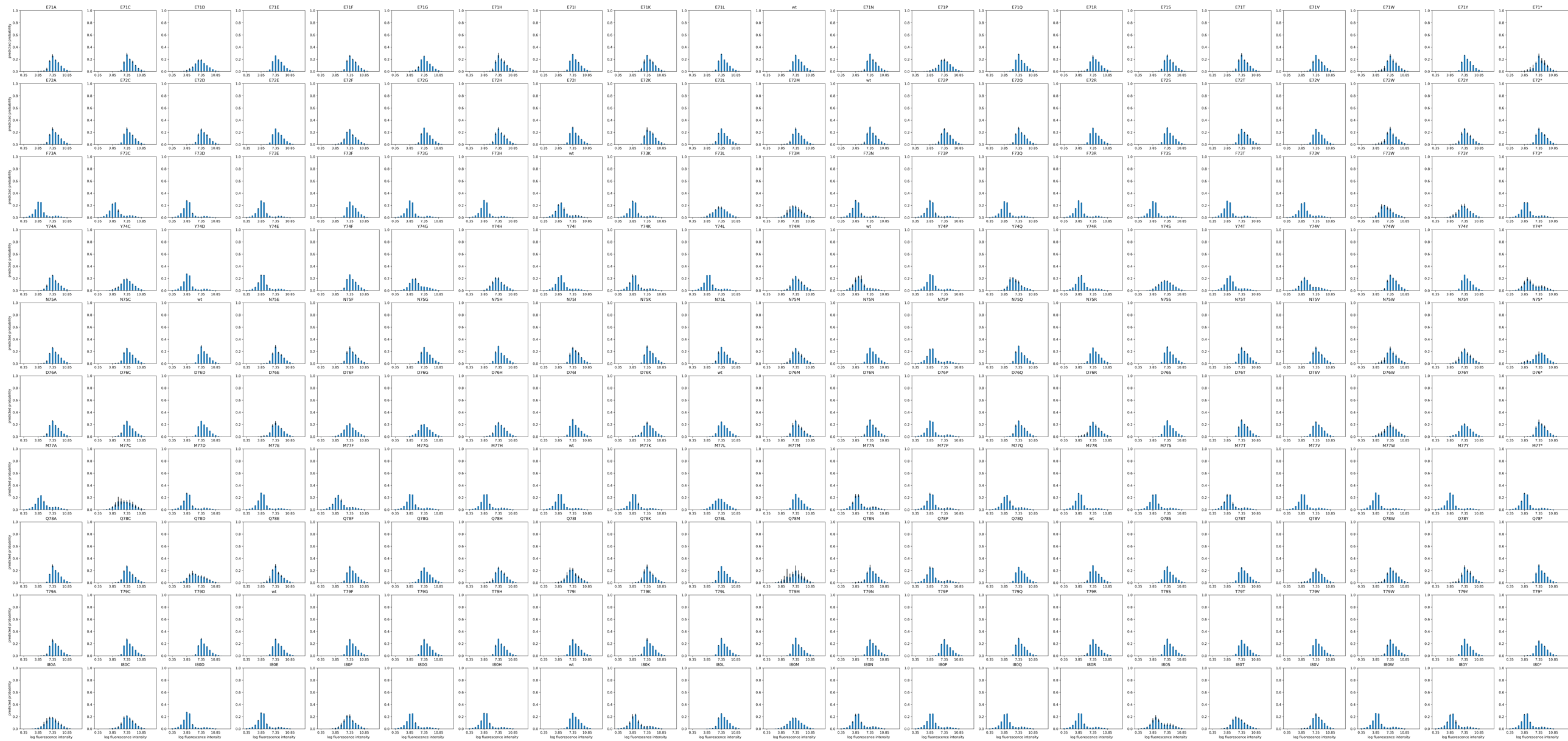

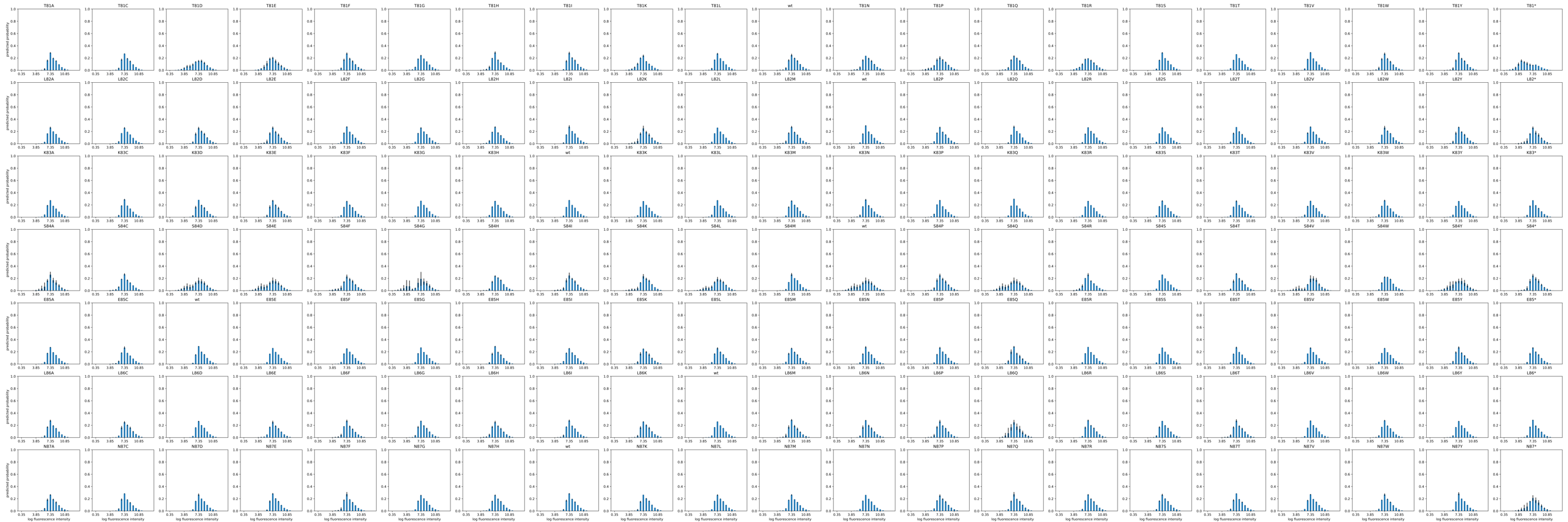

Supplement: gkae1052_Supplemental_Files [file gkae1052_supplemental_files.zip › Supplementary data 5.pdf]

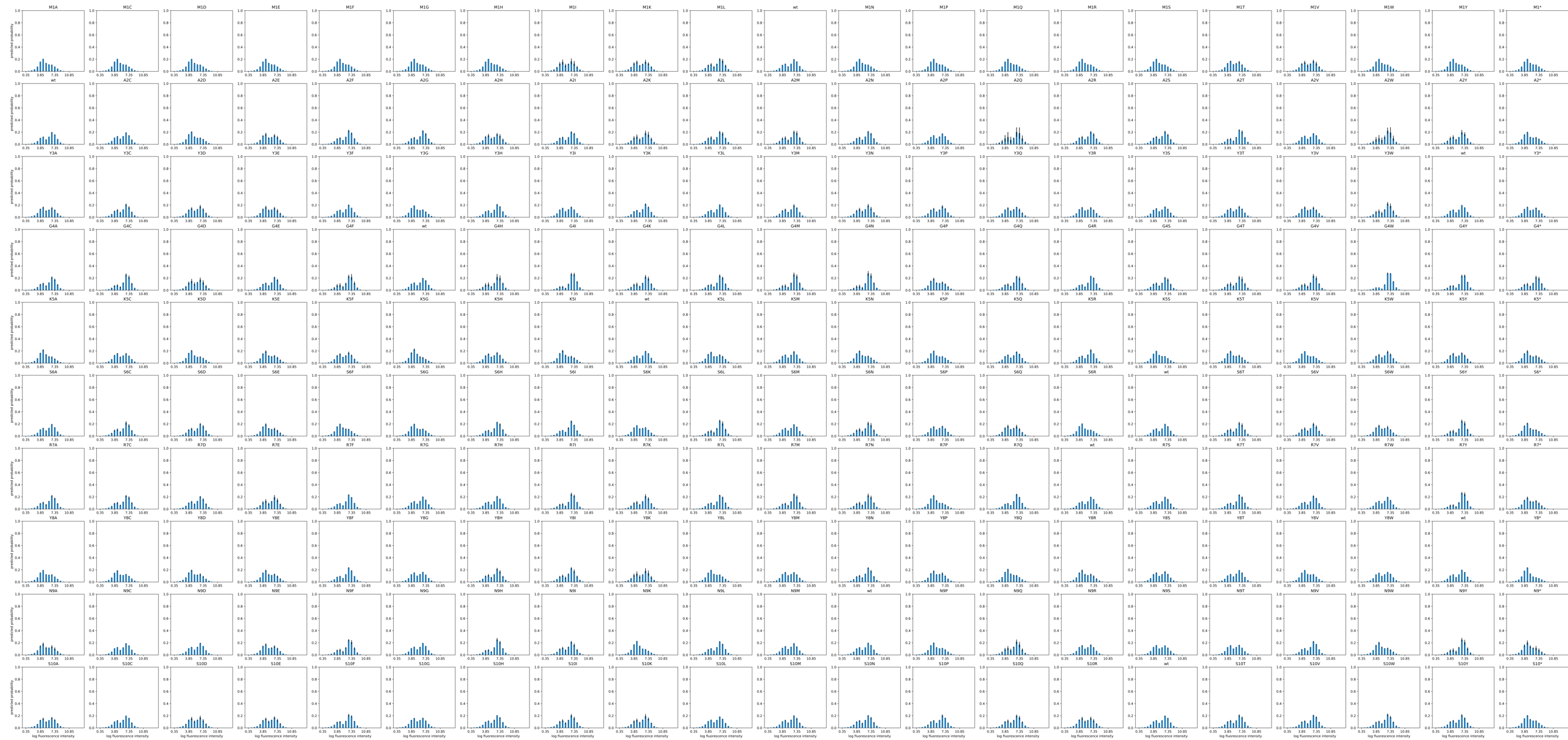

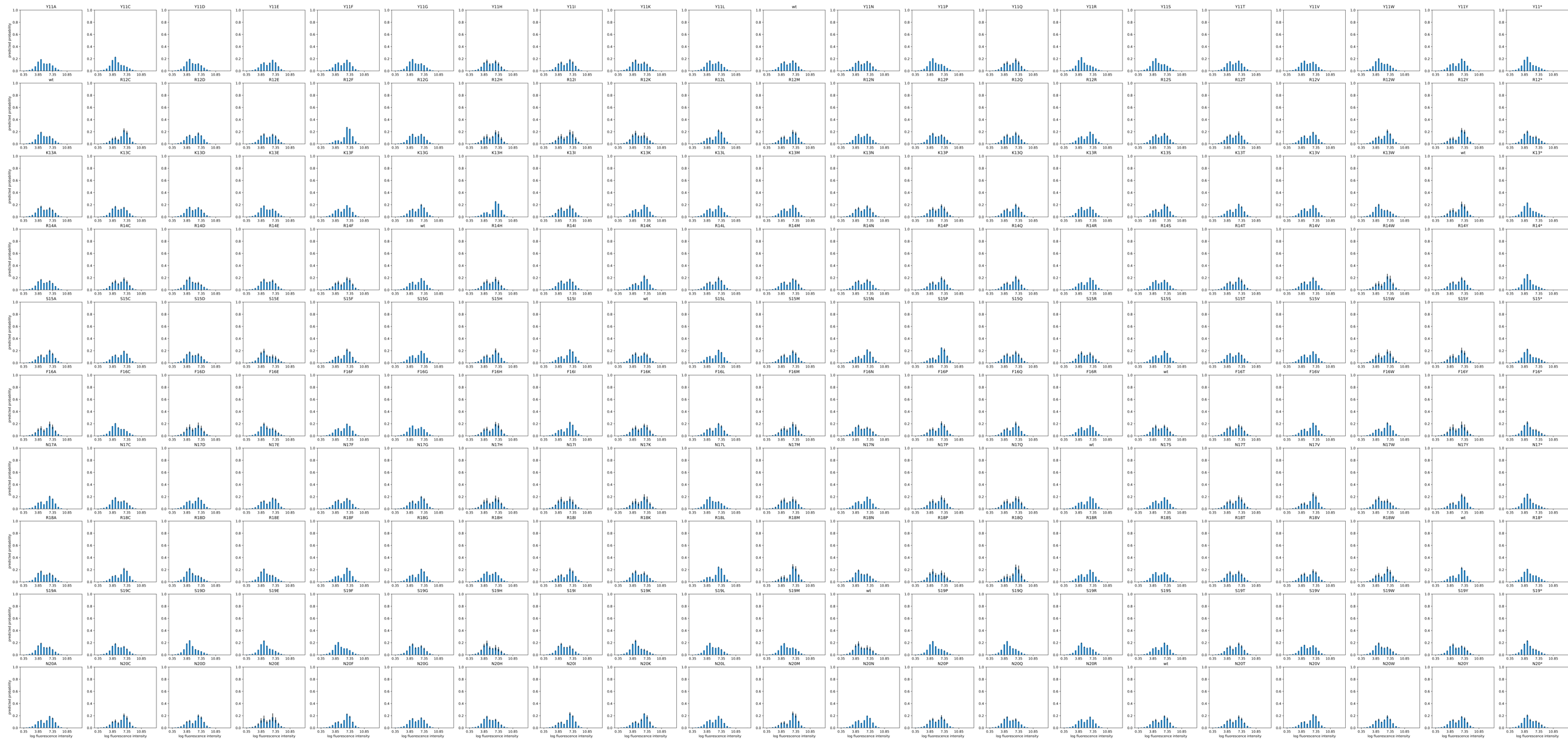

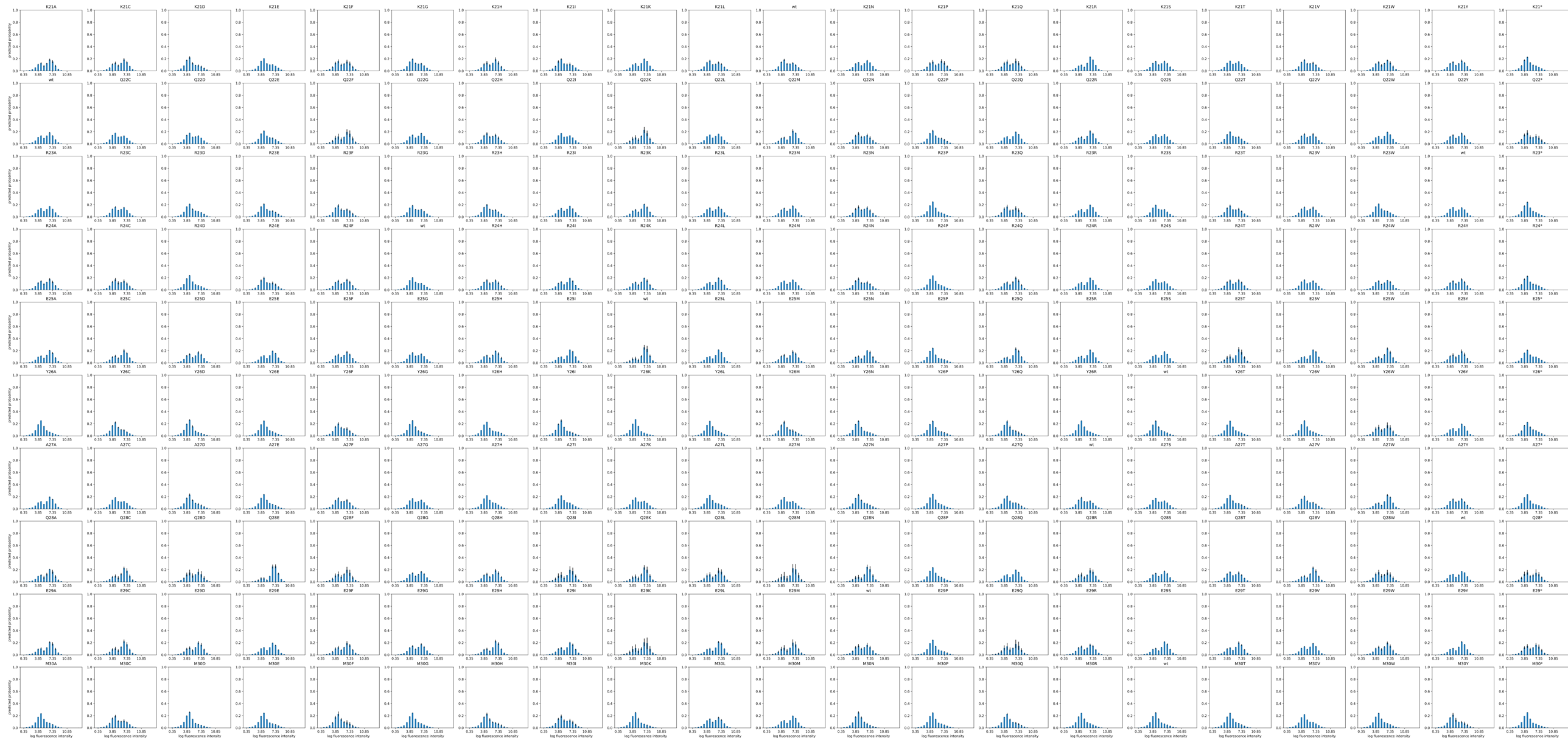

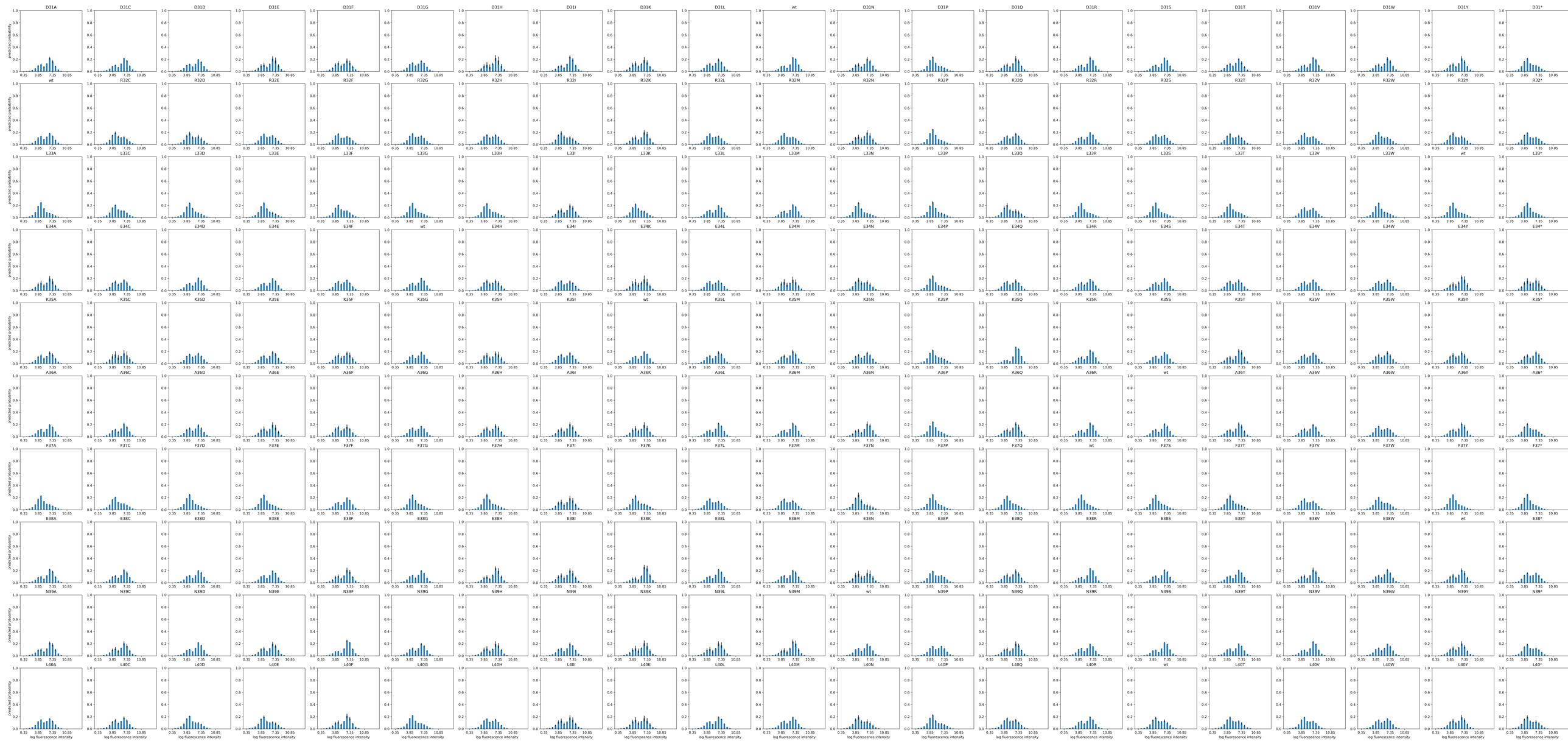

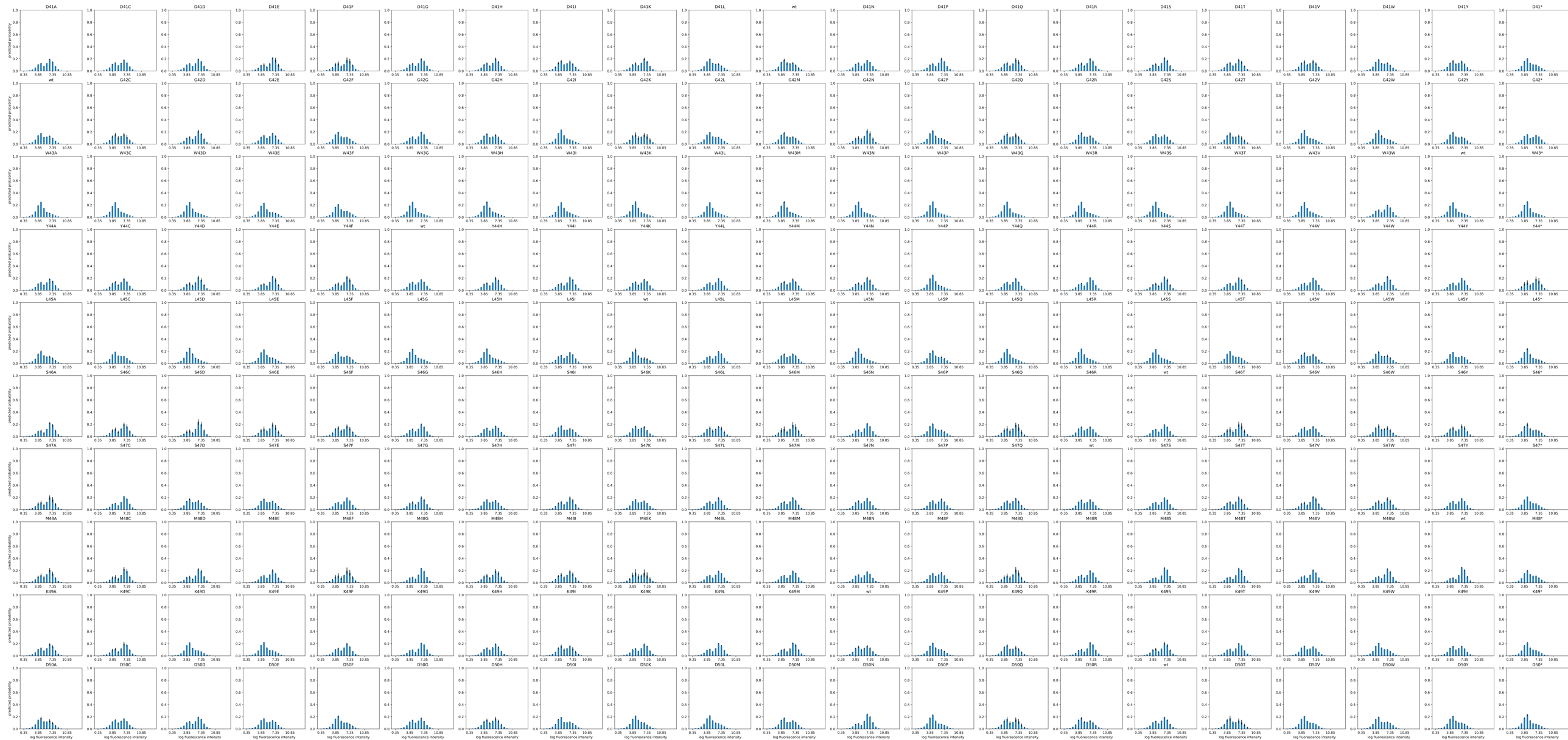

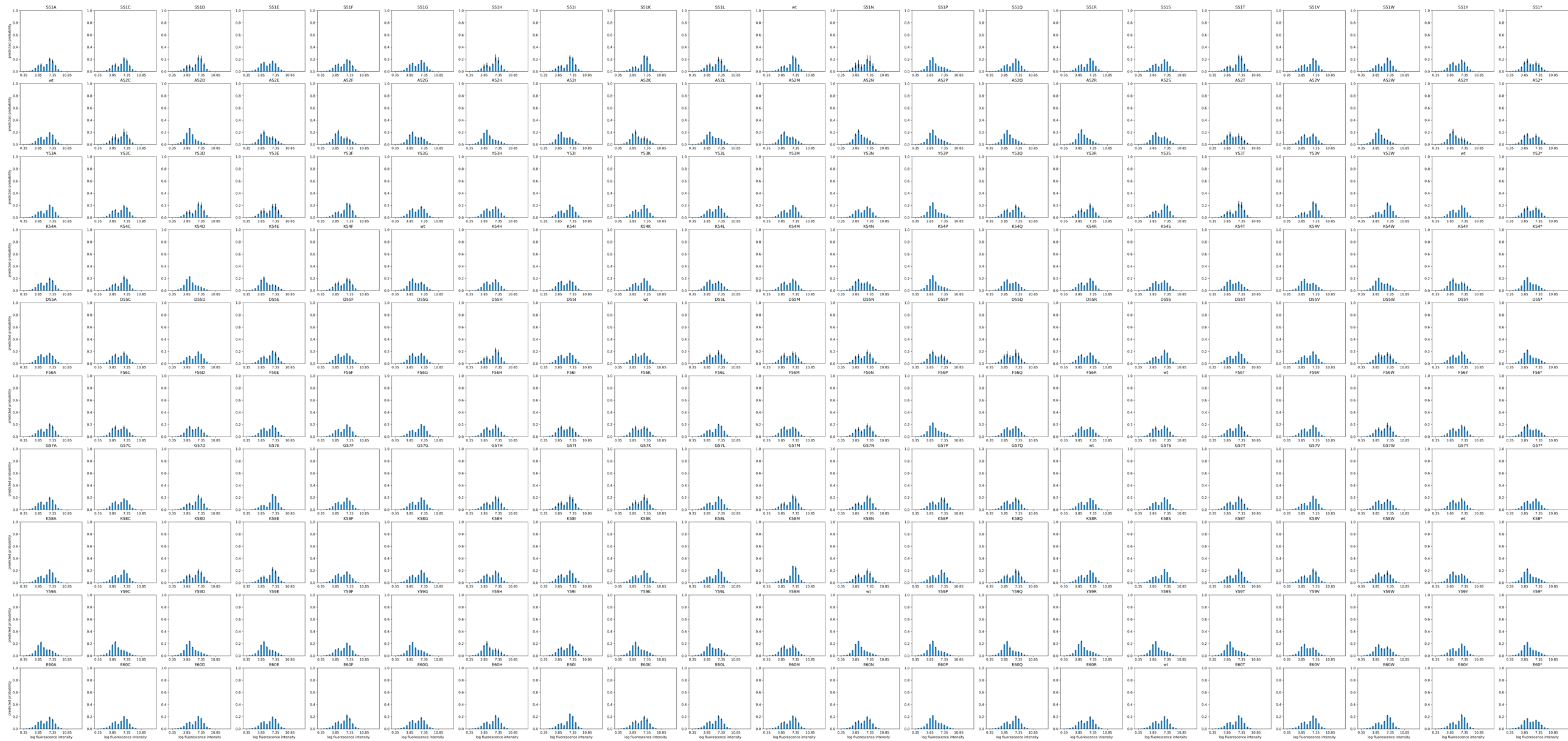

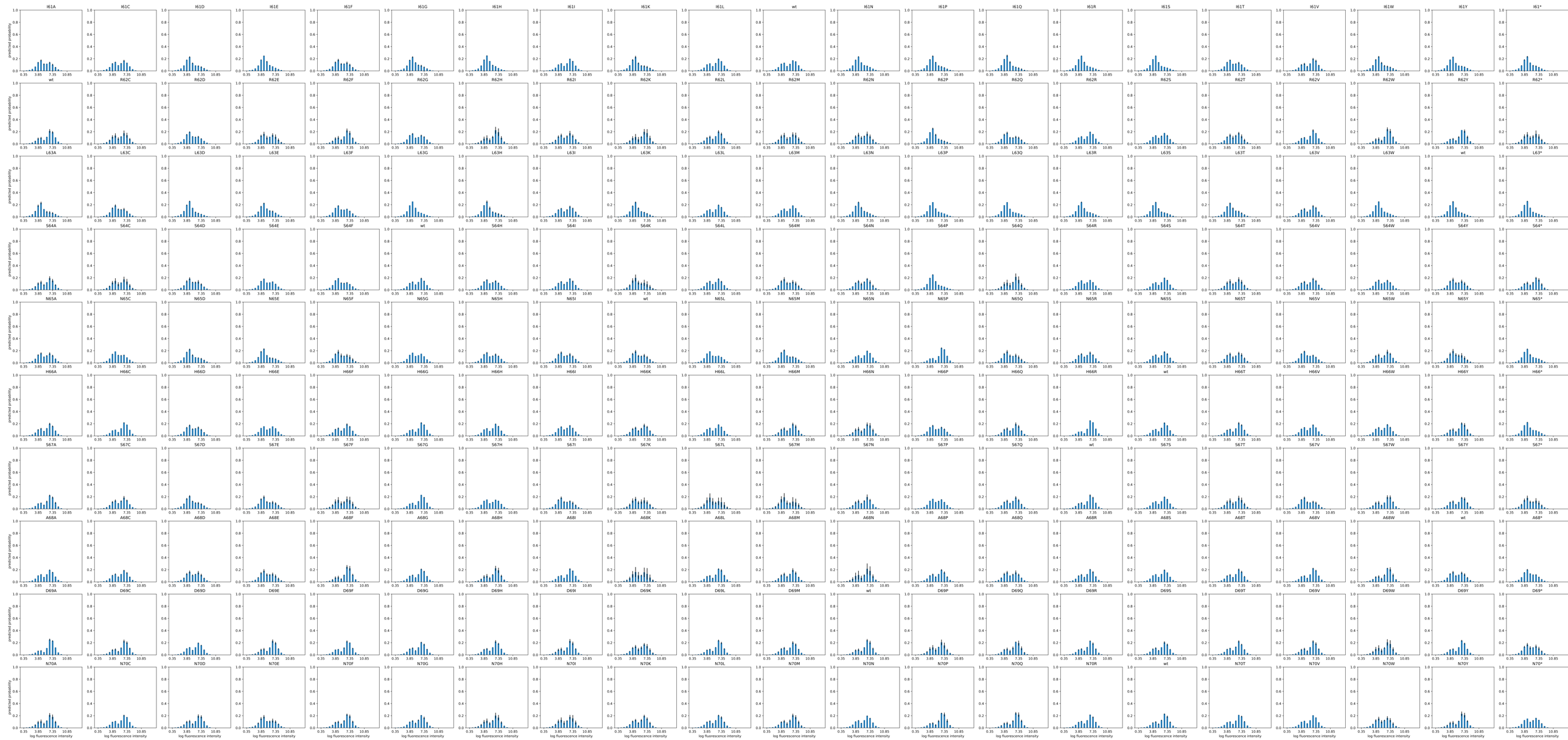

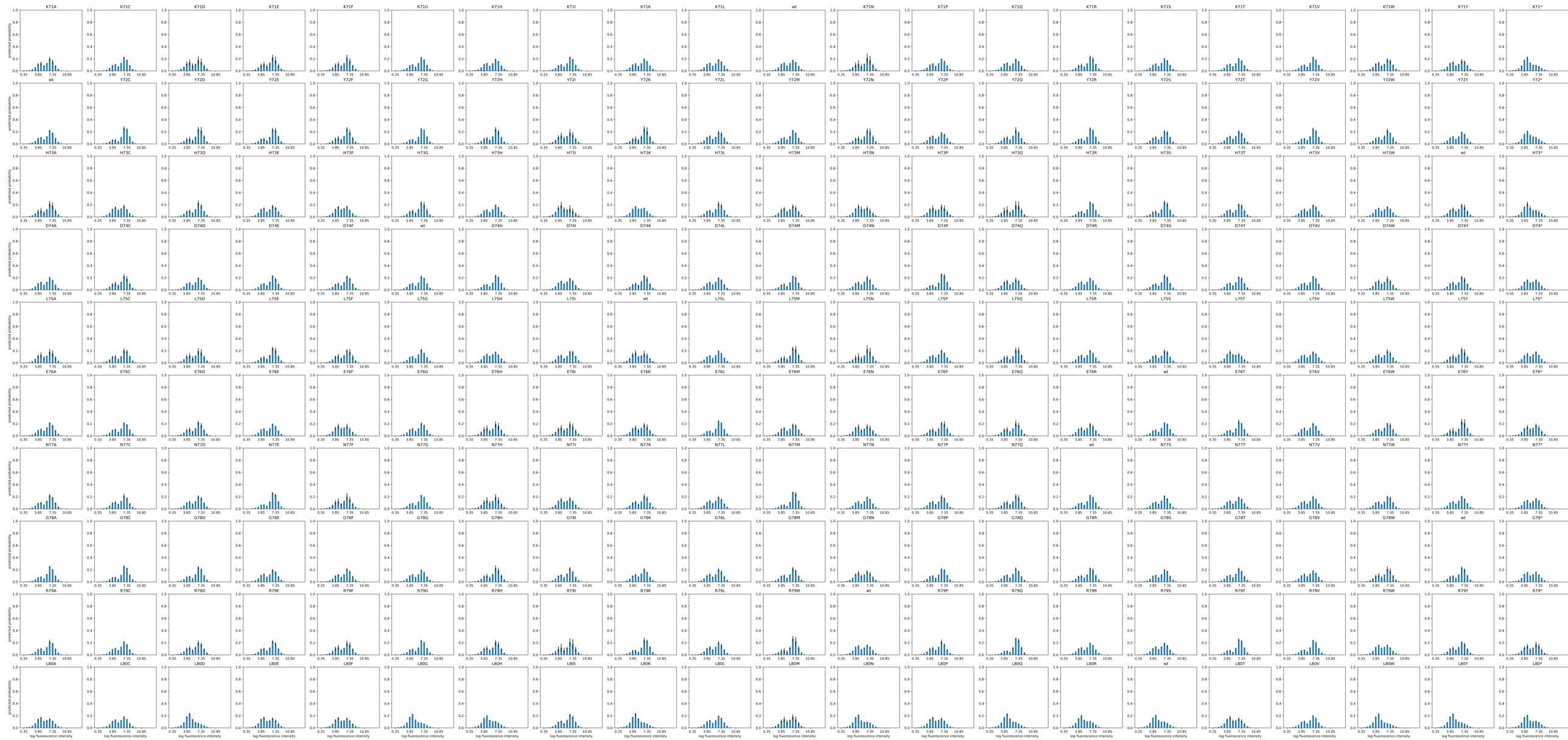

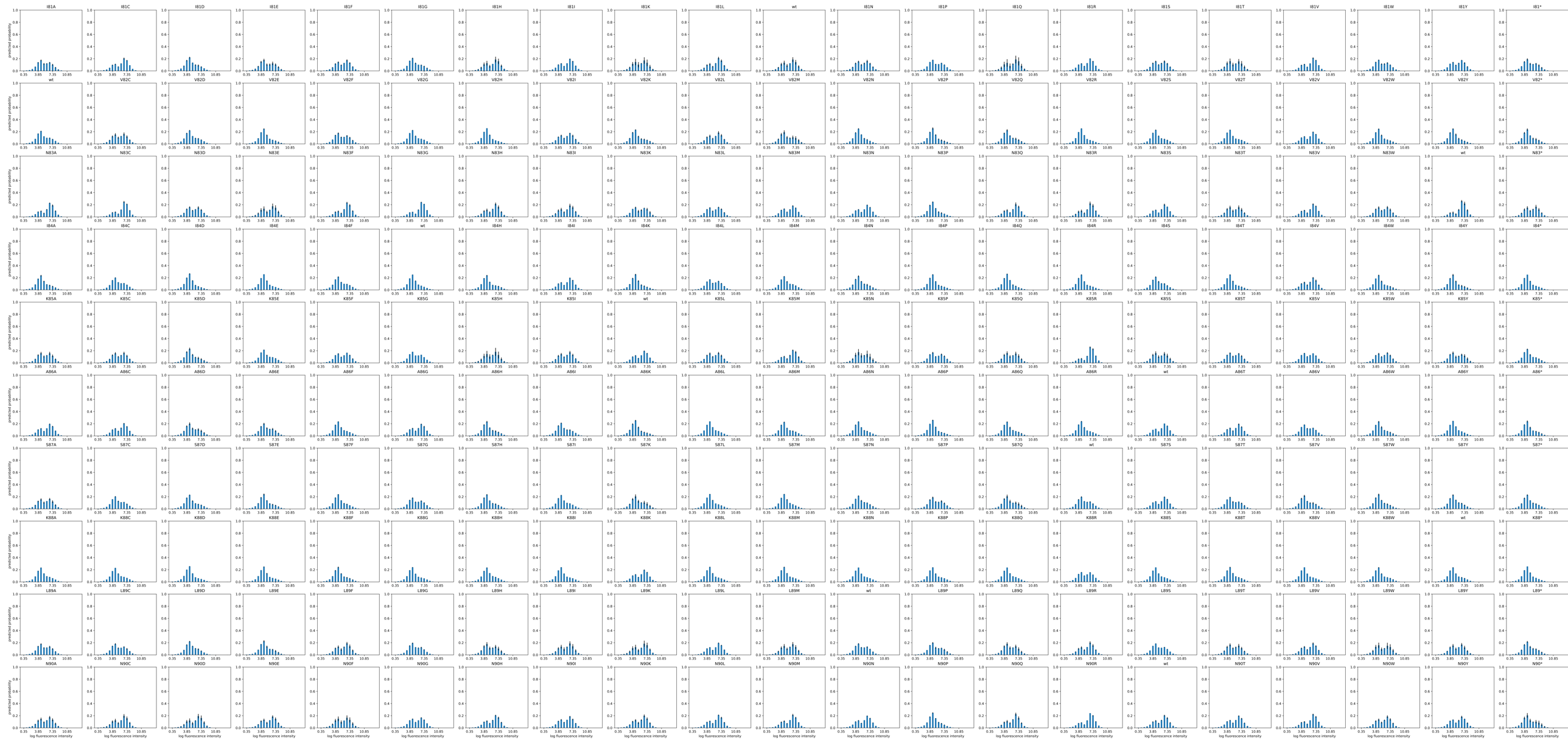

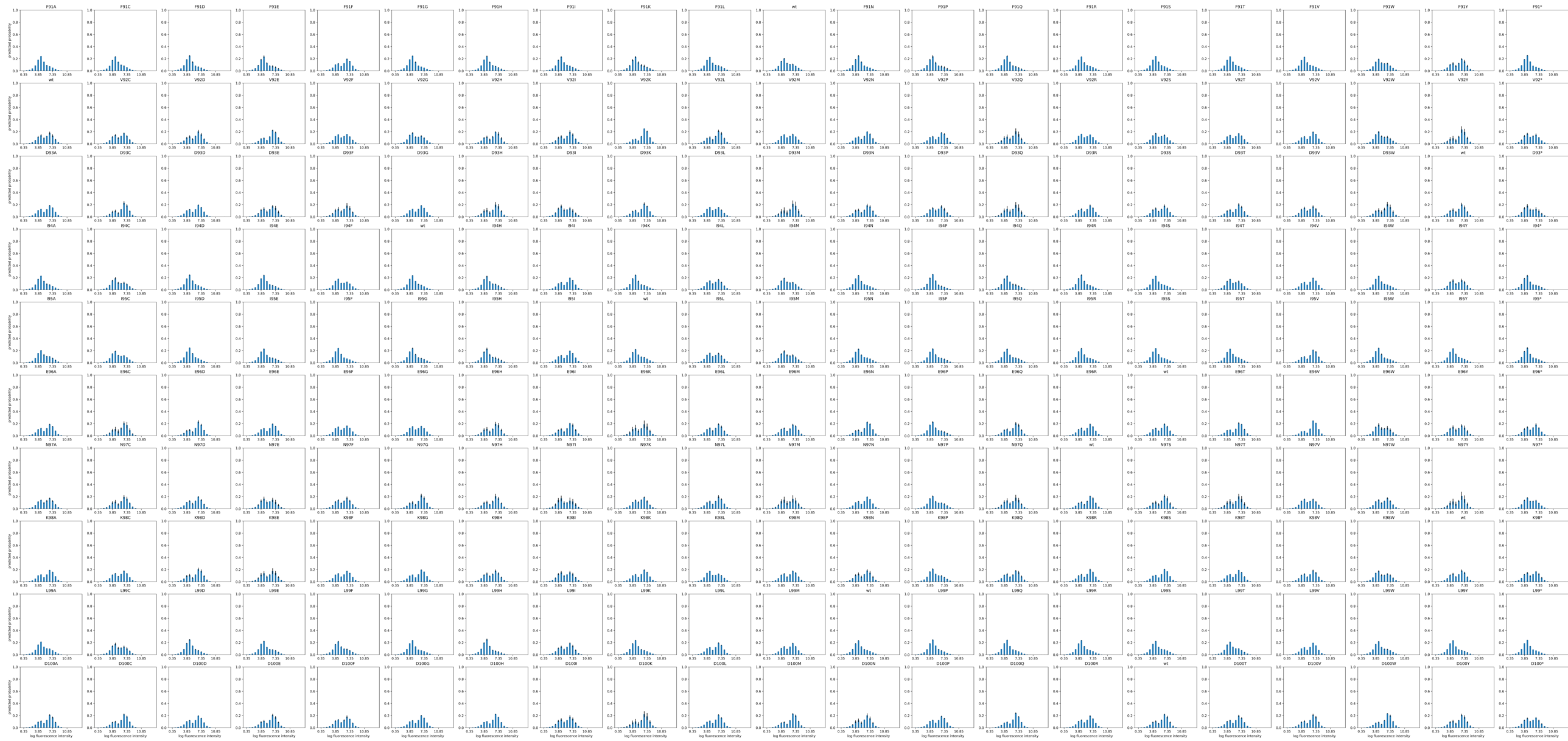

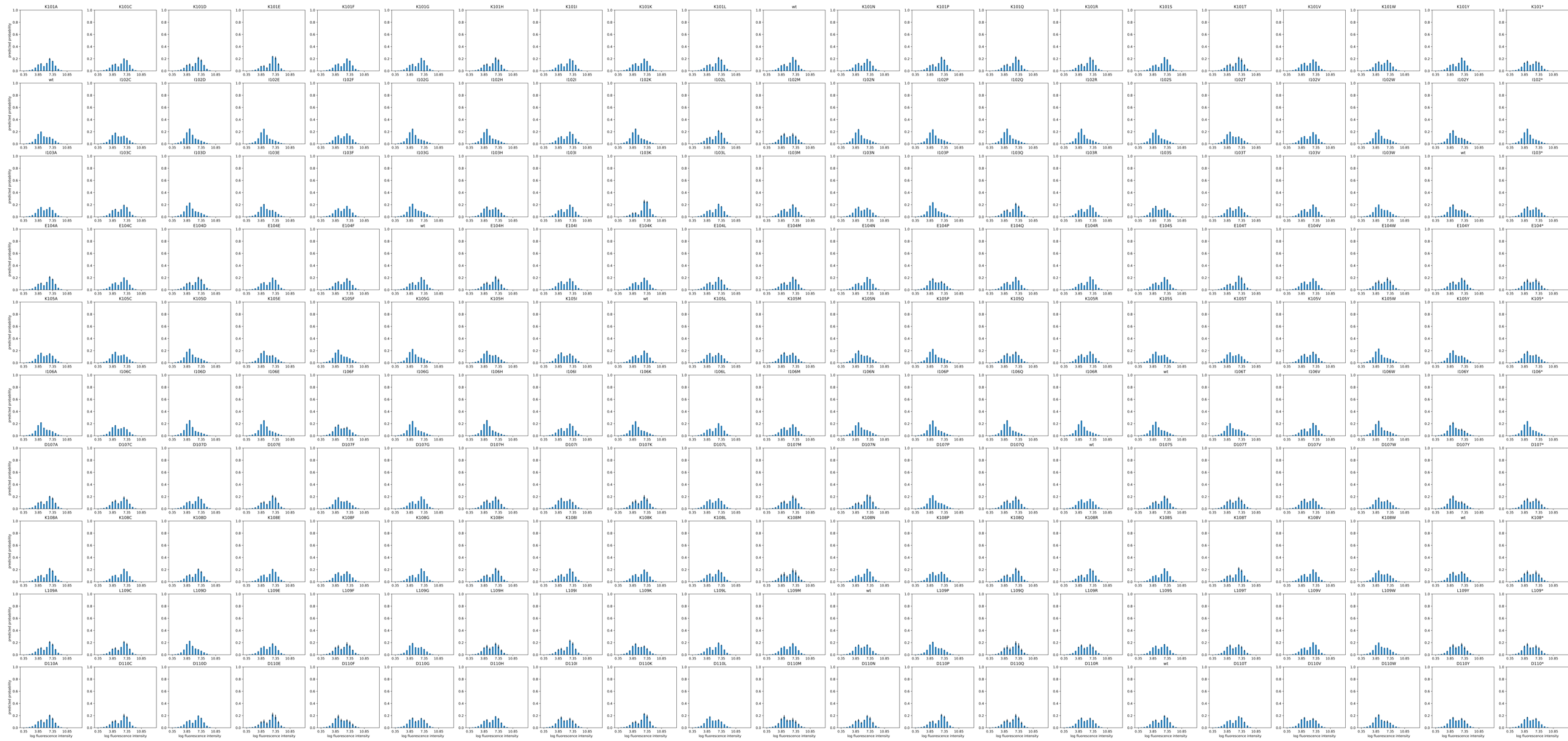

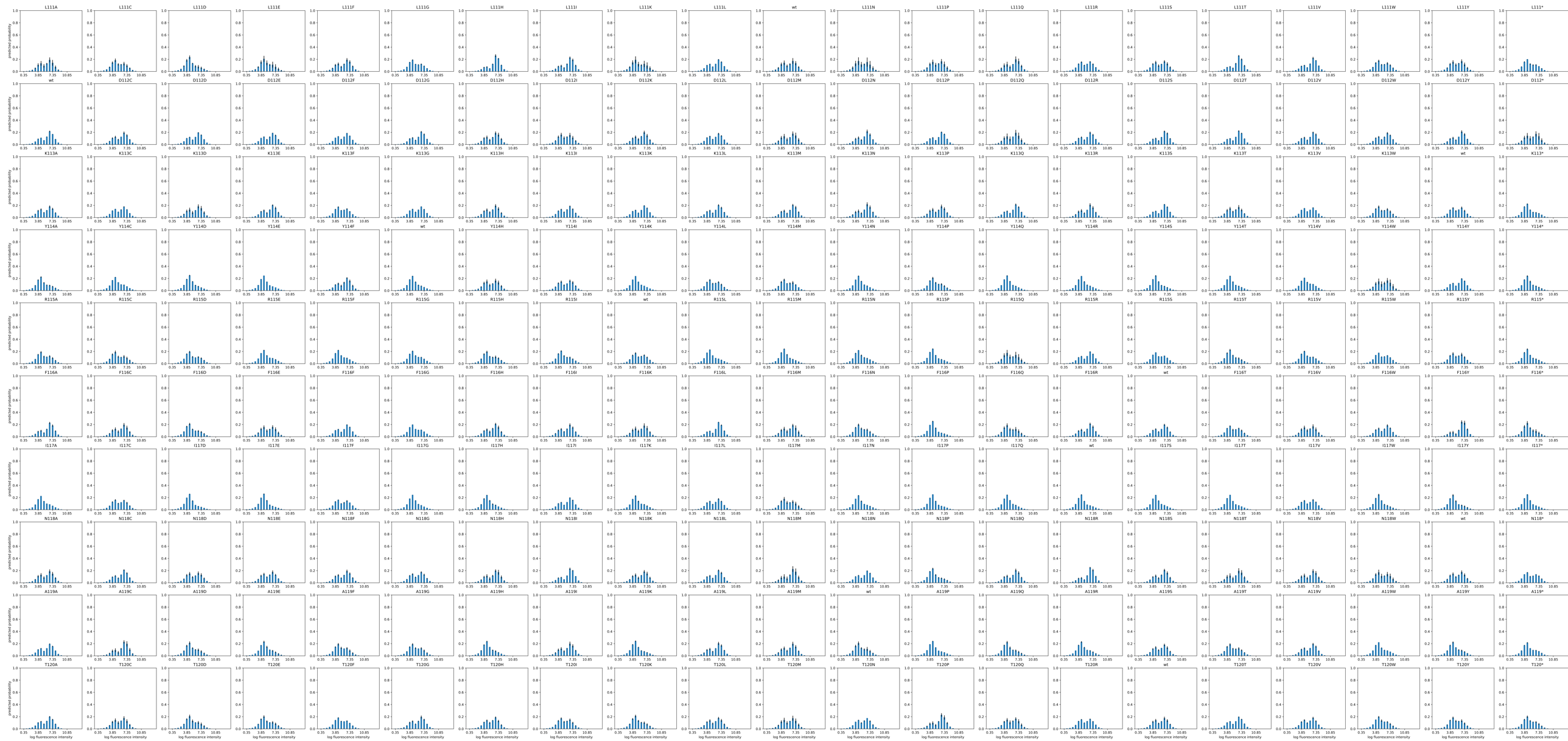

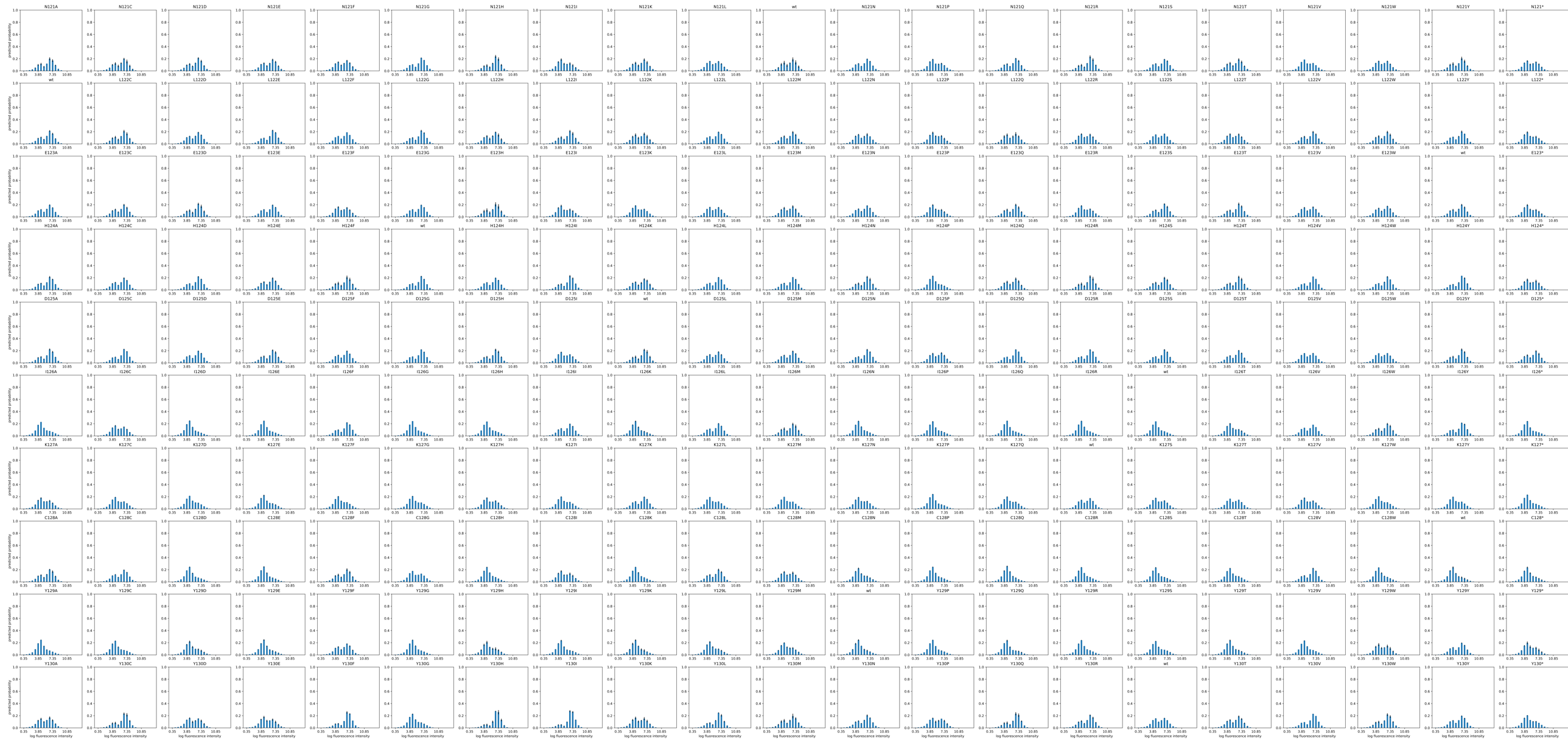

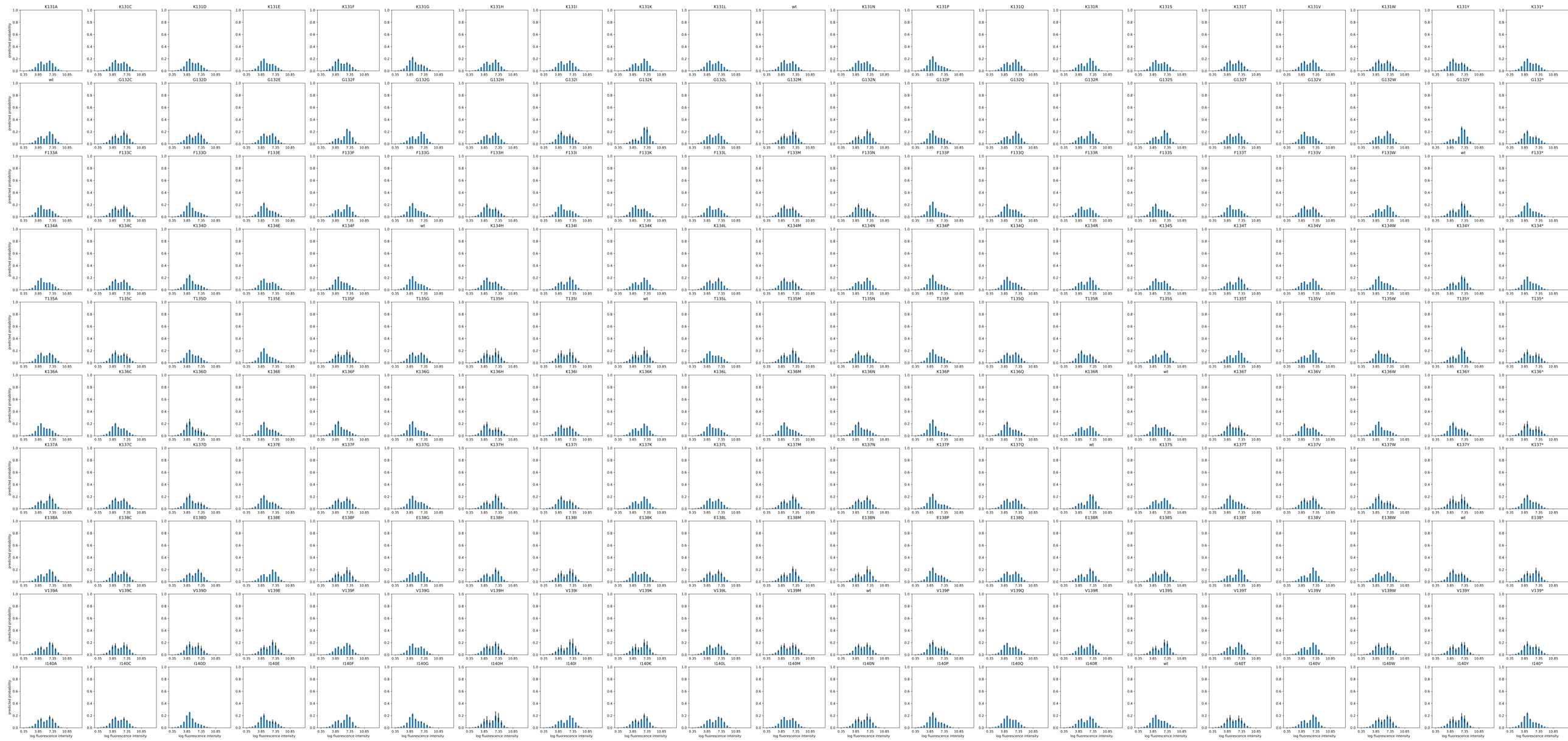

Supplement: gkae1052_Supplemental_Files [file gkae1052_supplemental_files.zip › Supplementary data 6.pdf]
